# Tree identity shapes composition, substrate shifts dominance in deadwood fungi of an old-growth lowland beech forest

**DOI:** 10.3897/imafungus.17.190198

**Published:** 2026-08-24

**Authors:** Glen Dierickx, Kis Vandekerkhove, Peter Van de Kerckhove, Pieter Asselman, Annemieke Verbeken

**Affiliations:** 1 Research Group Mycology, Ghent University, Ghent, Belgium Research Group Mycology, Ghent University Ghent Belgium; 2 Research Institute For Forest And Nature, Geraardsbergen, Belgium Research Institute For Forest And Nature Geraardsbergen Belgium

**Keywords:** Beta-diversity, coarse woody debris, community assembly, eDNA, fine woody debris, long-read sequencing, monitoring, species hypothesis

## Abstract

Wood-inhabiting fungal (WIF) communities are strongly structured by host tree species and environmental gradients. However, how WIF communities are structured among deadwood substrates when such drivers are minimized remains less clear, as do the implications for eDNA-based monitoring. We addressed these questions in a mono-dominant and unmanaged old Beech (*Fagus sylvatica*) forest, using long-read community profiling. Sampling followed a cross-fraction, tree-linked design that linked four deadwood substrate types to their source tree: logs, snags, attached fine woody debris (aFWD), and fallen fine woody debris (fFWD).

Across three analytical scopes, we quantified (1) stand-scale substrate structuring, (2) within-substrate variation, and (3) directed cross-substrate transitions. Alpha diversity was comparable across all substrates, but total richness did not approach saturation. In contrast, dominant diversity (Hill q_2_) saturated rapidly. Communities were strongly turnover-driven, indicating species replacement rather than nestedness. Tree identity explained a consistent fraction of compositional variance at the stand scale and remained the strongest predictor within coarse woody debris. In fine woody fractions, tree-identity effects weakened and substrate characteristics gained relative importance. Substrate differentiation was stronger in abundance-weighted than in presence–absence space, indicating that substrates primarily reorganize dominance within partially shared species pools.

These results show that tree-level legacy persists across fragmented deadwood fractions, while substrate context modulates competitive outcomes. Given these patterns, long-read eDNA efficiently captures dominant and recurrent components of WIF communities, whereas recovery of total richness is unrealistic under typical sampling effort. Monitoring should therefore prioritize sampling many trees with pooled cores per tree and broad coverage of substrate types and characteristics, including decay stage, size, and depth gradients, rather than intensive within-object stratification.

## Introduction

Deadwood is a key structural and functional component of forest ecosystems and contributes to nutrient cycling and the maintenance of habitat heterogeneity (Stokland et al. 2012). Wood-inhabiting fungi (WIF) are the principal drivers of wood decay and facilitate access to wood resources for many other organisms that depend on deadwood (Boddy 2001). Variation in deadwood size, decay stage, and exposure supports diverse saproxylic assemblages, while structural heterogeneity within the deadwood substrate pool is likewise associated with increased fungal diversity (Parisi et al. 2018; Yang et al. 2021). Moreover, WIF represents one of the most species-rich groups in forested ecosystems, contributing a unique fraction to forest biodiversity (Niskanen et al. 2023; Fjelde et al. 2025). Because deadwood is among the forest structures most strongly altered by management, WIF are increasingly recognized as indicator taxa in monitoring and conservation frameworks (Christensen et al. 2005; Heilmann-Clausen et al. 2014; Halme et al. 2017; Burrascano et al. 2021). Understanding how WIF communities are structured within and among deadwood fractions is therefore central to both deadwood ecology and evidence-based forest biodiversity monitoring.

Several recurrent drivers of WIF community structure have been identified. Host tree species is often the primary substrate-level filter, reflecting differences in wood chemistry, anatomical traits, and associated microbial interactions (Purahong et al. 2018; Lepinay et al. 2021; Odriozola et al. 2021). Decay stage represents a second major axis of variation, as it captures progressive shifts in wood chemistry, density and moisture during decomposition (Kahl et al. 2017). Deadwood size and quality further structure communities, since larger deadwood pieces buffer microclimatic fluctuations and provide higher structural heterogeneity than small-diameter fractions (Küffer et al. 2008; Abrego and Salcedo 2013). However, these deterministic gradients explain only part of the pronounced piece-to-piece variability characteristic of deadwood fungi. Wood is colonized sequentially, and early-arriving species can modify the substrate or exclude later colonists, producing priority effects. As a result, communities that differ in their assembly histories tend to diverge, leading to pronounced compositional differences among individual deadwood units (Fukami et al. 2010; Ottosson et al. 2014; Hiscox et al. 2018; Lepinay et al. 2021).

A key issue is that deadwood studies often focus on one specific segment of the deadwood substrate, most often coarse woody debris (CWD) in the form of logs (Fjelde et al. 2025). In reality, tree death results in a mosaic of deadwood fractions, physically continuous or not, that differ in diameter, microclimate exposure and colonization dynamics. For example, standing deadwood (snags) experience reduced soil contact and steep vertical moisture gradients while fine woody debris (FWD) is fragmented, rapidly decomposing, and more exposed to microclimatic variations (Pouska et al. 2016; Brabcová et al. 2022; Uhl et al. 2022; Dyson et al. 2026). Both fruitbody and metabarcoding surveys indicate that excluding FWD ignores a substantial part of the WIF community and richness, as small diameter fractions host distinct assemblages with strong turnover among pieces (Nordén et al. 2004; Juutilainen et al. 2011; Atrena et al. 2020; Brabcová et al. 2022; Korhonen et al. 2024). Still, cross-fraction designs that explicitly link substrates to their source tree are sparse.

Spatial scale further complicates study design and community analyses because WIF communities are highly compartmentalized within individual deadwood pieces. Turnover at centimeter- to meter-scale and depth stratification are common, creating distinct microhabitats even within a single log (Kubartová et al. 2012; Leonhardt et al. 2019; Dahlberg et al. 2025). This is particularly relevant for studies and surveys using metabarcoding. A single deadwood sample for metabarcoding, typically a small drill core, captures only a limited fraction of the community mosaic. Consequently, differences among samples may reflect both within-piece heterogeneity and turnover among different pieces. This creates a trade-off between replication within and across deadwood units and complicates decisions about how samples should be pooled without obscuring meaningful variation (Tedersoo et al. 2022; Bosch et al. 2023).

On the other hand, eDNA-based community profiling is well suited to study WIF communities because it detects most taxa present regardless of fruitbody formation and allows standardized comparisons of fixed volumes across dispersed substrates (Ovaskainen et al. 2013; Purahong et al. 2019). Long-read ITS sequencing further improves taxonomic resolution and comparability through reference frameworks such as UNITE Species Hypotheses (Tedersoo and Anslan 2019; Furneaux et al. 2021; Abarenkov et al. 2024).

In this study, we examine WIF communities, using eDNA sampling, in a long unmanaged, mono-dominant stand of European beech (*Fagus sylvatica*) with minimal environmental gradients. This setting provides near-null conditions to clarify how tree identity and within-substrate contrasts structure fungal communities. Because it remains unclear whether substrate differentiation overrides or preserves tree-level legacy effects, we apply a multi-substrate sampling design within a tree-identity framework that links deadwood fractions to their source tree. We compare four common, field-accessible substrates: logs, snags, attached fine woody debris (aFWD) and fallen fine woody debris (fFWD).

Our analyses aim to: (1) quantify the relative importance of substrate and tree identity at the stand scale, (2) evaluate within-substrate community structuring, and (3) assess community shifts across common substrate transitions. These results will enhance our understanding of community structuring in WIF, and be of use both for fundamental fungal ecology and conservation efforts. Finally, we also translate observed community patterns into practical guidance on how to allocate eDNA sampling effort.

## Materials

### Sampling design

Sampling took place in the Joseph Zwaenepoel forest reserve, Belgium, in an Atlantic old-growth lowland *Fagus sylvatica* (L.) stand. The stand has been set-aside since 1983 and holds dead wood volumes of more than 120 m^3^ ha^–1^ (Thomaes et al. 2024). Site context is given in detail in Vandekerkhove et al. (2018) and De Keersmaeker et al. (2023). The forest is characterized by a single dominant tree species, minimal environmental gradients, and no recent disturbance. This allows for a specific sampling design emphasizing how tree identity and substrate contrasts structure wood-inhabiting fungal (WIF) communities.

**Figure 1.**
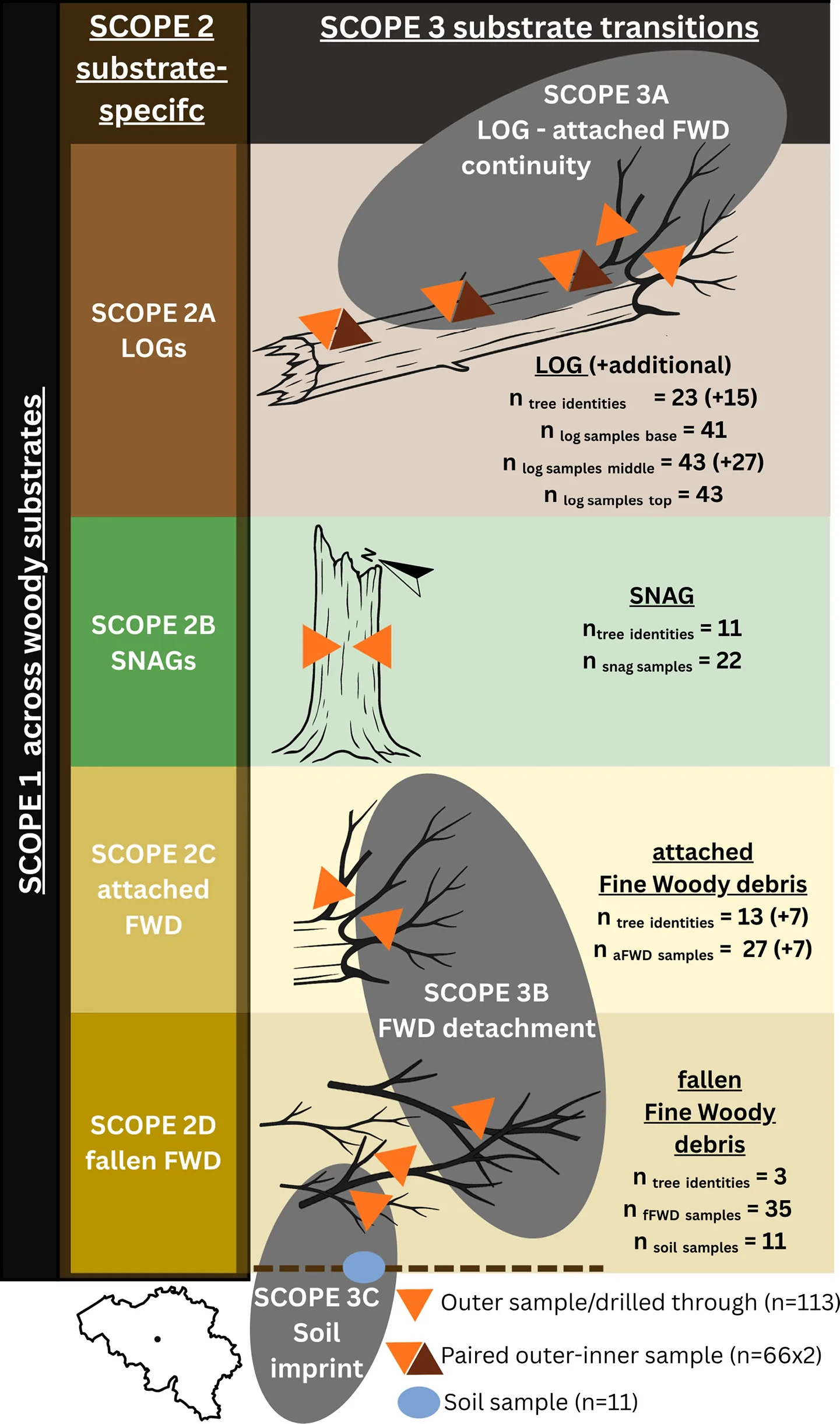
Overview of sampling design, realized samples and three analytical scopes used to disentangle substrate fungal community patterns in European Beech deadwood. Scope 1 targets diversity patterns across woody substrates, Scope 2 quantifies substrate-specific diversity within deadwood types and Scope 3 tests cross-substrate transitions linking substrates. The inset map (bottom left) indicates the sampling site.

We sampled sawdust with a wood auger from each selected deadwood object (Suppl. material 1: fig. S1, Appendix 1 (Sampling protocol)). Target substrates for sampling involved (A) logs (>15 cm diameter), (B) snags, (C) attached fine woody debris (aFWD, branches 5–15 cm diameter attached to a sampled log) and (D) fallen FWD (fFWD, branches in the crown of a sampled log). The sampling scheme is summarized in Fig. 1. To account for shared origin, traceable deadwood fractions originating from the same, once living, tree were linked by a shared tree legacy identifier called “tree identity”. Logs were selected to span Renvall decay stages 1–5 (Renvall 1995) and a broad size range (15–118 cm diameter). For each focal log, we checked whether an associated snag attributable to the same tree identity was present and, when present, sampled it (Renvall stages 1–4, 15–112 cm diameter). Likewise, when aFWD was present on the focal log, we sampled two distinct aFWD pieces (Renvall stages 1–4, 5–14 cm diameter). For fFWD, sampling was restricted to three coarse logs (94–110 cm DBH) in early decay with large crowns. Per crown, we sampled 12 fFWD pieces (2–9 cm diameter) and stratified by decay class. Because decay stage determination is less precise for fFWD due to faster decomposition rates, we grouped pieces into two contrasting field-based categories (early vs late decay) and omitted pieces with intermediate decay stage (see also Appendix 2).

For each sample, we pooled sawdust from two wood auger cores taken approximately 5 cm apart. Each sample therefore represents a composite of two replicate cores. Standard drilling depth was 0–8 cm under bark (Suppl. material 1: fig. S1). On logs, we sampled three longitudinal positions (base, middle, top) and, at each position, collected paired depth strata: a standard outer sample from 0–8 cm and an extra inner sample from 10–18 cm under bark. Snags were drilled at breast height on the north and south cardinal positions. For FWD, when pieces were < 8 cm in diameter, the core spanned the full diameter (bark excluded). While this somewhat biases equal sample volume, in situ, no volumes are the same, because wood decay creates volumetric inconsistencies and DNA extractions are made from a limited subsample of homogenized wood powder. We recorded substrate type, tree identity, decay stage at drilling point (Renvall stages 1–5), sampling depth, deadwood diameter at drilling point, log position, and for snags cardinal direction (north or south). To test for local forest-floor impact, shallow soil samples (organic horizon) were collected directly beneath selected fFWD pieces, enabling direct comparison of community similarity between paired fFWD–soil samples. The detailed sampling protocol can be found in Appendix 1 and Suppl. material 1: fig. S1.

This strategy resulted in the following realized totals for analysis. On 23 logs we attempted the full three-position by paired depth design and obtained 127 log samples, with n_base_ = 41 (22 outer, 19 inner), n_middle =_ 43 (22 outer, 21 inner), and n_top_ = 43 (23 outer, 20 inner). Inner sampling was skipped at positions where it would have reached far beyond the pith or where wood was too degraded to make a meaningful contrast. To cover more trees, we sampled 15 additional logs at the middle position only and obtained 27 extra log samples (16 outer, 11 inner). The extra outer sample reflects one tree where separate white- and brown-rotted middle-outer fractions were sampled. Across the combined log sets, aFWD yielded 34 samples (27 and 7 from additional logs). 11 snags contributed 22 samples (one per cardinal direction). For fFWD sampling resulted in 35 samples (spread across 3 trees, half in early and half in late decay). Soils directly under selected fFWD pieces contributed 11 samples.

### Wet lab

Wood shavings were homogenized under liquid nitrogen. Two extraction replicates per sample were carried out and then pooled following Dierickx et al. (2024). In short, 0.5 mL homogenized wood powder was suspended in 800 µL of 2× CTAB extraction buffer containing 5 µL β-mercaptoethanol and 2 µL Proteinase K, and incubated at 55 °C for 2 h. The lysate was purified with two rounds of chloroform:isoamyl alcohol (24:1) cleanups, precipitated using isopropanol (0.54×) and 7.5 M ammonium acetate (0.08×), washed with 70% ethanol and resuspended in sterile water. Extracts were cleaned using 0.8X SPRI beads (Beckman Coulter, Pasadena, CA, USA) and diluted to 1 ng/µl.

Sequencing was carried out for four libraries as shown in Table 1. For details on libraries I and II we refer to Dierickx et al. (2026, submitted). In this study, gDNA was amplified in duplicate with primer pair ITS1f/ITS4 (White et al. 1990; Gardes and Bruns 1993). Samples underwent 25 amplification cycles in 25 µl reactions using Platinum™ SuperFi II Green PCR Master Mix according to the manufacturer’s recommendations (Invitrogen, Waltham, MA, USA), then a 0.9× SPRI cleanup and were quantified on a Qubit fluorometer (Invitrogen, Carlsbad, CA, USA). Equimolar pools were barcoded with ONT’s Native Barcoding Kit V14 following the manufacturer’s recommendations (SQK-NBD114.96, Oxford Nanopore Technologies Inc., Oxford, UK). Negative (extraction blank carried into PCR) and positive (mock community) controls were implemented throughout. Two libraries of 93 and 89 samples were sequenced on a PromethION flow cell at the NXTGNT facility, Faculty of Pharmaceutical Sciences, Ghent University (Belgium). Pod5 files were basecalled and demultiplexed (--barcode-both ends) using Dorado v0.9.1 in SUP mode (Oxford Nanopore Technologies 2024). Data is available at SRA (PRJNA1428556).

**Table 1.** Sequencing libraries included in this study. Four ONT amplicon libraries, showing primer pair, amplification protocol (UMI [Unique Molecular Identifier] vs standard amplicon), sample number, sequencing device and SRA project number. Library and sequencing depth were used as technical covariates in downstream analyses.

Library	Dataset	Primer pair	Amplification protocol	Samples	Device	SRA
MI	Dierickx et al. (2026, submitted)	ITS9mun ITS4	UMI - amplicon	24	MinION	PRJNA1328363
P1	Dierickx et al. (2026, submitted)	ITS1f ITS4	UMI - amplicon	75	PromethION	
P2	this study	ITS1f ITS4	amplicon	93	PromethION	PRJNA1428556
P3	this study	ITS1f ITS4	amplicon	89	PromethION	

Sequencing data for the remaining samples (24+75) were sourced from Dierickx et al. (2026, submitted) and available at SRA (PRJNA1328363). The data differs in the amplification method, using unique molecular identifiers (UMIs), but similarly targeted the full-length ITS region using the same set of primers for 75 samples and the ITS9mun/ITS4ngs primer pair (Tedersoo et al. 2018) for the remaining 24. Library and sequencing depth were used as technical covariates downstream.

### Bioinformatics

Demultiplexed fastq files were fed into and preprocessed with eNano (Dierickx et al. 2024) into per-sample fasta files with standard settings. In short, adapters and primers were trimmed, and reads were reoriented. A low Phred-based quality filter was set (--q 10) to remove the most spurious reads. Preprocessed reads were taxonomically annotated to Species Hypothesis (SH) as implemented in the UNITE v.10 database (Abarenkov et al. 2024) using the SH-matching algorithm (Abarenkov et al. 2022). Per read annotations were summed into a SH-table using 1.5% SHs and all analyses are based on this threshold.

In total, eleven samples failed quality criteria (low read depth, cross-contamination, or host dominance) and were excluded from analysis, together with six control samples. We further removed non-fungal lineages, lab contaminants (*Malassezia* spp. detected in the negative control) and set SH minimum abundance to 10 reads at the sample level. This threshold was set from the positive control barcode hopping signature (barcode hopping showed ≤ 8 reads per SH from the positive control in a few other samples), adding a small surplus that also removes unstable single- and doubletons while preserving genuine low-abundance taxa. After filtering, 264 samples (incl. 11 soil samples) remained for downstream analyses. Per sample metadata is given in Suppl. material 2: table SS1.

### Analyses

#### Scopes

Analyses were structured to contrast fungal richness and community composition within and across four deadwood substrates (LOG, SNAG, aFWD, fFWD).

**Scope 1: Stand-scale among-substrate structuring**. We combined all woody substrates in an across-substrate comparison to quantify how substrate type structures the sampled deadwood community.

**Scope 2: Substrate-specific patterns**. To quantify within-substrate variation in diversity, we analyzed each substrate type separately and evaluated how richness and community composition varied with substrate characteristics (e.g. decay stage and diameter), while accounting for tree identity. The scopes are referred to per substrate type as: (2A) LOG, (2B) SNAG, (2C) aFWD, and (2D) fFWD.

**Scope 3: Cross-substrate transitions**. To assess how substrates are interlinked, we contrasted three cross-substrate transitions. First, in (3A) we contrast LOG versus aFWD to assess trunk-branch continuity of communities across contrasting wood diameters within a continuous deadwood unit. Second, in (3B) we contrast aFWD versus fFWD to quantify shifts after branch detachment, using both a global comparison across all trees with either aFWD or fFWD data and a paired subset restricted to the three trees contributing both FWD types. Third, in (3C) we contrast fFWD-soil pairs to evaluate local soil impact on fFWD using soil cores collected directly underneath the corresponding fFWD pieces.

Together, this design separates broad substrate effects (Scope 1) from within-substrate drivers (Scope 2) and from physically defined cross-substrate transitions (Scope 3).

#### Analysis framework (applies to Scopes 1–3)

All analyses were structured to follow the same logic across scopes. We combined alpha diversity modelling (Scopes 1 and 2) with a unified multivariate pipeline overall (Scopes 1–3). We based multivariate analyses on two complementary distances to demonstrate robustness: robust Aitchison distances on SH abundance (compositional, rare taxa downweighted) and Jaccard distances on presence-absence (all taxa treated equally). Scope-specific predictors and sample/SH totals are summarized in Table 2, with predictor encoding in Appendix 2 and scope-specific procedures in Appendix 4.

**Table 2.** Predictors by analysis scope with sampling and Species Hypothesis (SH) totals. • Predictors in both alpha-diversity modelling and PERMANOVA. ○ Predictors used only in PERMANOVA. NULL indicates predictors evaluated with design-preserving nulls (see Appendices 3, 4). 2 × n denotes paired samples.

**Predictor Scope**	**Technical**		**Tree identity**†	**Substrate characteristics**					**Trees** ^‡^	**Samples** ^‡^	**SHs** ^‡^
	**log (Reads)**	**Library**		**Substrate type**	**Decay stage**	**Depth**	**Position**	**Size**			
1. across types	•	•	•NULL	• NULL	/	/	/	/	39	253	1801
2A. LOG	•	•	• NULL	/	•	•	•	•	23	127	1187
2B. SNAG	•	•	• NULL	/	•	/	•		11	22	548
2C. aFWD	•	•	• NULL	/	•	/	/	•	20	34	536
2D. fFWD	•	•	• NULL	/	•	/	/	•	3	35	673
3A. LOG vs aFWD	○	○	○ NULL	○	/	/	/	/	13	63	894
3B. aFWD vs fFWD	○	○	○ NULL	○ NULL	/	/	/	/	20|3	69|41	864|740
3C. fFWD vs soil	○	N.A.	N.A.^§^	○ NULL	/	/	/	/	3	2 × 11	2354

†Tree identity is tested in unblocked PERMANOVA models and used as the permutation stratum in blocked PERMANOVA models. ‡g|r denotes global and tree-identity-restricted analysis for aFWD-fFWD scope 3B. § tree identity included indirectly by blocking permutations by fFWD-soil pairs.

Tree identity was used throughout to account for non-independence among samples with shared history. In PERMANOVA we used unblocked models with sequential tests to partition variance among technical covariates, tree identity and scope-specific predictors, and blocked models with permutations stratified by tree identity using marginal tests to assess substrate characteristics while controlling for tree identity. Multivariate dispersion was assessed with PERMDISP as a diagnostic.

Because tree identity and substrate type are multi-level grouping factors with uneven replication, PERMANOVA *R*^2^*_observed_* for those terms can absorb substantial variance and become sensitive to design imbalance and heterogeneous dispersion. We therefore benchmarked tree identity (throughout) and substrate effects (Scopes 1 and 3) against design-preserving null models by shuffling labels while preserving group sizes. For each shuffle, we recomputed PERMANOVA R^2^ under the same model and permutation scheme and summarized effects as: the mean-corrected effect ∆*R*^2^ = *R*^2^_*observed*_*– mean* (*R*^2^_*null*_), the standardized effect size SES = ∆*R*^2^/*SD*(*R*^2^_*null*_) and the empirical p-value as the proportion of permutations where *R*^2^_*null*_ ≥ *R*^2^_*observed*_. Robustness was further assessed using a sensitivity analysis (details in Appendix 3) consisting of leave-one(-tree)-out PERMANOVA and within-versus-between group separation metrics (AUC and ANOSIM), visualized with ECDFs. Appendix 3 details the sensitivity analyses and null models, and Appendix 4 contains per-scope implementation details. To further assess robustness to amplification protocol, Scope 1 and Scope 2A analyses were repeated separately for UMI and non-UMI libraries (Appendix 6).

##### Scope 1: Stand-scale among-substrate structuring

Broad alpha-diversity contrasts between substrates were assessed with sample-based iNEXT curves for Hill numbers q_0_ (species richness), q_1_ (exponential of Shannon entropy, typical diversity), and q_2_ (inverse Simpson diversity, dominant diversity). Pairwise differences in q_2_ were evaluated at the minimum shared sample coverage across groups (C*) without extrapolation using z-tests with bootstrap SEs. To assess within-tree species accumulation, we constructed tree-level iNEXT curves for trees with ≥ 6 samples. To obtain model-based sample-level contrasts among substrates, we modelled observed richness (number of 1.5% SHs) using negative-binomial GLMMs with log(reads) and library as fixed covariates and tree identity as a random intercept. Pairwise substrate contrasts were evaluated with Tukey adjustment for multiple comparisons.

We analyzed community composition with PERMANOVA to quantify contributions of substrate type, tree identity, and technical covariates, including dispersion checks and null benchmarking as described above. Incidence-based β-diversity was decomposed into turnover and nestedness using betapart, and turnover:nestedness (T:N) ratios were summarized for within-group versus between-group contrasts for both substrate groups and tree identity groups. Here, T:N was defined as β_SIM_/β_SNE_, with values approaching 0 indicating nestedness-dominated dissimilarity and values >>1 indicating turnover-dominated dissimilarity. We quantified sample (LCBD) and species (SCBD) contributions to β-diversity and visualized dominant SCBD taxa together with their abundance and prevalence across substrates. For ordination-based visualization of stand-level community composition, we used a partial PCA on CLR-transformed abundances (Aitchison framework, zeros imputed prior to transformation), partialling out log(reads) and library effects so axes represent residual compositional structure. Sensitivity analysis was run as specified earlier. Occupancy frequency distributions were additionally produced to show prevalence structure across samples, logs, and tree identities.

##### Scope 2: Substrate-specific drivers within each deadwood type

Analyses were conducted separately for logs (Scope 2A), snags (Scope 2B), attached fine woody debris (aFWD, Scope 2C), and fallen fine woody debris (fFWD, Scope 2D). Alpha diversity (observed richness, number of 1.5% SHs) was modelled as in Scope 1 using negative-binomial GLMMs, but with substrate-specific predictors (Table 2, formulas in Suppl. material 2: table S6) and fitted separately within each substrate. For each substrate, we also report the three most abundant SHs to aid interpretation.

Within each substrate, community composition was analyzed using the general multivariate framework with PERMANOVA, betapart turnover/nestedness (T:N ratios), and within-versus-between separation metrics (sensitivity analyses: AUC, ANOSIM, ECDF, Appendix 3). Importantly, for logs (Scope 2A), the paired inner–outer design was treated as a defined sub-scope to test depth stratification (INNER versus OUTER), with implementation details provided in Appendix 4.

##### Scope 3: Directed cross-substrate transitions

To minimize collinearity among substrate type and substrate characteristics, only substrate type was included as substrate predictor in this scope (Table 2). Analyses followed the common framework. Pairing logic and the exact contrast and null-model structure for each sub-scope are detailed in Appendix 4.

All analyses were conducted in R 4.4 (R Core Team 2024) using packages vegan (Oksanen et al. 2025), betapart (Baselga et al. 2025), iNEXT (Chao et al. 2014; Hsieh et al. 2025), permute (Simpson 2025), lme4 (Bates et al. 2015) and emmeans (Lenth and Piaskowski 2025). Plots were made using ggplot2 (Wickham 2016; Wickham et al. 2019). All code is available at GitHub/Mycomatics/scripts/Dierickx_Vandekerkhove_Verbeken_2025.

## Results

Sequencing resulted in 222.7 million reads. Of the retained reads, 74.0% could be annotated to existing fungal UNITE SHs. After sample exclusion and per-sample abundance filtering, 2358 SHs and 264 samples were retained. The main results from null models are shown in Table 3. Full results are provided in Suppl. material 2: tables S2–S9: S2 PERMANOVA, S3 null models and sensitivity metrics, S4 leave-one-out robustness, S5 multivariate dispersion, S6 alpha models and S7 emmeans, S8 fFWD–soil nulls and S9 betapart fractions. Null model and group separation distributions are visualized in Appendix 5, and amplification protocol-stratified robustness analyses are provided in Appendix 6. While an in-depth taxonomic discussion is beyond the scope of this study, we provide as supplementary material Krona wheel charts illustrating overall taxonomic composition and substrate-specific composition. Additionally, betapart fractions (T:N ratios) are visualized in Suppl. material 1: fig. S2, and occupancy frequency distributions are shown in Suppl. material 1: fig. S3.

**Table 3.** Null-corrected PERMANOVA effect sizes (ΔR^²^) and within–between separation (AUC) for tree identity and substrate type across scopes and distance metrics (robAit = robust Aitchison). Significance is indicated as *** p < 0.001, ** p < 0.01, and * p < 0.05.

**Scope**		**Term**	**Metric**	**ΔR^²^**	**AUC**
1		tree identity	robAit	0.113***	0.522*
			Jaccard	0.098***	0.685***
		substrate type	robAit	0.081***	0.594***
			Jaccard	0.027***	0.619***
2	A	tree identity	robAit	0.172***	0.598***
			Jaccard	0.088***	0.740***
	B	tree identity	robAit	0.094	0.562
			Jaccard	0.101***	0.870***
	C	tree identity	robAit	0.084	0.655*
			Jaccard	0.033**	0.829***
	D	tree identity	robAit	0.019	0.535***
			Jaccard	0.040***	0.628***
3	A	tree identity	robAit	0.082*	0.545
			Jaccard	0.064***	0.709***
	B - global	tree identity	robAit	0.057	0.527
			Jaccard	0.046***	0.706***
		substrate type	robAit	0.008	0.518
			Jaccard	0.008***	0.651***
	B - paired	substrate type	robAit	0.007	0.606*
			Jaccard	0.011**	0.696*
	C	substrate type	robAit	0.364***	0.861***
			Jaccard	0.186***	0.994***

### Scope 1: Stand-scale among-substrate structuring

In this scope, we quantified how strongly substrate type and tree identity structure diversity at the stand scale.

**Figure 2.**
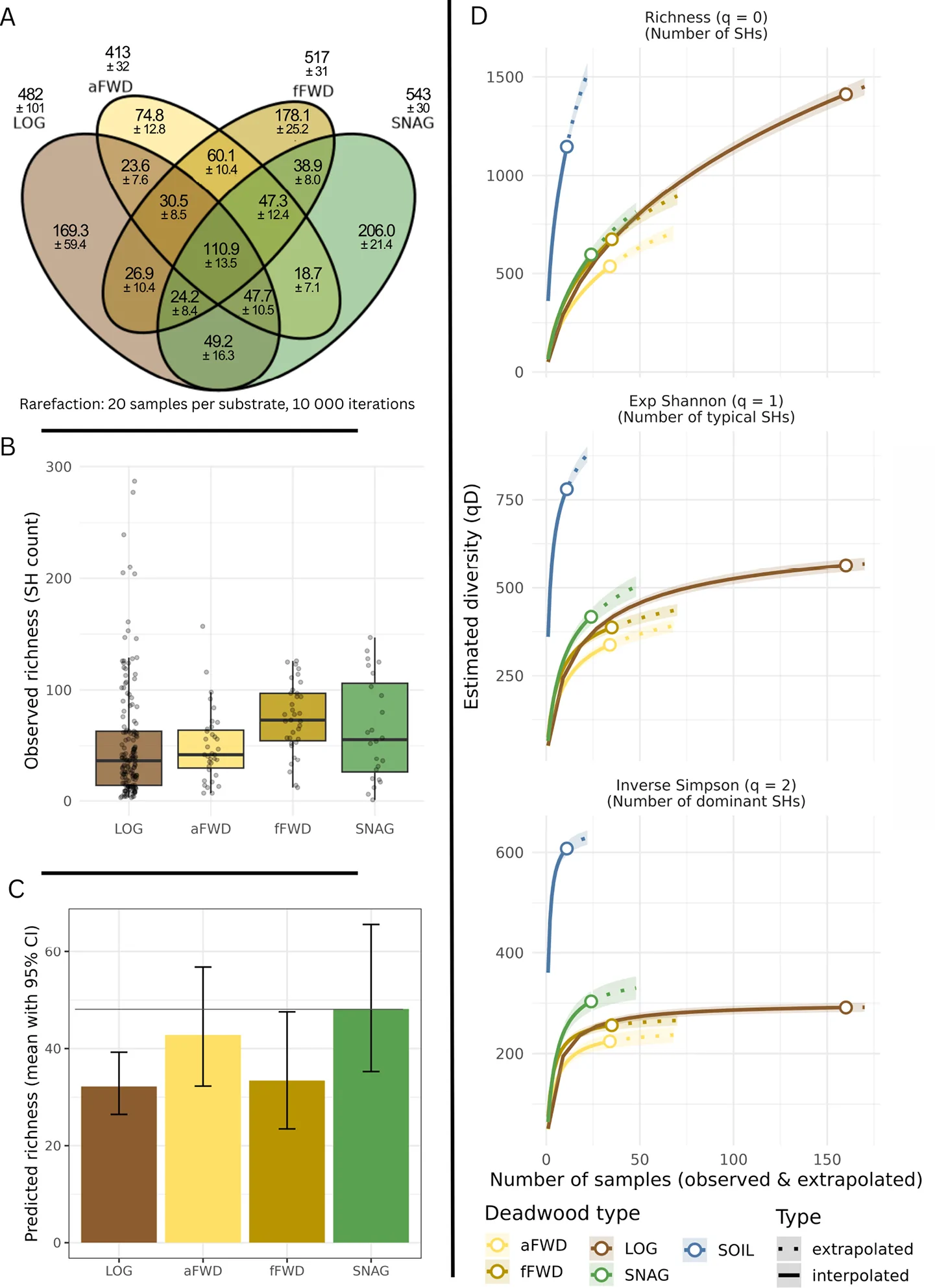
Scope 1: Alpha diversity per substrate type. **A** Venn diagram with Species Hypothesis overlap – SH aggregated per substrate type, standardized to compare 20 samples per substrate across 10k iterations **B** observed richness per sample **C** modelled richness per sample **D** collector's curves per Hill number, showing observed richness (q = 0), typical richness (q = 1) and dominant richness (q = 2). Colour shading = 95% confidence intervals. Abbreviations: attached fine woody debris (aFWD), fallen fine woody debris (fFWD).

Fig. 2 synthesizes alpha diversity patterns for the 1801 deadwood SHs.

Substrate-level SH pools overlapped broadly, with 205 SHs shared among all substrates. Observed per-sample richness spanned a wide range (1–287 SHs). Read-based rarefaction generally flattened within samples, indicating good within-library coverage (Suppl. material 1: fig. S4C). Sample-accumulation curves diverged by Hill order: richness (q_0_) did not reach an asymptote, whereas dominant diversity (q_2_) plateaued across substrates, showing saturation of common taxa but incomplete sampling of rare SHs. Across substrates, dominant diversity is of the same order of magnitude (±200–350 dominant SHs). At a common sample coverage of 81%, dominant diversity differed among categories, with SOIL highest and woody substrates ranking SNAG > LOG > fFWD > aFWD (q_2_ at C* = 0.81: 561, 303, 264, 238, 212; all pairwise contrasts p < 0.05). Tree-level accumulation curves (Suppl. material 1: fig. S5) indicate that dominant diversity (q_2_) saturated after > 5 samples per tree, whereas typical (q_1_) and total richness (q_0_) showed tree-specific saturation patterns and required more samples.

At the sample level, modelled richness (GLMM) differed only marginally between LOG and SNAG samples (p = 0.03), with a log sample averaging ~33% fewer SHs than snags. Technical covariates (library and sequencing depth) were consistently significant. These tendencies (SNAG ≥ FWD ≥ LOG) are visible both in the modelled estimates and in the coverage-standardized comparisons, but should be interpreted cautiously given incomplete q_0_ sampling and the smaller sample sizes for FWD and SNAG.

Across all woody samples, community composition was structured by both tree identity and substrate type. Tree identity explained more variance than substrate type in both metrics (ΔR^2^_RA_ = 0.113, ΔR^2^_JAC_ = 0.098, both p < 0.001), indicating that tree identity continues to imprint composition beyond substrate differences. Effects were generally stronger in abundance-weighted space than in presence–absence data, particularly for substrate type (ΔR^2^_RA_ = 0.081, ΔR^2^_JAC_ = 0.027, both p < 0.001). This suggests that while tree identity structures species composition broadly, substrate differentiation more strongly influences which taxa become abundant. Turnover dominated β-diversity and T:N ratios were much higher between substrates than within substrates (T:N_between_ = 11.1, T:N_within_ = 5.9), showing that cross substrate contrasts generate a strong taxon replacement signal in the dataset.

**Figure 3.**
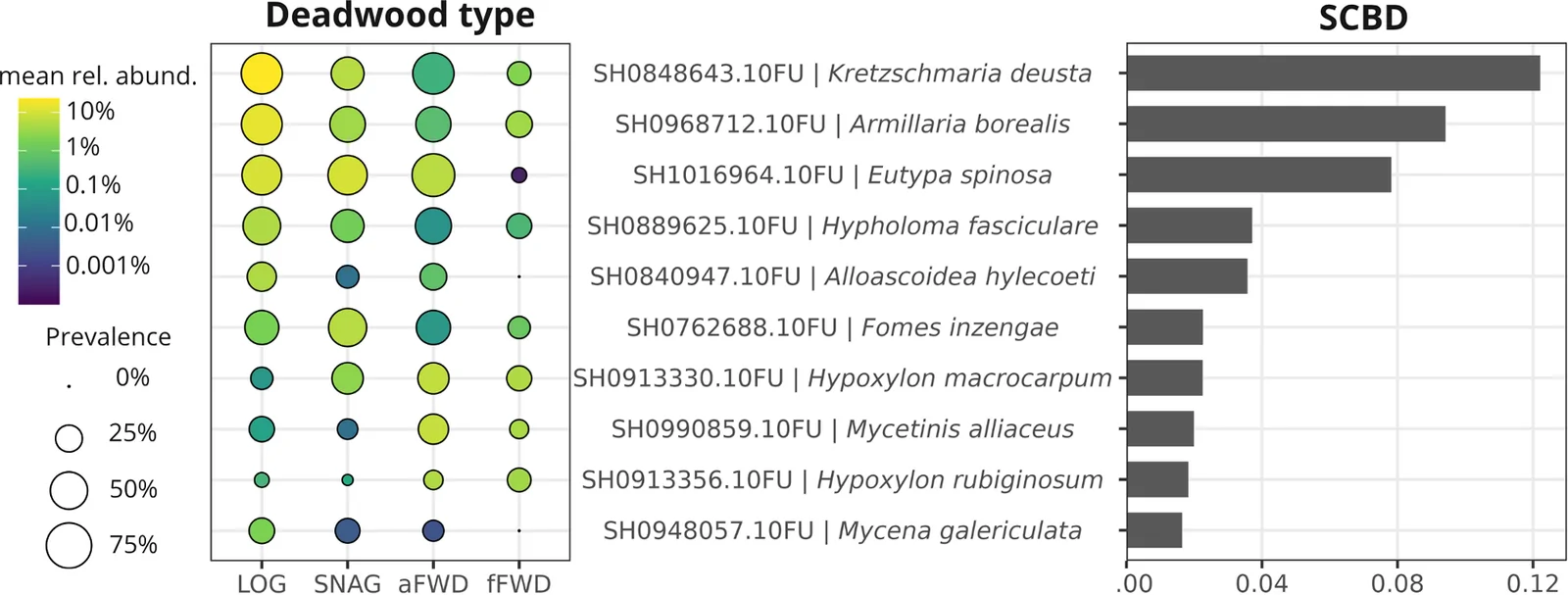
Distribution and contribution of shared fungal species hypotheses (SHs) across deadwood categories (LOG, SNAG, aFWD, fFWD). Left: dot heatmap showing SH distribution across categories, with fill indicating mean relative abundance (log scale) and point size denoting prevalence. Right: species contributions to β-diversity (Hellinger distance) for the same SHs shown in the heatmap, ranked by their contribution at the dataset scale. Abbreviations: attached fine woody debris (aFWD), fallen FWD (fFWD), Species Contribution to Beta Diversity (SCBD).

**Figure 4.**
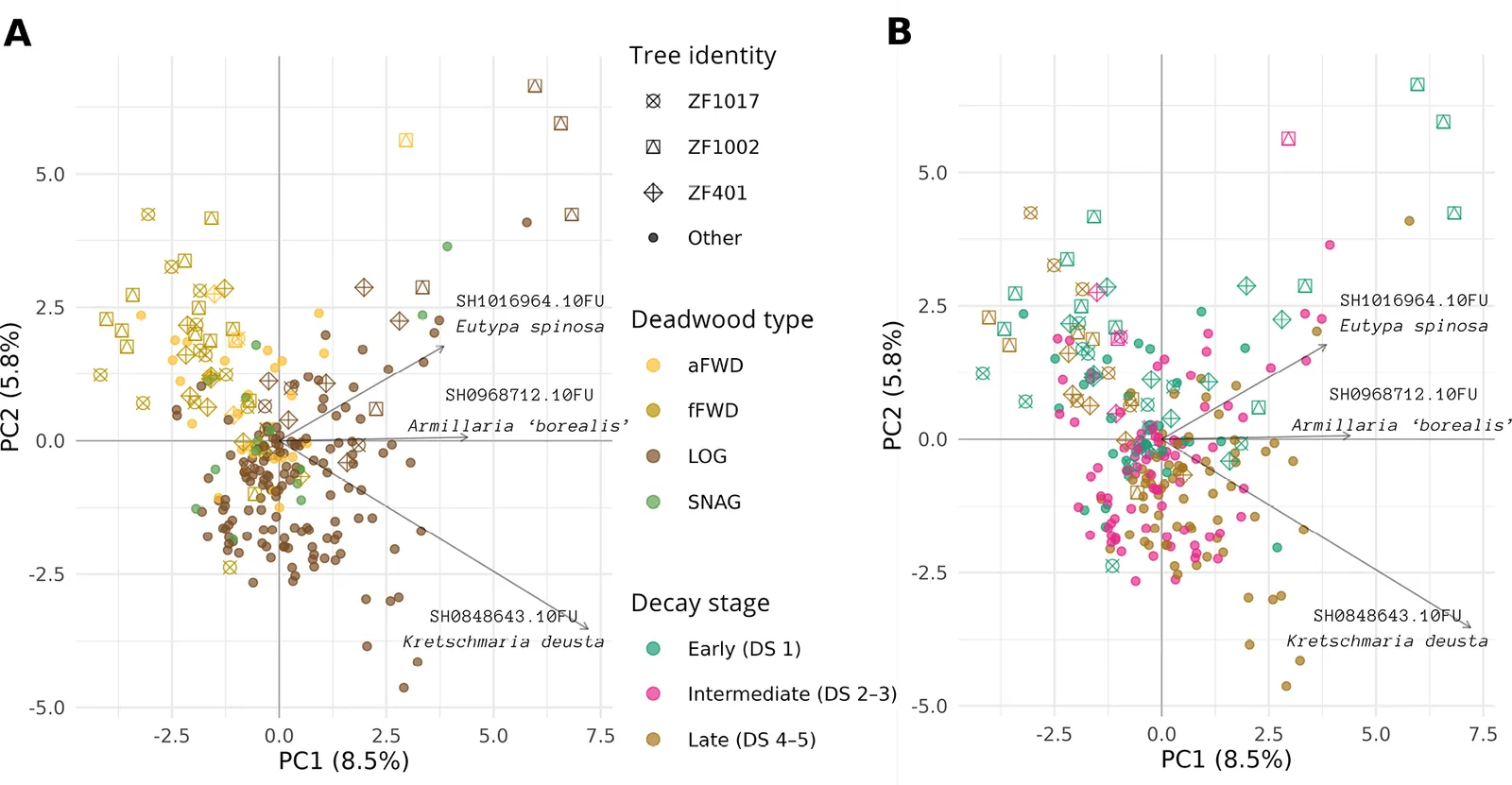
Partial PCA of fungal community composition across all samples (Scope 1), based on centered log-ratio (CLR)–transformed sequence counts with zero replacement (robust Aitchison framework). Effects of sequencing depth and library were partialled out (Condition(log(reads) + library)). Arrows indicate species loadings for the three UNITE Species Hypotheses (SHs) with the strongest contributions to among-sample compositional turnover. **A** Samples formatted by deadwood substrate type and **B** by decay stage. Abbreviations: attached fine woody debris (aFWD), fallen FWD (fFWD).

Species contribution to β-diversity highlighted three dominant and influential SHs annotated as *Kretzschmaria deusta* (SH0848643.10FU), *Armillaria borealis* (SH0968712.10FU), and *Eutypa spinosa* (SH1016964.10FU). They accounted for the largest share of β-diversity and were also the most abundant taxa in the dataset. Abundance and prevalence of the 10 most contributing (SCBD) SHs are visualized in Fig. 3. In the partial PCA (Fig. 4), axis 1 (8.5% variance) primarily discriminated logs from fine woody debris, with attached FWD intermediate and fallen FWD showing the strongest separation from logs. Axis 2 (5.8%) partially aligned with decay-related structure within logs but not within FWD. In contrast, SNAG samples did not form a distinct cluster on either axis. The three trees with the most samples (those that included fFWD) are formatted by a different symbol to reveal tree identity aggregation.

### Scope 2: Substrate-specific characteristics within each deadwood type

Within each substrate, we quantified whether tree identity and substrate characteristics (fragment diameter, decay stage, sampling depth) explain diversity and community composition.

#### Scope 2A. LOGS

Within logs, the most common SHs were *Kretzschmaria deusta* (SH0848643.10FU, 22.7%), *Armillaria borealis* (SH0968712.10FU, 14.6%) and *Eutypa spinosa* (SH1016964.10FU, 11.3%). Superficial log samples were substantially richer than deeper samples (70% higher richness, p < 0.001, Suppl. material 2: table S6). Decay stage was marginally significant for alpha richness (p = 0.07).

**Figure 5.**
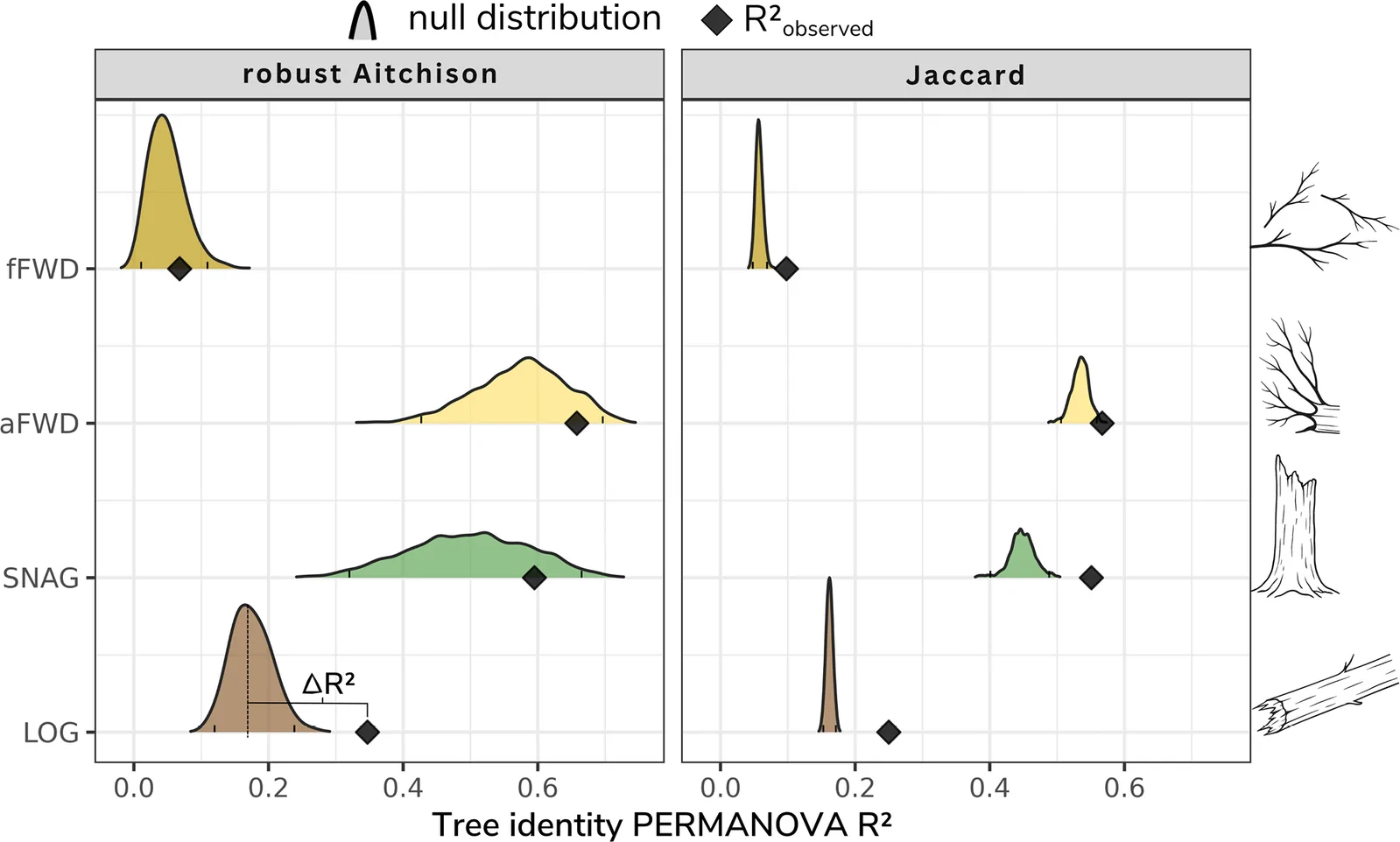
Scope 2. PERMANOVA Null distributions of R^²^ for tree identity effects on fungal community composition across scopes and distance metrics. Null distributions from tree-label shuffle permutations, with black diamonds denoting the observed R^²^ values from unblocked PERMANOVA and ΔR^²^ visually represented (bottom left). Abbreviations: attached fine woody debris (aFWD), fallen FWD (fFWD).

No spatial autocorrelation was detected among logs for either distance metric (both Mantel r < 0.02, p > 0.37). Tree identity was the dominant predictor of community composition. After correcting with tree-label shuffle null models, tree identity ΔR^2^ was 0.172 in robust Aitchison space and 0.088 in Jaccard space (Fig. 5, Table 3, Suppl. material 2: table SS3). Decay stage at the drill point was the strongest substrate characteristic (R^2^ = 0.034–0.098 across metrics) and was stable in LOO analyses for Jaccard but not for robust Aitchison, partly reflecting dispersion heterogeneity. Longitudinal position and diameter showed weak and inconsistent effects (R^2^ ≤ 0.02), whereas depth exhibited a small but stable effect in presence–absence space (R^2^ = 0.015). Sensitivity analysis confirmed these results, with within–between tree contrasts indicating higher similarity within than between trees that was modest in abundance-weighted space but clear in presence–absence (AUC_RA_ = 0.60, AUC_JAC_ = 0.74, both p < 0.001). β-diversity was turnover-dominated (T:N_between_ = 5.7, T:N_within_ = 4.9). Together these results indicate that log identity is the primary driver of fungal community composition, with within log substrate characteristic gradients adding a smaller but detectable component.

**Figure 6.**
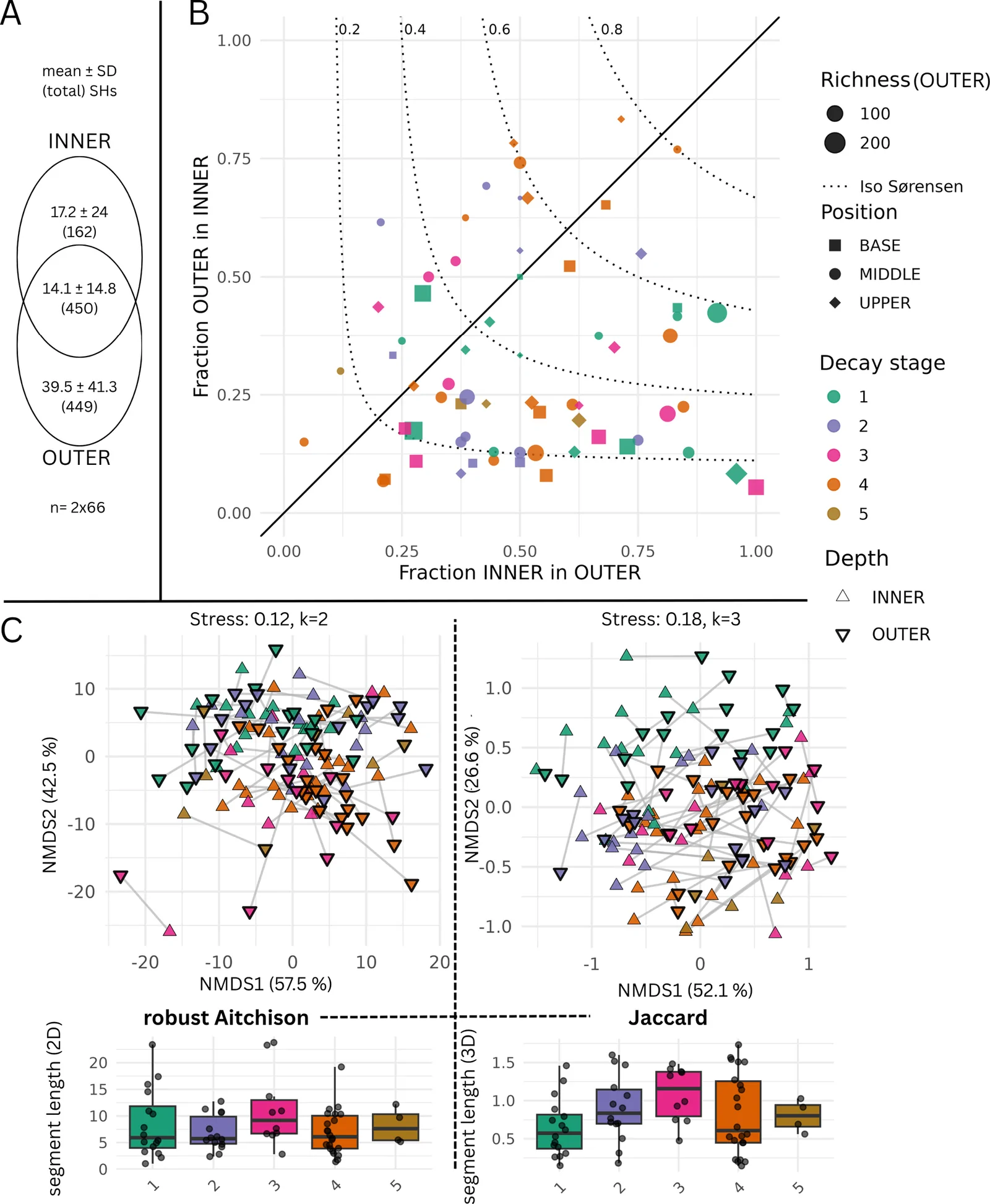
Scope 2A. Depth stratification between INNER and OUTER log samples. **A** Venn diagram of SH overlap between INNER and OUTER samples as average ± SD SHs per sample (total) **B** Nestedness scatterplot with iso-overlap guides based on Sørensen similarity (higher is more similar). Each point represents one INNER–OUTER sample pair from the same tree and position. Points clustering below the 1:1 line and toward the lower right indicate a tendency for INNER assemblages to be partially nested within the richer OUTER assemblages. Point size scales with OUTER richness, colours indicate decay stage, and shapes denote position **C** Upper: NMDS ordinations of INNER–OUTER sample pairs connected by grey lines and, lower: the distribution of those segment lengths (including NMDS3 for Jaccard) per decay stage based on robust Aitchison distances (left) and Jaccard distances (right). N = 2 × 66 samples.

Because inner and outer samples are spatially coupled, we checked whether one community is a partial subset of the other. Across all paired samples (n = 66 × 2), 162 (17.2 ± 24) SHs were unique to inner, 449 (39.5 ± 41.3) to outer and 450 were shared, indicating only partial overlap (Fig. 6A). Directional containment of INNER in OUTER increased with OUTER richness (Spearman ρ = 0.24, p = 0.02) and was negatively correlated with INNER richness (ρ = -0.30, p = 0.007). This pattern is in part due to higher richness in outer samples (paired Wilcoxon signed rank, V = 1709, p < 0.001), consistent with the GLMM result (IRR_outer_ = 1.70). This indicates that outer samples add taxa rather than simply replacing inner communities. A nestedness scatterplot (Fig. 6B) visually confirmed the asymmetry.

Partitioning β-diversity supported a mixed mechanism dominated by turnover but with a measurable nestedness component with T:N = 2.9. This suggests that most pairwise differences reflect replacement, but with INNER samples partially nested in OUTER ones. In ordination space (Fig. 6C), most pairs were well separated, and clustering occurred mainly by decay stage. Still, we found no evidence that INNER–OUTER dissimilarity changed systematically with decay (Fig. 6C), neither as progressive homogenization nor increasing divergence. NMDS segment lengths were stable across decay stages in both robust Aitchison and Jaccard space (Kruskal-Wallis p ≥ 0.14), with no monotonic trend (Spearman p ≥ 0.35) or decay-dependent changes in spread (Levene p ≥ 0.18). Together, these results reinforce that outer samples host higher richness, while inner samples represent a somewhat nested subset, especially when outer cores are particularly rich, albeit with substantial taxon turnover, with no consistent decay-related homogenization or divergence.

#### Scope 2B. SNAGs

Within snags, the most common SHs were *Eutypa spinosa* (SH1016964.10FU, 10.7%), *Fomes inzengae* (SH0762688.10FU, 6.2%) and *Kretzschmaria deusta* (SH0848643.10FU, 6.1%). Tree identity effects were only significant in presence–absence space (ΔR^2^_RA_ = 0.094, p = 0.16, ΔR^2^_JAC_ = 0.101, p < 0.001), and this pattern was corroborated by within versus between snag community similarity metrics. Within snags, substrate characteristics remained largely unresolved due to limited variation at the snag level and only two samples per snag. Still, cardinal direction showed a strong, stable effect in abundance-weighted space (R^2^≈0.12). The effect persisted after removal of taxa with the largest abundance shifts, indicating diffuse abundance shifts associated with cardinal direction. β-diversity was turnover-dominated with slightly higher T:N between snags than within snags (6.1 vs 5.5).

#### Scope 2C. Attached Fine Woody Debris (aFWD)

Within aFWD, the most common SHs were *Hypoxylon fragiforme* (SH1014579.10FU, 9.0%), *Mycetinis alliaceus* (SH0990859.10FU, 8.1%) and *H. macrocarpum* (SH0913330.10FU, 7.4%). Tree identity effects were non-significant in abundance-weighted space (ΔR^2^_RA_ = 0.086, p = 0.12) but remained weakly detectable in presence–absence space (ΔR^2^_JAC_ = 0.033, p = 0.004). Substrate effects were non-significant, except for diameter in the tree-blocked Jaccard model, but that effect was unstable in LOO. Within- versus between-tree contrasts confirmed higher within-tree similarity (AUC_RA_ = 0.65, AUC_JAC_ = 0.83). β-diversity was turnover-dominated, with higher T:N between deadwood units (8.0) than within (4.0). Nestedness within trees was comparatively high and similar to the inner–outer contrasts observed for logs in Scope 2A.

#### Scope 2D. Fallen Fine Woody Debris (fFWD)

Within fFWD, the most common SHs were *Hypoxylon macrocarpum* (SH0913330.10FU, 5.4%), *Jackrogersella cohaerens* (SH0896415.10FU, 4.9%) and *Mycetinis alliaceus* (SH0990859.10FU, 4.9%). A tree identity effect persisted only in presence–absence space (ΔR^2^_JAC_ = 0.040). Given that only three trees were sampled with equal replication, interpretation is based primarily on the unblocked PERMANOVA. In abundance-weighted space, substrate characteristics outweighed tree identity (R^2^ = 0.068). Decay stage was the dominant predictor (R^2^ = 0.148), with diameter contributing a sizeable effect (R^2^ = 0.051). In presence–absence space, tree identity and substrate characteristics were similar in magnitude (R^2^ ≈ 0.09), with decay stage and diameter each explaining ~0.04. Species richness showed a marginal increase at late decay (IRR = 1.25, p = 0.058), consistent with decay stage as the primary substrate driver. Within- versus between-tree contrasts showed little separation in abundance-weighted space (AUC_RA_ = 0.54) but a clearer signal in presence–absence space (AUC_JAC_ = 0.63). β-diversity was almost entirely turnover-driven, with similarly high T:N within (8.7) and between trees (9.9), comparable to cross-substrate contrasts. Nestedness contributions were negligible, indicating that individual fallen twigs host highly distinct assemblages. Overall, microhabitat variation and extreme piece-to-piece turnover dominated β-diversity patterns in fFWD, with only a minor tree identity imprint detectable in presence–absence space.

### Scope 3: Directed cross-substrate transitions

We tested three cross-substrate hypotheses reflecting physical continuity, detachment, and soil proximity among substrates.

#### Scope 3A. LOG–aFWD continuity

When considering logs and their aFWD as a continuous deadwood unit, both tree identity and substrate type had modest effects (tree: ΔR^2^_RA =_ 0.082, ΔR^2^_JAC_ = 0.064, substrate: R^2^_RA_ ≈ 0.09–0.1, R^2^_JAC_ = 0.027, all p = 0.001). Pairwise contrasts showed marginal evidence for trunk-branch continuity in abundance-weighted space (AUC_RA =_ 0.55, p = 0.078), but a within-tree signal in presence–absence space (AUC_JAC_ = 0.71, p < 0.001), although the median difference was small (Δmedian Jaccard = 0.055). LOG–aFWD differences were turnover-dominated (T:N_between_ = 8.3, T:N_within_ = 7.4), reinforcing that cross substrate contrasts are driven by taxon replacement.

#### Scope 3B. aFWD–fFWD effect of detachment

Detachment effects were small but consistent and expressed mainly in presence–absence space. In robust Aitchison space, both substrate type and tree identity were non-significant. In Jaccard space detachment was significant but had a small effect size (ΔR^2^_substrate_ ≈ 0.01) while tree identity remained more substantial (ΔR^2^_tree =_ 0.046). Pairwise contrasts mirrored this pattern with no separation in abundance space for tree identity and only marginal separation for substrate type versus clear within-substrate and within-tree similarity in Jaccard. β-diversity was near-completely turnover-driven (T:N_within_ = 8.5, T:N_between_ = 9.1). Thus, detachment explains ≤ 1% of variation and mainly alters which taxa are present while extreme piece to piece turnover is the dominant feature.

#### Scope 3C. Soil imprint on fFWD

Substrate identity was a strong predictor when contrasting fFWD with paired soils, explaining 36.4% of variation (ΔR^2^) in robust Aitchison space and 18.6% in Jaccard space. β-diversity between fFWD and soil was still turnover dominated, but T:N ratios were lower than for contrasts among woody substrates because of a larger nestedness component. Pair level imprint nulls (Suppl. material 1: fig. S6, Suppl. material 2: table S8) revealed only weak evidence for increased similarity. Under the global null four out of eleven pairs showed elevated similarity, 3 of which involved late decay fFWD. The strongest signal was in presence-absence, but even these retained high Jaccard dissimilarities of 0.87–0.98. Together, these results support strong compositional differences between soil and wood, with limited pair-specific convergence.

## Discussion

Fungal communities often exhibit signals linked to tree identity, but it remains unclear whether these patterns persist when environmental and host-species gradients are minimized. Our study system, a mono-dominant, unmanaged beech stand with limited environmental heterogeneity, reduces those gradients and allows us to test how substrate type and tree identity structure wood-inhabiting fungal (WIF) communities. To this end, we implemented a cross-fraction, tree-linked design connecting logs, snags, attached fine woody debris (aFWD), and fallen fine woody debris (fFWD) to their source tree.

### Scope 1: Stand-scale among-substrate structuring

Across substrates, richness continued to rise with sampling effort, whereas dominant diversity saturated rapidly (Fig. 2D, Suppl. material 1: fig. S4D). Common taxa were recovered consistently, while many rare SHs remained restricted to single samples or trees (Suppl. material 1: fig. S3), producing turnover-dominated differences among units. This heterogeneity is typical in deadwood metabarcoding studies (Kubartová et al. 2012; Baldrian et al. 2016) and frames the results below.

The three taxa with the highest contributions to β-diversity, *Armillaria ‘borealis’*, *Kretzschmaria deusta*, and *Eutypa spinosa*, are also some of the most common species recorded in fruitbody surveys of natural beech forests (Ódor et al. 2006). All are heartwood or butt-rotters of beech, often present before trees fall, and form interaction zone lines. *Armillaria* spp. can be pathogenic, increase moisture in occupied wood and cause white rot, whereas *K. deusta* and *E. spinosa* are soft-rot taxa that tolerate drier wood (Boddy 2021; Gilmartin et al. 2022). *E. spinosa* is often associated with early decay and sapwood colonization in attached branches and snags. These contrasts in niche position and decay mode are consistent with persistent within-tree heterogeneity and turnover-dominated structure among deadwood units.

Substrate-level species pools combined shared and substrate-associated taxa. A core group of 205 SHs (±10% of taxa) occurred across all woody substrates (Fig. 2A, Suppl. material 1: fig. S4A), yet substrate-specific SHs were more numerous. Between-substrate contrasts were strongly turnover-driven, with turnover-to-nestedness nearly twice as high between substrates as within substrates (Suppl. material 1: fig. S2), indicating replacement rather than one substrate representing a subset of another. Similarly, Fjelde et al. (2025) found that across 26 substrate types nearly half of fungal OTUs were substrate-specific, with deadwood contributing disproportionately to richness due to its high number of unique OTUs (>30%).

Substrate type explained more variance in abundance-weighted space (ΔR^2^_RA_ ≈ 0.08) than in presence–absence space (ΔR^2^_JAC_ ≈ 0.03). A comparable effect size was reported by Uhl et al. (2022) for contrasting CWD types (R^2^ = 0.11). Thus, differences among substrates were driven more by shifts in relative abundance than by changes in species membership. Although many taxa occur across substrates, substrate conditions modulate their relative abundances, likely reflecting shifts in competitive performance within shared species pools. Substrates therefore appear to reorganize dominance rather than forming independent assemblages. Because sporocarp formation requires sufficient resource capture, the more pronounced contrasts observed in fruitbody-based surveys among substrate types (Heilmann-Clausen and Christensen 2004; Uhl et al. 2022) may similarly reflect differences in dominance patterns.

Together, these findings show that deadwood substrates form distinct yet partially overlapping components of stand-level gamma diversity. Excluding any one substrate type would reduce that diversity and may disproportionately affect locally dominant fractions of the community.

Beyond substrate differentiation, we asked whether tree identity structures community composition at the stand scale. When all deadwood samples were analyzed jointly, tree identity explained a consistent fraction of compositional variance (ΔR^2^ ≈ 0.10). Given the high turnover characteristic of WIF, a null-corrected effect of this magnitude represents a non-trivial stand-scale signal. Deadwood from the same tree tended to be more similar in composition than deadwood from different trees, even across substrate types. In contrast to substrate type, whose effects were stronger in abundance-weighted space, tree identity effect sizes were comparable across distance metrics, indicating structuring of both species’ incidence and relative dominance. Ordination analysis (Fig. 4) visualized this diffuse but persistent clustering at the tree-level alongside substrate and decay gradients, a pattern also reported in other deadwood metabarcoding studies (Kubartová et al. 2012; Baldrian et al. 2016; Lepinay et al. 2021; Gilmartin et al. 2022; Korhonen et al. 2024). Such structuring therefore appears to be a general feature of WIF communities.

The persistence of tree identity across substrates further indicates that individual trees act as distinct community units. An alternative explanation for high turnover could be differences in propagule load within the stand. However, at this spatial scale (<480 m between samples), WIF are generally not considered dispersal-limited, as propagules are abundant and rare taxa show little spatial restriction at comparable distances (Rolstad et al. 2004; Abrego et al. 2018; Komonen and Müller 2018; Dickie et al. 2020). The tree-level signal is therefore unlikely to result from restricted dispersal alone. Instead, differences in colonization and establishment more likely explain the persistence of tree-specific community structure. In the absence of strong environmental gradients, those differences may arise from accumulated endophytic and bark-associated communities, subtle physiochemical variation among trees, and post-mortem assembly history shaped by priority effects (Fukami et al. 2010; Ottosson et al. 2014; Hiscox et al. 2016). Together, these results suggest that tree identity integrates pre-mortem biotic structure with subsequent assembly dynamics, allowing its imprint to persist as deadwood fragments and decays. The persistence of this signal across multiple deadwood fractions indicates that tree-level contingencies remain detectable despite physical fragmentation of the tree.

### Scope 2: Substrate-specific patterns within deadwood types

With stand-scale context established, we now focus on within-substrate processes: turnover, depth stratification, and the persistence of tree-level structure.

#### Scope 2A: Logs

In logs, outer wood was ~70% richer than inner wood, consistent with higher diversity in beech sapwood than heartwood (Leonhardt et al. 2019). Community differentiation among log samples was dominated by turnover. Even paired inner–outer samples showed turnover-dominated contrasts (T:N = 2.9), indicating that community similarity breaks down at centimeter scales. Therefore, logs behave as spatially compartmentalized substrates rather than homogeneous units, consistent with WIF occupying discrete habitat patches (Boddy 2021).

Despite this micro-scale heterogeneity, tree identity remained the strongest predictor of compositional variation (ΔR^2^ ≈ 0.09–0.17). Because no spatial autocorrelation was detected, this signal is unlikely to be a proximity artifact. Instead, it indicates that each log retains a distinct compositional signature, shaped by log-specific properties. Our results reinforce that tree identity should always be included as a random factor in WIF studies with multiple samples per deadwood unit, while it also represents meaningful ecological variation when interpreted explicitly.

Among measured substrate characteristics, decay stage explained the largest share of compositional variance (~3–9% depending on metric), although its effect remained modest compared to tree identity. Diameter, depth, and sampling position accounted for smaller fractions (<2%). Sampling position has likewise been shown to structure communities in conifers (Kubartová et al. 2012; Dahlberg et al. 2025; Naranjo-Orrico et al. 2025). Depth stratification also persisted across decay, as inner and outer communities remained compositionally distinct at all stages (Fig. 6). Outer wood acted as a more inclusive species pool, whereas inner assemblages were only partly nested within outer samples and remained turnover-dominated. In contrast to an outside-in migration scenario, we found no evidence for progressive homogenization with decay, nor for increasing dispersion that would indicate growing divergence between depth strata. Instead, inner and outer communities appear coupled but constrained from complete mixing. Thus, depth stratification appears to be a stable structural feature of beech logs, even under advancing decay.

Taken together, logs combine the strongest tree-identity imprint with pronounced within-log structuring, particularly along the depth axis.

#### Scope 2B: Snags

Snags showed the highest molecular richness, consistent with other metabarcoding studies of beech snags (Jomura et al. 2022; Uhl et al. 2022), but contrasting with fruitbody surveys where snags often appear species-poor, likely because low water availability limits sporocarp formation (Heilmann-Clausen and Christensen 2004; Pouska et al. 2016; Atrena et al. 2020). Uhl et al. (2022) attributed this discrepancy to elevated stress conditions in snags that relax competition and increase niche availability. Such conditions promote the coexistence of stress-tolerant taxa (Toljander et al. 2006) and inflate molecular richness, particularly of imperfect fungi, while simultaneously constraining or delaying fructification of sporocarp forming taxa. Consequently, snags host a broader suite of cryptic fungi and contribute disproportionately to the γ-diversity of the stand. Together, these findings highlight that beech snags make an outsized, but largely cryptic contribution to fungal diversity.

In our dataset, snag communities differed among trees primarily in species incidence rather than in abundance structure (ΔR^2^_JAC =_ 0.10, AUC_JAC_ = 0.87), indicating clear within–between separation. Within individual snags, substrate-level contrasts were weak, due to limited replication per tree. However, cardinal direction showed a consistent abundance-weighted signal. This effect was diffuse and not driven by a few dominant taxa, but reflected modest abundance shifts across multiple shared species. Although north–south microclimatic differences may underlie this pattern, such gradients were not directly measured and are likely subtle under a closed beech canopy.

Overall, snag communities combined high turnover among trees in presence–absence space with weak abundance structuring and modest, cardinal position modulation of abundances.

#### Scope 2C–D: fine woody debris (FWD)

Attached twigs and small branches represent exposed substrates with high surface-to-volume ratios and limited buffering, which should favor rapid colonizers tolerant of desiccation and disturbance (Boddy 2021). Consistent with this, aFWD was enriched in pyrenomycetous ascomycetes (e.g., *Hypoxylon* spp., *Jackrogersella cohaerens*), drought-tolerant endophytes also reported from beech FWD in surveys (Chapela and Boddy 1988; Nordén et al. 2004; Abrego and Salcedo 2013).

Although the total aFWD species pool was smaller than in other substrates, fruitbody studies have reported steeper richness increases per unit volume in FWD than in CWD (Heilmann-Clausen and Christensen 2004; Nordén et al. 2004). Accumulation curves here showed comparable richness increases in CWD and FWD. Because we sampled equal volumes, this suggests that the steeper per-volume patterns observed in fruitbody surveys may partly reflect a surface-area effect (Heilmann-Clausen and Christensen 2004) rather than intrinsically higher diversity in FWD.

The aFWD results illustrate how sample-level and community-level alpha diversity can diverge. At the sample-level, aFWD was as species rich as, or richer than, logs and fallen FWD, yet its total species pool was the smallest (Fig. 2C). Similar to snags, reduced buffering in small-diameter substrates may promote local co-existence while drawing from a more restricted pool of taxa capable of exploiting thin woody substrates. Tree identity only shaped incidence and was smaller (ΔR^2^_JAC_ = 0.03) compared to CWD fractions (ΔR^2^_JAC_ ≈ 0.09–0.1). The relatively high tree-level nestedness and elevated tree separation metrics further indicate that multiple attached twigs host overlapping subsets of a somewhat constrained, tree-associated species pool. Within aFWD, neither diameter nor decay stage had consistent effects further suggesting weaker substrate filtering at this scale.

Following detachment, tree identity remained equally weak in fFWD (ΔR^2^_JAC_ = 0.04), and, compared with aFWD, same-tree pairwise dissimilarities were even less separated from different-tree dissimilarities. In contrast, substrate characteristics dominated (decay class and size, ΣR^2^ ≈ 0.08–0.20), and turnover peaked (T:N ≥ 8.7). The high turnover among fFWD pieces is consistent with its fragmented nature, where many independent units allow colonization and competition dynamics to proceed largely independently (Korhonen et al. 2024). Similar rapid compositional change, short taxon residence times and strong microclimatic sensitivity have been reported in eDNA time series of beech and fir FWD (Brabcová et al. 2022). Although decay effects in deciduous fFWD have not always been detected (Korhonen et al. 2024), both size and decay contributed to differentiation in our dataset, suggesting increased substrate-level filtering.

Overall, metabarcoding and fruitbody research converge in identifying FWD as a highly dynamic, microclimate-sensitive habitat fraction characterized by diameter and decay filtering, rapid species replacement, and progressive erosion of tree-identity effects once branches become detached.

### Scope 3: Cross-substrate transitions

Scope 3 evaluates how fungal communities shift across substrate transitions accompanying tree fragmentation and decomposition. By contrasting coarse and fine woody fractions, attached and fallen FWD, and deadwood with soil, we mimic structural transitions that alter physical continuity, position, and environmental exposure.

Linking coarse and fine fractions, logs and their attached branches were more similar within the same tree than across trees, indicating modest trunk-branch continuity (ΔR^2^ ≈ 0.06–0.08). The signal was clearer in presence–absence space, whereas in abundance-weighted space it was weak (p = 0.03) and not supported by separation metrics. Thus, tree identity partly defines a shared species pool that extends across logs and their aFWD.

In contrast, substrate type explained more variation in abundances than in occurrences (R^2^_RA_ ≈ 0.09–0.10, R^2^_JAC_ = 0.027), indicating that the log–aFWD transition mainly reshapes which of those shared species become dominant. Similar trunk–branch differentiation has been reported in conifer systems (Korhonen et al. 2025), suggesting that this pattern is not restricted to beech and is consistent with diameter-related filtering across woody fractions. Several dominant log-associated taxa (e.g., *K. deusta*, *A. borealis*, *F. inzengae*) were prevalent in aFWD (Fig. 3) but remained low in relative abundance, with *E. spinosa* representing an exception known to colonize attached branches (Boddy 2021). Overall, while these taxa can colonize attached fine fractions, their dominance and fruiting require larger and more continuous resource volumes than provided by thin woody substrates.

Detachment itself explained little variance (~1%, only in presence–absence space), indicating that most replacement occurs among fine-wood pieces regardless of attachment state. Consequently, communities in fallen branches do not form coherent extensions or supersets of attached ones, but diverge in a piece-specific manner. Similar turnover-dominated β-diversity has been reported in other deadwood fungal systems (Ronold et al. 2026). Experimental contrasts between logs with soil-contact and artificially uplifted ones likewise show that soil contact and associated moisture shifts only modestly alter community composition (R^2^ ≈ 0.04 in Oechler et al. (2026)). In such highly dynamic substrates, pervasive turnover appears to limit the compositional signal attributable to detachment.

Soil and fFWD were strongly separated, yet their contrast included more nestedness than most deadwood-deadwood comparisons. This fits soil acting as a reservoir for some wood-associated taxa, while deadwood retains a substantial pool of exclusive fungi (Mäkipää et al. 2017). Pairwise imprint null models suggested only weakly elevated similarity for some late-decay pairs (Suppl. material 1: fig. S6), but replication was too low for robust conclusions. The absence of a clear pair-level signal may partly reflect bark removal prior to sampling, as bark is nutrient-rich (Mussche et al. 1998; André et al. 2010), supports high fungal diversity (Dierickx et al. 2024; Naranjo-Orrico et al. 2025), forms the direct interface with the surrounding forest floor, and likely represents a more readily colonized compartment than the wood itself.

Viewed across substrate types, community structure shifts from strongly tree-associated in coarse wood to increasingly piece-specific in smaller and detached fractions.

### Sampling and eDNA monitoring implications

Deadwood fungal communities are difficult to sample comprehensively using eDNA because sampled volumes are small and diversity is highly compartmentalized. Despite extensive, volume-standardized sampling, sample-level richness varied widely and total richness did not saturate. This pattern is consistent with ecological heterogeneity rather than methodological limitations and aligns with previous WIF metabarcoding studies (Kubartová et al. 2012; Runnel et al. 2021; Korhonen et al. 2024; Scali et al. 2025). Many taxa were restricted to individual samples, logs, or trees (Suppl. material 1: fig. S3), indicating that extensive replication is required to approximate total richness.

In contrast, dominant diversity (q_2_) saturated rapidly across substrates and required roughly 20–50 samples depending on substrate coverage (Fig. 2D, Suppl. material 1: fig. S4D). Additional replication yielded diminishing returns after common taxa were detected, both at the stand- and at the tree-level (Suppl. material 1: fig. S5). By comparison, typical diversity required substantially higher replication (±200 samples in this system). Sampling effort determines which diversity component is recovered: dominant diversity captures recurring structure, whereas rare taxa reflect localized microhabitats and stochastic variation. Long-read eDNA is effective for characterizing dominant assemblages and detecting cryptic fungi, but inefficient for exhaustive richness estimation under realistic monitoring effort (≤ 96 samples).

Spatial scale shapes inference. Within logs, communities differed even at centimeter scales. In contrast, no spatial autocorrelation was detected among logs, indicating that increasing distance among nearby deadwood units contributes little to stand-scale inference when environmental gradients are weak, reducing the need to sample far apart. Instead, compositional variation was primarily associated with tree identity, with deadwood units from the same tree showing greater similarity than expected from spatial proximity alone.

Sampling strategy at the micro-scale is therefore important. Wood anatomy shapes both retrieved community composition and richness and represents an additional and currently underappreciated axis of variation in WIF eDNA studies. Radial sampling across annual rings, sampling along growth rings from cut surfaces, or targeting specific wood compartments intercepts different anatomical microhabitats. Because fungal spread follows vessel and fiber networks, and radial connectivity of vessel networks is more restricted than longitudinal connectivity, drilling orientation and depth influence which mycelial territories are captured. Considering anatomical structure will therefore improve interpretation and comparability among studies.

These constraints inform sampling design. Pooling multiple cores per tree, including deeper wood fractions, can reduce micro-scale heterogeneity and stabilize compositional estimates, making tree-level replication more efficient than intensive within-log stratification for stand-level monitoring. Our data (Suppl. material 1: fig. S5) suggest that dominant diversity can be recovered with > 5 pooled samples per log. Capturing typical and total richness requires substantially more sampling and introduces additional challenges related to homogenizing larger volumes and subsampling for DNA extraction. Monitoring schemes should therefore prioritize pooling at the tree level for coarse woody debris, while maintaining broad substrate coverage across decay stages, diameter classes, and multiple trees. Substrates with lower richness, such as aFWD, may contain conservation-relevant taxa and should not be excluded (Uhl et al. 2022). By contrast, studies targeting ecological mechanisms should match sampling resolution to the spatial scale of the processes under investigation (Bosch et al. 2023).

From a practical perspective, intensive drilling-based eDNA sampling is not always necessary for monitoring dominant taxa. Fruitbody surveys remain cost-effective but are constrained by seasonality and detectability (Rieker et al. 2024). DNA-based approaches complement fruitbody observations by detecting taxa that rarely fruit or remain overlooked in field surveys.

Taken together, long-read eDNA reliably captures dominant and structurally recurrent components of deadwood fungal communities, whereas saturation of total richness remains unrealistic under a typical monitoring effort. Sampling strategies should therefore align with inferential goals: stand-scale monitoring benefits primarily from replication across trees and broad substrate coverage, whereas intensive stratification is most appropriate for hypothesis-driven studies.

## Figures and Tables

**Figure A1.**
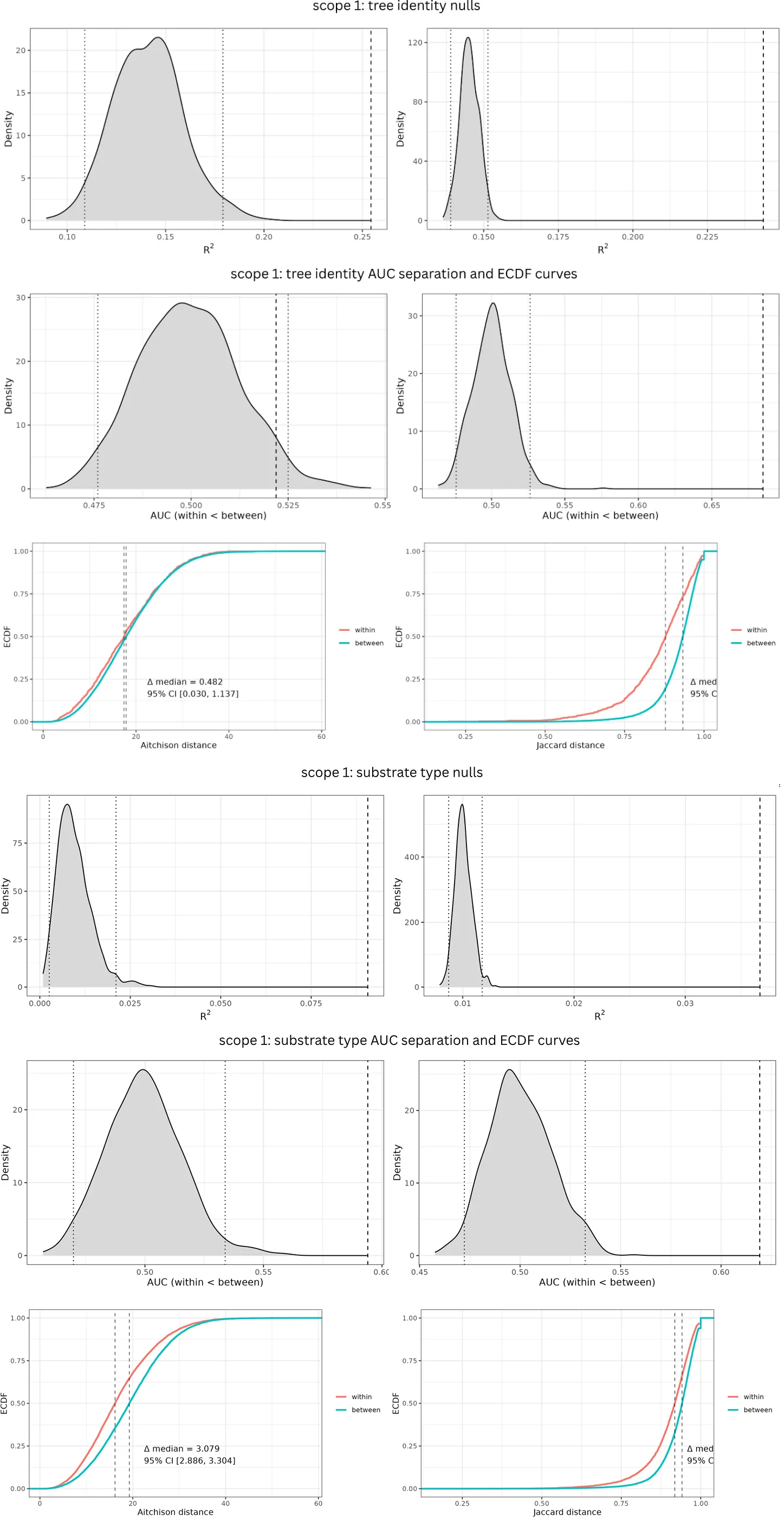
SCOPE 1 Cross substrates. Tree NULL and Substrate null.

**Figure A2.**
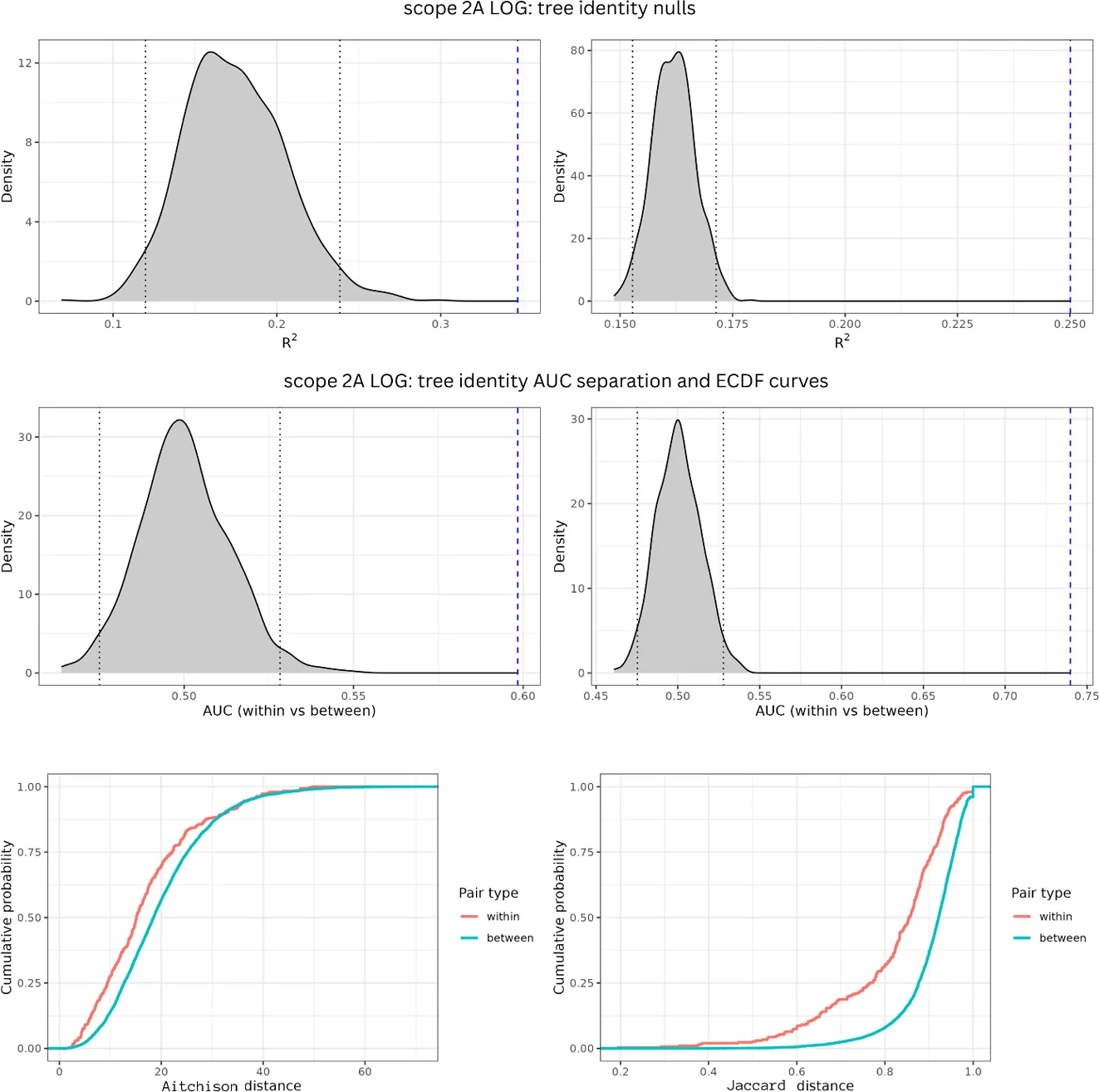
SCOPE 2A LOGs.

**Figure A3.**
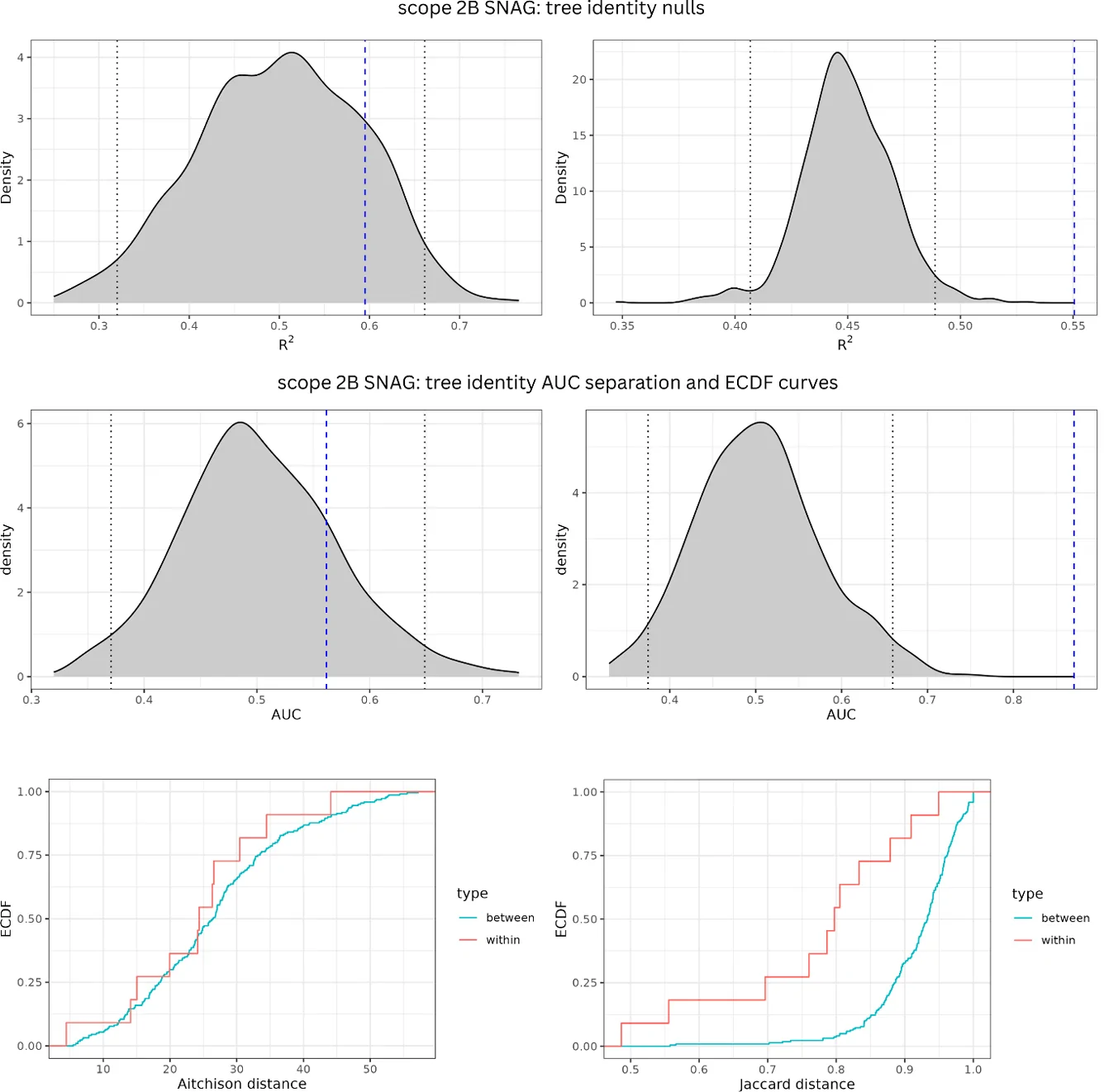
SCOPE 2B SNAGs.

**Figure A4.**
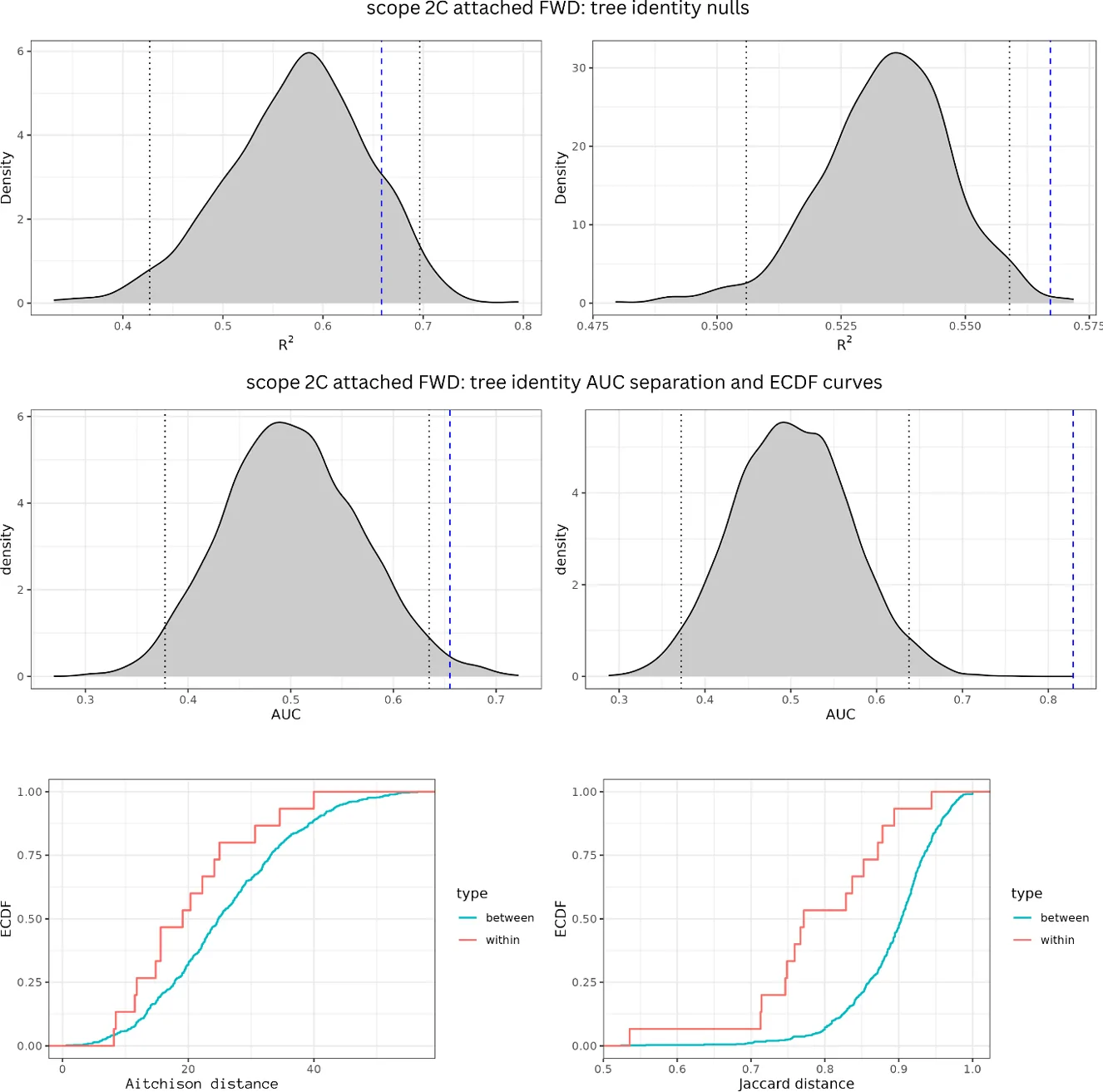
SCOPE 2C aFWD.

**Figure A5.**
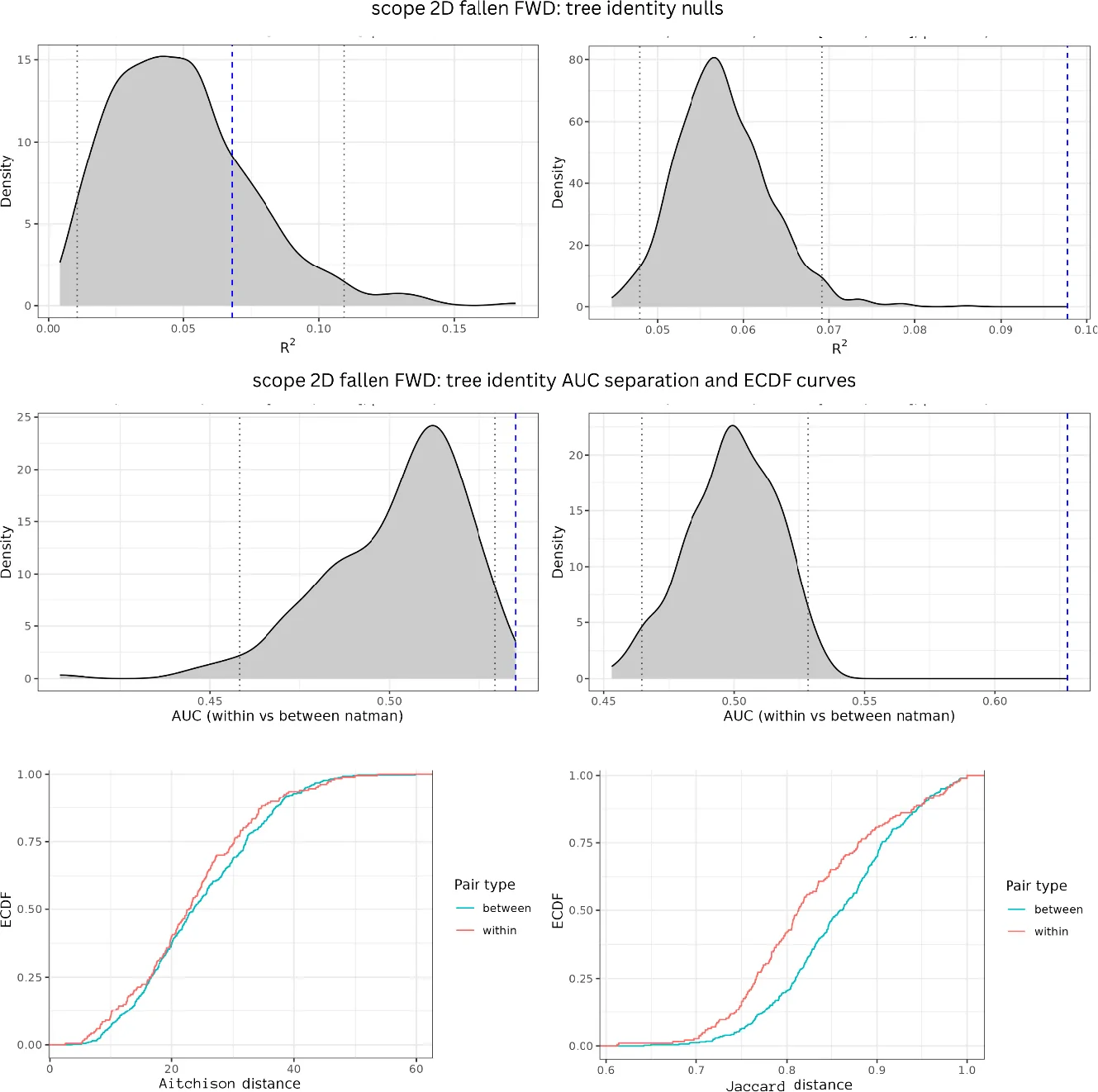
SCOPE 2D fFWD.

**Figure A6.**
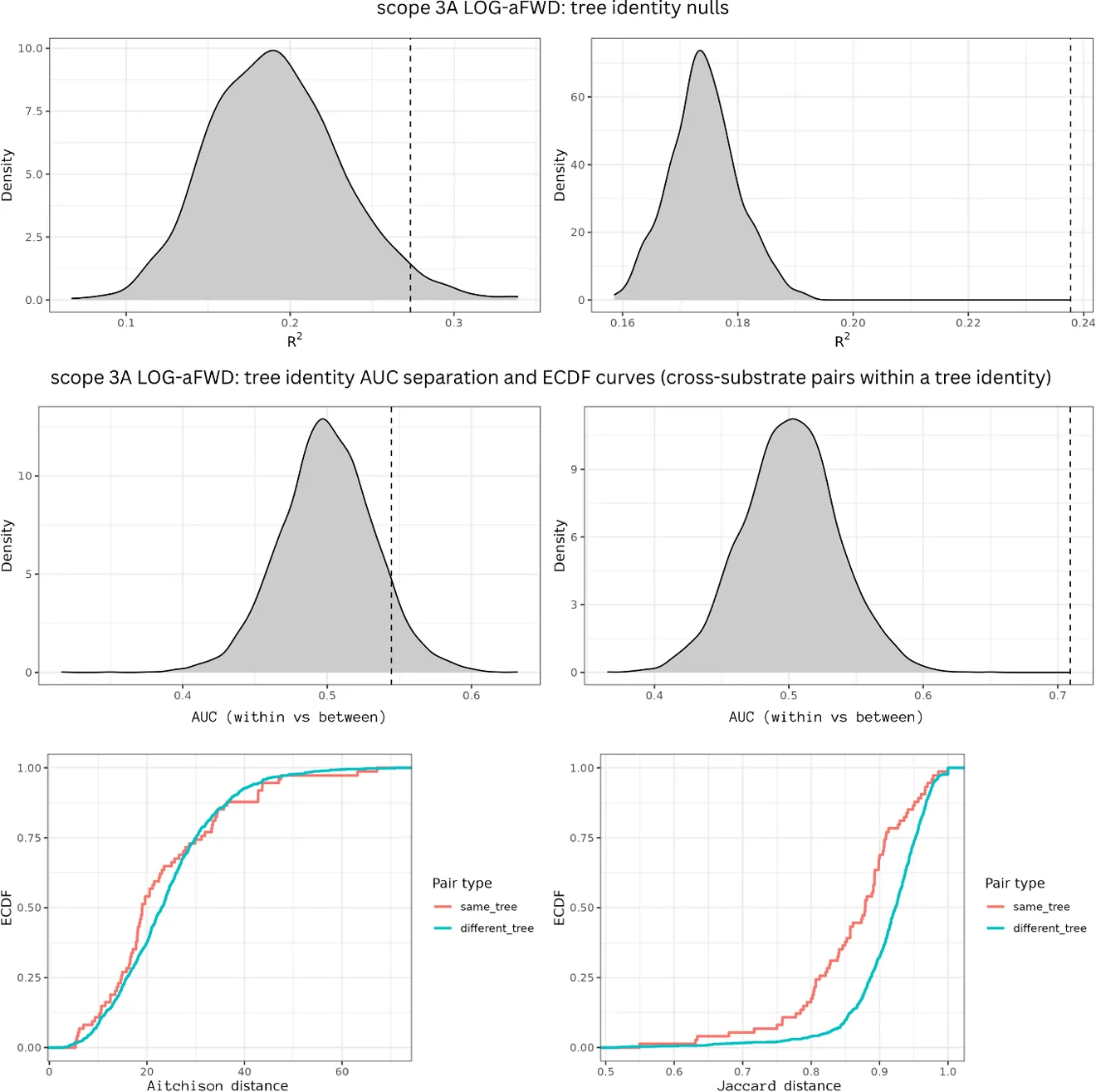
SCOPE 3A LOG-aFWD continuity.

**Figure A7.**
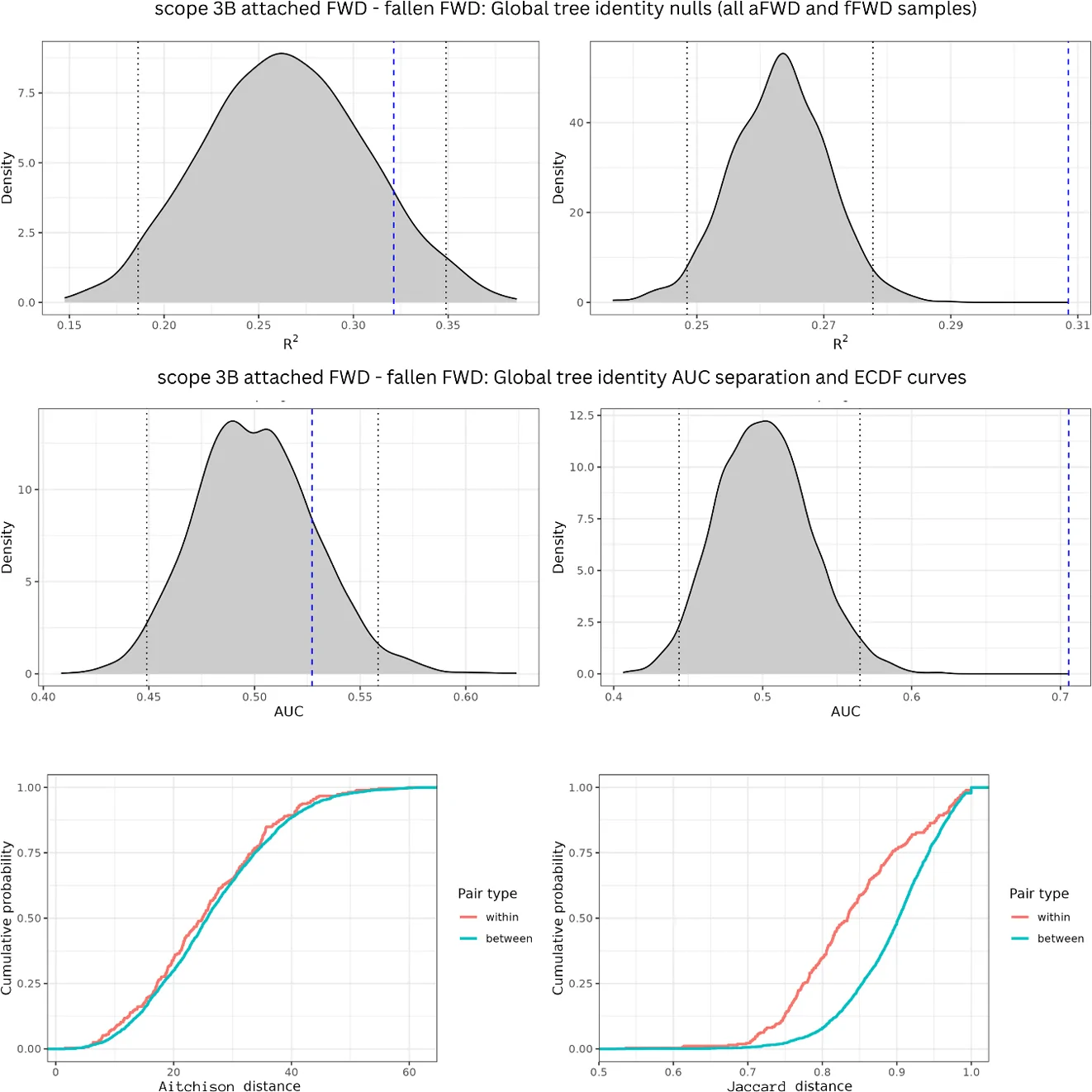
SCOPE 3B FWD detachment. Global NULL for tree identity.

**Figure A8.**
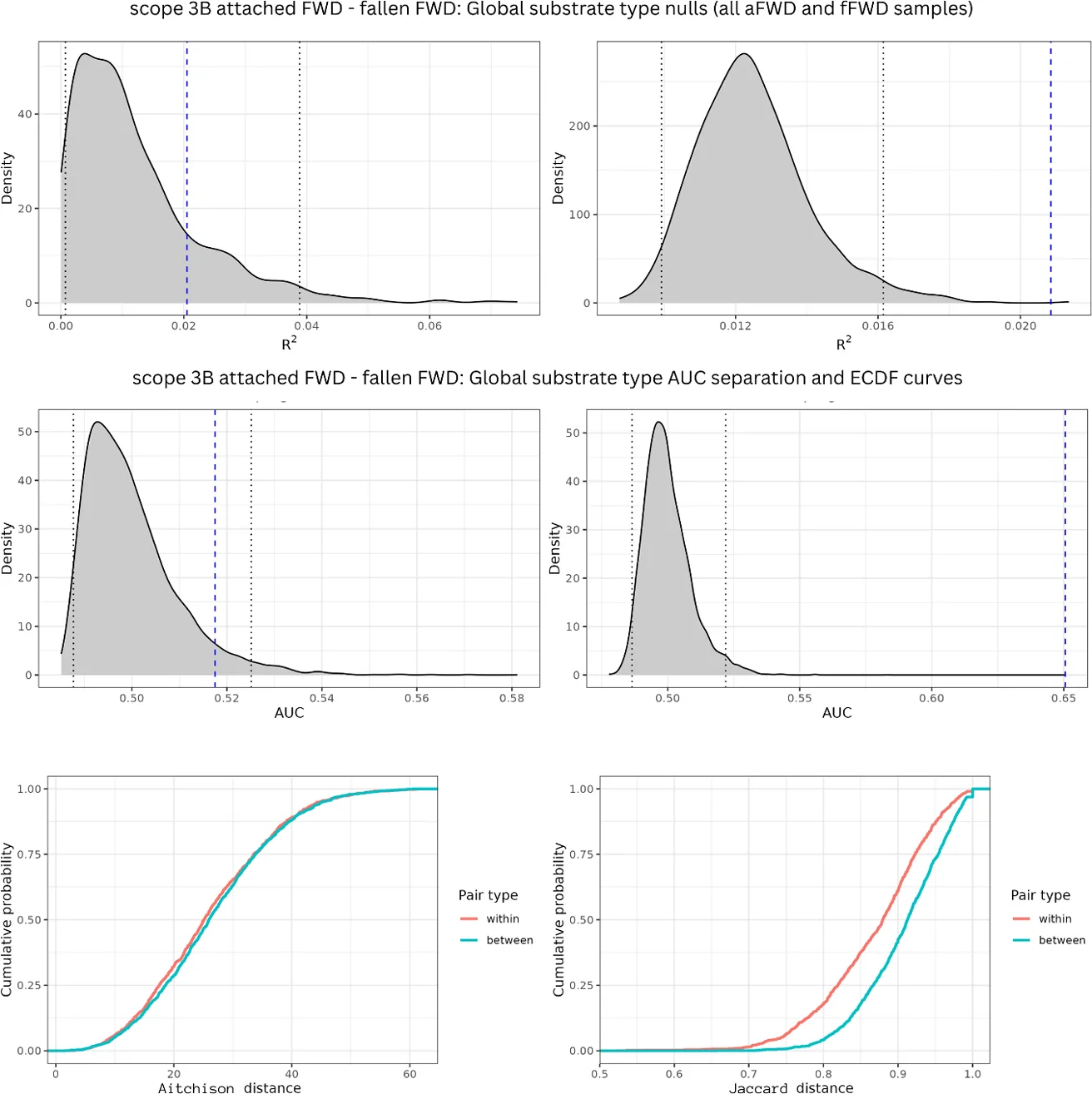
Global NULL for substrate.

**Figure A9.**
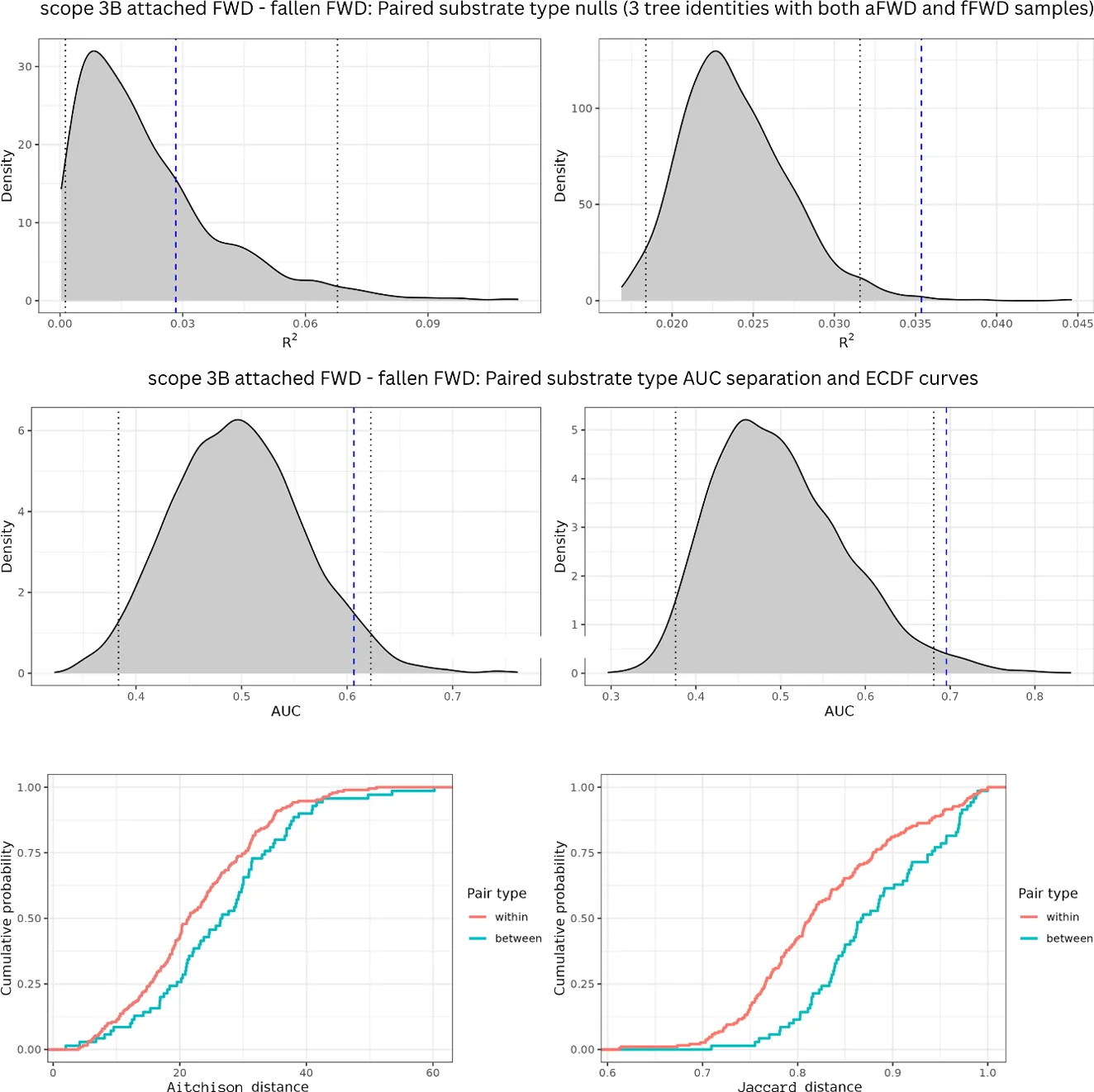
Paired NULL for substrate.

**Figure A10.**
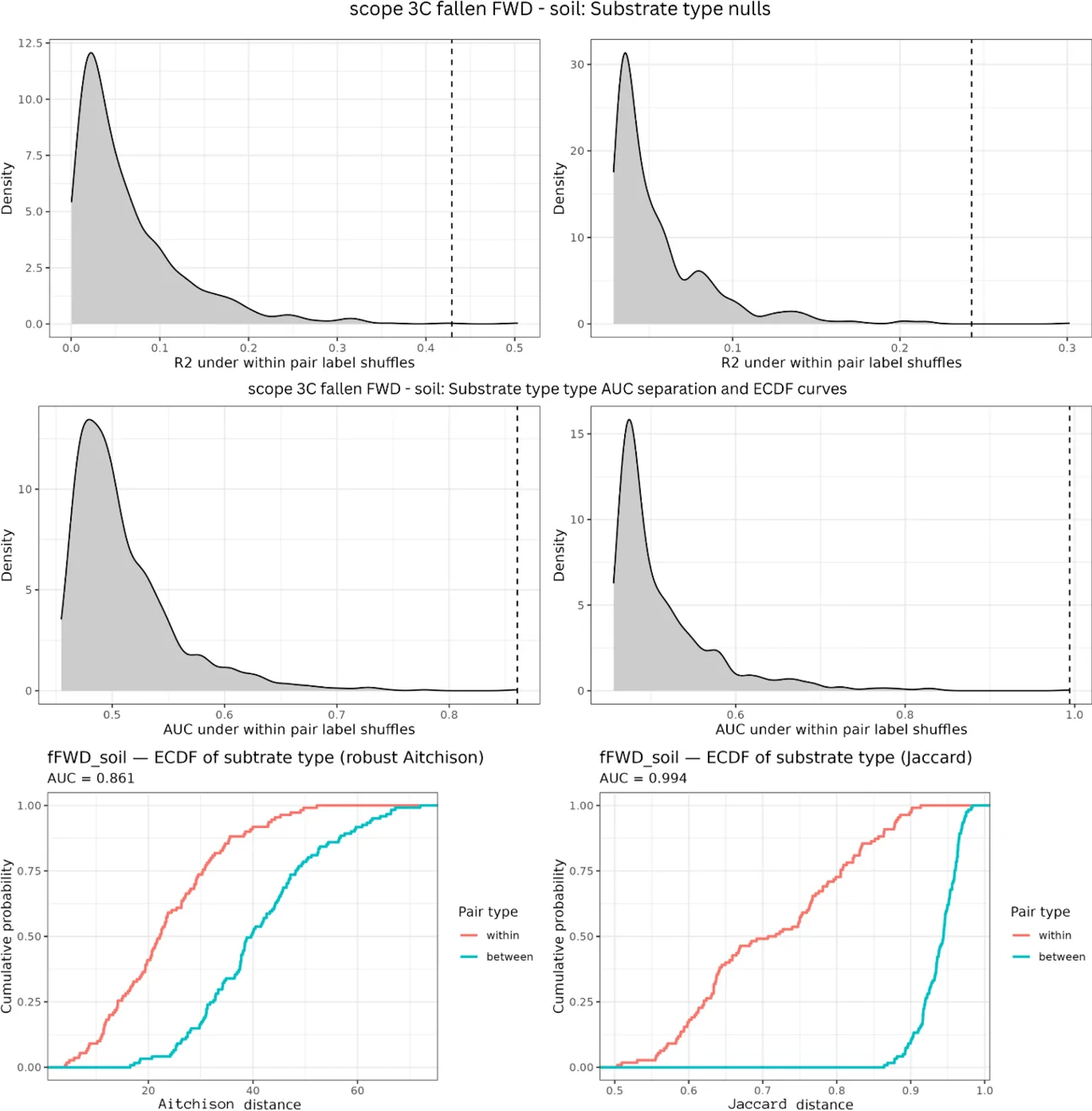
SCOPE 3C Soil imprint on fFWD.

## Appendix 1. Sampling protocol

All drilling used sterile technique. Before drilling we removed bark and loose debris with a sterile knife and a clean brush. Drill bits were changed between holes and decontaminated in sequence with a water rinse, 10% bleach, a second water rinse, 70% ethanol, and a brief flame with some time given to let the drill bit cool down. We sampled sawdust with a 10 mm × 150 mm wood auger mounted on a cordless MAKITA drill and drilled through a sterile 50 mL tube that was fashioned into a funnel (hole in screwcap and bottom cut off). A new funnel was used for each drill. For OUTER depth, we drilled an 8 cm deep core and collected shavings into sterile pre-labeled 50 mL tubes. We then repeated the procedure 5–10 cm further away and pooled the two yields to target maximally 16 cm^3^. For INNER depth we widened the first hole and deepened it by about 2 cm using a 45 mm spade bit. We then drilled a second 8 cm deep hole from the widened opening and collected shavings as the INNER sample. On logs this two-depth procedure was applied at BASE, MIDDLE, and UPPER positions, with two auger replicates per depth that were pooled as specified above. On attached and fallen fine woody debris, where diameters were small, we took a single pooled OUTER (8 cm deep or drilled through if diameter was less than 8 cm) auger sample per FWD piece. On snags we drilled at 1.37 m height on NORTH and SOUTH aspect positions with OUTER depth only, in duplicate as specified above. For soil we removed surface litter beneath 12 fallen fine woody debris pieces and used a 5 cm diameter soil auger to collect the organic horizon to about 5 cm depth. All tubes were kept on ice in the field and stored at -20 °C until DNA extraction. Equal sample volume was targeted across depths and substrates, and we noted any shortfalls for very small-diameter material.

## Appendix 2. Covariate level encoding

Diameter was standardized as a z-score and read counts (sequencing depth) were log transformed. All other predictors listed in Table 2 were treated as factors with the following levels: library (Table 1) had levels P2 and P3 for data generated in this study and MI and P1 for data generated in Dierickx et al. submitted (2026). Substrate had levels LOG, SNAG, aFWD, fFWD and SOIL. Decay stage had levels 1 to 5. Depth had levels INNER and OUTER. Position had levels BASE, MIDDLE and UPPER for log samples and NORTH and SOUTH for snag samples. For fFWD, decay class was encoded with levels EARLY and LATE. Early decay pieces retained bark, resisted breakage under mild force, and showed little to no penetration by a knife point. Late decay pieces had little to no bark remaining and were easily penetrated by a knife point. These categories broadly correspond to Renvall decay classes 1–2 (EARLY) and 4–5 (LATE), as applied in comparable wood-inhabiting fungi studies (Abrego and Salcedo 2011; Abrego and Salcedo 2013). For visualization in PCA only, Renvall decay stage 1 was encoded as EARLY decay, decay stages 2–3 as INTERMEDIATE and decay stages 4–5 as LATE decay classes.

## Appendix 3. Sensitivity Analyses

Sensitivity analyses were used to evaluate how robust the main community level results (unblocked and blocked PERMANOVA) were to influential trees, permutation design and the choice of summary statistic. Because the sampling design is hierarchical with strong tree level structure, often with few (highly variable) samples per tree identity, there is a risk that apparent effects of tree identity or microhabitat are driven by a few atypical trees rather than by consistent patterns. In addition, PERMANOVA effect sizes and p values can be influenced by dispersion differences among groups and by the chosen permutation scheme. The sensitivity framework therefore comprises a triple check: A) leave-one-tree-out (LOO) PERMANOVA (a form of jackknifing), B) within versus between group distance separation metrics, and C) design preserving null models that respect the sampling design.

**A) Leave-one-tree-out (LOO) PERMANOVA** addresses the impact of single tree identities on microhabitat effects sizes and significance. For each scope and distance metric, we refitted the blocked PERMANOVA after removing all samples belonging to a tree identity at a time and recorded the resulting R^2^ and p-values. This produces a distribution of effect sizes and p values over trees rather than a single point estimate. In the LOO summary table (Suppl. material 2: table S4) we report for each scope, model and predictor R^2^_median_ across LOO runs, together with the 95% confidence interval of permutation p-values. If the main results are robust, the R^2^_median_ will be similar to the R^2^_observed_ estimate and p-values will remain significant. Large differences between the R^2^_observed_ and the LOO R^2^_median_ or an increase in p-values when a tree identity is removed indicate that the corresponding effect is tree identity sensitive and should be interpreted with caution. This approach is particularly important in scopes with a modest number of trees, such as the SNAG (scope 2B) and FWD scopes (scope 2C-D, 3), where effect estimates could be driven by a small subset of (outlier) trees.

**B) Within versus between group separation metrics**

**Area Under the Curve (AUC)** captures if distances differ between levels of a grouping factor, in this case, either tree identity or substrate. We partitioned pairwise distances (both metrics) into within group and between group sets and quantified their separation using the AUC of the receiver operating characteristic associated with the Mann Whitney U test. AUC = 0.5 indicates that within and between distances are not distinguishable, AUC > 0.5 indicates that between group distances tend to be larger than within group distances, and AUC < 0.5 indicates the reverse. For example, AUC_observed_ = 0.73 means that in 73% of pairwise comparisons a between group distance exceeds a within group distance. This probability-based measure does not assume linear relationships and is less sensitive to differences in group size. To visualize the underlying distance patterns, we also plotted empirical cumulative distribution functions (ECDF) of within and between distances so that the magnitude and form of separation underlying the AUC values can be visually assessed.

**ANOSIM (Analysis of Similarity)** is conceptually related to AUC but uses a different summary statistic and permutation test. ANOSIM uses the ranks of the distance matrix and returns an R statistic that indicates how much between group ranks exceed within group ranks. R = 0 indicates no separation among groups, whereas R = 1 indicates complete separation. Because ANOSIM relies on ranks (PERMANOVA uses distances), it is affected differently by outliers and dispersion than PERMANOVA.

**C) Design preserving NULL models** were used to generate empirical expectations for PERMANOVA R^2^ and AUC under constrained randomization schemes that preserve group sizes and, where relevant, the distribution of samples over trees. For the tree identity effect, NULL distributions were obtained by shuffling tree labels across samples while keeping group sizes constant so that each permutation preserves the number of samples per tree identity. For substrate contrasts, substrate labels were shuffled among samples under the same constraint, or restricted within trees when the contrast is defined within tree legacies. NULL models produce reference distributions for how much variation explained or distance separation would be expected if tree or substrate identity did not matter beyond sample size structure, imposed by the study design.

We report in Suppl. material 2: table SS2, for each scope and metric the *R*^2^*_observed_*, the mean and 95%CI of the R^2^ values under the null model. From these values we derive the mean-corrected effect size ∆*R*^2^ = *R*^2^_*observed*_ – *mean* (*R*^2^*_null_*), the standardized effect size SES = ∆*R*^2^/*SD*(*R*^2^*_null_*) and the empirical p-value as the proportion of permutations where *R*^2^*_null_* ≥ *R*^2^_*observed*_. We focus on ∆*R*^2^ to assess effects beyond those expected from the design alone.

For the AUC based separation metrics we similarly report the AUC_observed_, mean and 95% CI AUC_NULL_, an empirical p-value and SES. For NULLs, 999 label shuffles were used. PERMANOVA used 999 permutations or 199 within a null permutation. ANOSIM used 9999 permutations.

The LOO analysis focuses on sensitivity to individual trees. The separation metrics AUC and ANOSIM quantify how strongly within and between group distances differ while ECDF plots visualize these differences. The NULL models evaluate whether R^2^_observed_ and AUC can be explained by the sampling design alone. Consistency across these complementary diagnostics increases confidence that the main conclusions about tree identity and microhabitat effects are not artefacts of a particular tree, metric or permutation scheme

Scope specific choices of grouping factors, permutation constraints and null model constructions are detailed in the individual scope descriptions.

## Appendix 4. Scope-specific descriptions

Per scope we detail which analyses were carried out in line with the main text in Materials and Methods section. We document any scope specific sample filtering, the parameter choices that deviate from the global settings and the rationale for these choices. Where relevant we outline subanalyses and grouping schemes used within a scope. For each analysis we also specify how the corresponding design preserving NULL model or models were constructed.

**Scope 1 across substrates**

For the across substrate scope we asked how fungal diversity and community composition differ among the four substrates at the plot scale, and how much of this structure is attributable to tree identity versus substrate identity. Starting from the global data set we removed soil samples. Tree identity (tree_identity) and library were treated as factors and a four level substrate factor with levels SNAG, aFWD, fFWD and LOG represented the deadwood type.

Alpha diversity across deadwood types was modelled with a GLMM with formula:

*richness* ~ *log_reads* + *library* + *dw_type* + (1 | *tree_identity*)

Deadwood type was coded with SNAG as the reference level for the fixed effect. Incidence rate ratios and their significance are reported in Suppl. material 2: table S6 and estimated marginal means for substrate in Suppl. material 2: table S7.

Community level analyses in the cross-substrate scope followed the general framework. Unblocked PERMANOVA to quantify tree identity and substrate effects with free permutations (by = ”terms”) was specified as

*distance_metric ~ log_reads + library + tree_identity + dw_type*

To isolate within tree differences among substrates we fitted a tree blocked PERMANOVA (by = ”margin”) with formula:

*distance_metric ~ log_reads + library + dw_type*

We applied LOO PERMANOVA to the tree blocked models (Suppl. material 2: table S4). Multivariate dispersion among substrates was evaluated with PERMDISP (Suppl. material 2: table S5).

We calculated within and between group distances for both tree identity and substrate type, deriving AUC and ANOSIM. We ran corresponding null models by refitting unblocked PERMANOVA, one for tree identity by permuting tree labels and one for substrate by shuffling substrate type labels. Simultaneously, we calculated AUC null metrics from the permuted data.

To deconstruct beta diversity into nestedness and turnover components, we applied betapart within and between substrate pairs.

We applied Hellinger transformation to the abundance otu-matrix as recommended for LCBD and SCBD. To identify influential samples and taxa in this cross scope setting we calculated local (LCBD) and species level (SCBD) contributions to β-diversity on the Hellinger transformed data. Using the beta.div function we obtained LCBD for each sample and SCBD for each SH. LCBD significance was evaluated with permutation tests and BH adjustment. No results are shown for LCBD as no samples were even marginally significant. SCBD is used to rank taxa by their contribution to overall β-diversity and is visualized in Fig. 3 for the 10 most contributing taxa. Occupancy frequency distributions (OFDs) were constructed by converting counts to presence–absence and calculating, for each SH, its occupancy k across sampling units. OFDs were computed at three scales: per sample, and after geometric collapse of counts per log, and per tree identity.

**Scope 2A LOG**

For this LOG scope, we elaborate more on each test. While we are briefer in other scopes, the same rationale applies.

Within this scope we ask which microhabitat predictors are important at the log scale. We also quantify the effect of the deadwood unit itself (tree identity) by comparing sample distances within and between logs. Samples were filtered for LOG substrate and restricted to focal logs by setting minimum sample number = 3 per tree (at least one per longitudinal position). The reason for restricting to focal logs was to have sufficient repetition per log to disentangle microhabitat factors.

Alpha richness was modelled with a GLMM (negative binomial) using log(reads) and library as technical covariates, and decay at drill point (ds_at_drill), depth class (depth_2, INNER vs OUTER), longitudinal position along the log (Position, BASE – MIDDLE - UPPER) and scaled diameter at drill (diameter_z) as microhabitat fixed effects. Tree identity was included as a random intercept (1 | tree_identity). Incidence rate ratios and their significance at level (Wald p) and factor (Type-II χ^2^ tests, anova chi^2^) are reported in Suppl. material 2: table S6, and estimated marginal means in Suppl. material 2: table S7.

We first fitted an unblocked PERMANOVA (free permutations, by = ”terms”) with the model

*distance_metric ~ log_reads + library + tree_identity + ds_at_drill + depth_2 + Position + diameter_at_drill_z*

to partition variation in a hierarchical manner among technical covariates, tree identity and microhabitat factors. To isolate within tree microhabitat effects we then fitted a tree identity blocked PERMANOVA (by = ”margin”) with the model

*distance_metric ~ log_reads + library + ds_at_drill + depth_2 + Position + diameter_at_drill_z*

and permutations stratified by tree identity (Suppl. material 2: table SS2). Dispersion differences among trees and among microhabitat factors were evaluated with PERMDISP (Suppl. material 2: table S5) as either

*anova(betadisper(distance_metric, predictor))*,

or, when a specific permutation scheme was used,

*permutest(betadisper(distance_metric, predictor), permutation_scheme)*.

To partition β-diversity into turnover and nestedness signals, we used betapart with the Sørensen distance and report median β_SIM_ (turnover component), β_SNE_ (nestedness component) and β_SOR_ (total) for within tree and between tree dissimilarities as well as the turnover:nestedness ratio (median β_SIM_ / median β_SNE_) for within and between tree groups (Suppl. material 2: table S9).

Sensitivity analysis was carried out as specified in Appendices 3, 4. LOO PERMANOVA was run on the blocked PERMANOVA model with results in Suppl. material 2: table S4 to assess stability of microhabitat terms to tree exclusion. NULL models were constructed by shuffling tree identity labels to grasp the effect of our study design on effect size and within versus between LOG pairwise differences were computed using AUC (with NULL) and ANOSIM (Suppl. material 2: table SS3) and visually confirmed with ECDF graphs (Appendix 5)

**SUB-SCOPE 2A LOG: Inner vs Outer samples**

Because depth was a significant microhabitat predictor and the samples were taken spatially adjacent, we implemented a dedicated INNER versus OUTER contrast to test whether INNER cores form subsets of OUTER cores at the same position on the same log. Starting from the global metadata we filtered to LOG samples and restricted the data to positions where we had data for both INNER and OUTER cores.

For each INNER–OUTER pair we computed pair level metrics describing overlap, subsetness and β-diversity. Pair-level metrics included richness, the number of shared and unique SHs, Sørensen dissimilarity, and the fractions of INNER species contained in OUTER and of OUTER species contained in INNER. Approximate subsetness was defined using a tolerance threshold of 2/3, that is at least two thirds of species in one sample present in the other. We also computed median β_SIM_, β_SNE_ and β_SOR_. In addition, Venn diagrams summarize unique and shared SH counts, and mean plus standard deviation of unique and shared SHs per pair (Fig. 6A) .

Subsetness patterns across all pairs were synthesized in a scatter plot of the fraction of INNER species contained in OUTER against the fraction of OUTER species contained in INNER (Fig. 6B). Reference iso-Sørensen curves were overlaid to highlight whether pairs are close to true nestedness or show more symmetric replacement.

Directionality of richness and subsetness was evaluated with non-parametric tests. First, a paired Wilcoxon test compared OUTER richness to INNER richness across all pairs to test whether outer cores are richer than inner cores. Second, one-sided Spearman correlations tested whether OUTER and INNER richness are respectively positively and negatively associated with the fraction of INNER species contained in OUTER, which would be expected if richer outer cores tend to accumulate the inner community and less diverse inner cores are sheltered from accumulating species.

We visualized INNER–OUTER sample pairs in NMDS ordinations (with k = 3 to reduce stress < 0.2), plotted pairs in the first two NMDS dimensions, and summarized within-pair ordination distances as boxplots by decay stage. Pair distances were computed as Euclidean segment lengths in the full three-dimensional NMDS configuration to assess whether decay impacts community dissimilarity. In this framework, decreasing segment lengths indicate progressive biotic homogenization between well connected depth strata, whereas increasing lengths indicate divergence consistent with strata acting as weakly connected habitats. Segment length differences among decay stages were tested with a Kruskal–Wallis test, trends with Spearman correlation, and dispersion differences with Levene tests.

**Scope 2B SNAG (standing deadwood)**

Goal: contrast positions and decay microhabitat factors and assess the importance of tree identity in SNAG substrate.

For the SNAG scope we first restricted the global data to substrate = SNAG and those snags that had two samples (one on position = south and one position = north).

Alpha diversity modelling in SNAGs used formula

*richness ~ log_reads + library + (1 | tree_identity) + ds_at_drill + position*

Incidence rate ratios and their significance are reported in Suppl. material 2: table S6, and estimated marginal means in Suppl. material 2: table S7.

Community level analyses in SNAGs followed the general PERMANOVA and sensitivity framework. For each metric we first ran an unblocked PERMANOVA (free permutations, by = ”terms”) with the model

*distance_metric ~ log_reads + library + tree_identity + ds_at_drill + position*

Tree identity blocked PERMANOVA used the same model without tree identity

*distance_metric ~ log_reads + library + ds_at_drill + position*

LOO PERMANOVA (tree identity blocked) results are in Suppl. material 2: table S4 and PERMDISP outcomes in Suppl. material 2: table S5. β-diversity was partitioned with betapart into median β_SIM_, β_SNE_ and β_SOR_ for within and between snag pairs and the associated turnover:nestedness ratio reported in Suppl. material 2: table S9. Within snag pairs were defined as opposite aspects of the same snag and between snag pairs as samples from different snags.

The paired north-south data set was used to compute AUC based separation of within versus between snags and to derive a design preserving null distribution for AUC that maintains the two samples per snag structure. In parallel we quantified tree identity structure with ANOSIM and evaluated the tree_identity R^2^_observed_ in unblocked PERMANOVA against a NULL distribution of R^2^ obtained by rerunning PERMANOVA with tree_identity replaced by random two sample groupings that mimic the paired snag design while keeping technical and snag level covariates unchanged.

**Scope 2C aFWD (attached fine woody debris)**

For the aFWD scope we restricted the global data set to dw_type = FWD and position = ATTACHED.

Alpha diversity was modelled with formula:

*richness ~ log_reads + library + (1 | tree_identity) + ds_at_drill + diameter_z*

Incidence rate ratios and their significance are reported in Suppl. material 2: table S6 and estimated marginal means for ds_at_drill in Suppl. material 2: table S7.

Community level analyses in aFWD followed the general framework. Unblocked PERMANOVA to quantify tree identity and local microhabitat effects with free permutations was specified as

*distance_metric ~ log_reads + library + tree_identity + ds_at_drill + diameter_at_drill_z*

To isolate within tree microhabitat effects we then fitted tree_identity blocked PERMANOVAs with permutations stratified by tree_identity:

*distance_metric ~ log_reads + library + ds_at_drill + diameter_at_drill_z*

PERMDISP outcomes are summarized in Suppl. material 2: table S5. β-diversity partitioning results are reported in Suppl. material 2: table S9.

NULL and sensitivity analyses followed the general framework. LOO PERMANOVA was applied to the tree_identity blocked models (Suppl. material 2: table S4). Design preserving NULL models for tree identity were constructed by shuffling tree labels across samples while preserving the observed number of aFWD samples per tree and rerunning unblocked PERMANOVAs with the shuffled group labels. Within versus between tree distance structure was quantified with AUC and ANOSIM using tree_identity as the grouping factor. Design preserving NULL distributions for AUC and ANOSIM were obtained with the same tree label shuffling scheme so that the observed samples per tree structure was maintained (Suppl. material 2: table SS3).

**Scope 2D fFWD (fallen fine woody debris)**

For the fFWD scope we restricted the global data to dw_type = FWD and position = FALLEN.

Alpha diversity modelling in fFWD used the formula

*richness ~ log_reads + (1 | tree_identity) + decay_stage + diameter_z*

Incidence rate ratios, significance tests and estimated marginal means for decay_stage are reported in Suppl. material 2: tables S6, S7.

For community composition we ran unblocked PERMANOVA with free permutations and the model:

*distance_metric ~ log_reads + tree_identity + decay_stage + diameter_at_drill_z*

and the blocked PERMANOVAs with permutations stratified by tree_identity:

*distance_metric ~ log_reads + decay_stage + diameter_at_drill_z*

Dispersion differences were evaluated with PERMDISP (Suppl. material 2: table S5). β-diversity partitioning using betapart is reported in Suppl. material 2: table S9.

NULL and sensitivity analyses followed the same design preserving framework as in other scopes targetting the tree legacy effect in fFWD. LOO PERMANOVA was applied to the tree_identity blocked models and is reported in Suppl. material 2: table S4. Design preserving tree_identity NULL models were constructed by shuffling tree labels across samples while preserving the observed number of samples per tree, rerunning unblocked PERMANOVAs and building NULL distributions for tree_identity R^2^. Within versus between tree distance structure was summarized with ECDF plots, AUC for discriminating within from between tree_identity distances, while ANOSIM provided the complementary rank-based R statistic. Design preserving NULL distributions for AUC were obtained with the same tree_identity shuffling scheme and all tree_identity evidence metrics are compiled in the summary table (Suppl. material 2: table SS3).

**Scope 3A LOG - aFWD continuity**

This scope tests whether fungal communities in aFWD are more similar to outer LOG samples from the same tree identity (continuous deadwood unit) than to LOG samples from other trees. In other words, we ask whether attached twigs and their supporting log form a shared tree specific community pool across a size gradient from coarse to fine deadwood. We did not fit a separate alpha richness model here because within substrate microhabitat effects were already evaluated for LOG and aFWD in scope 2. This scope therefore focuses on between substrate community composition and trunk-branch continuity.

For this scope we restricted the global data set to samples with dw_type equal to LOG or aFWD and retained only outer log cores at the three longitudinal positions BASE, MIDDLE and UPPER, together with aFWD positions AFWD1 and AFWD2. Depth filtering removed INNER log samples (aFWD samples are ‘outer’ samples and we did not want to introduce depth bias). We treated tree_identity and ds_at_drill as factors and included log_reads and library as technical nuisance terms.

To quantify tree identity and substrate effects at co-located LOG-aFWD scale, we first ran unblocked PERMANOVAs with free permutations (with by = “terms”) using the model:

*distance_metric ~ log_reads + library + tree_identity + dw_type*

These models partitioned variance hierarchically among technical covariates, tree identity and substrate type. To isolate within tree differences between LOG and aFWD we then fitted tree_identity blocked PERMANOVAs with permutations stratified by tree_identity (by = “margin”):

*distance_metric ~ log_reads + library + dw_type*.

The blocked models quantify how strongly LOG and aFWD differ within tree identities after adjusting for sequencing depth and library.

Dispersion differences were evaluated for tree_identity and substrate with PERMDISP (Suppl. material 2: table S5). Tree identity effects were further assessed with design preserving tree_identity null models based on the unblocked PERMANOVAs, analogous to earlier scopes. Sensitivity to individual trees was examined with LOO PERMANOVA on the tree_identity blocked models, summarized in Suppl. material 2: table S4.

To directly quantify trunk-branch continuity between LOG-aFWD samples we restricted all pair-based analyses to cross substrate pairs where one sample was LOG and the other aFWD, and classified each pair as within tree or between tree based on tree_identity. We then computed the AUC and oriented so that AUC > 0.5 indicates that LOG-aFWD pairs from the same tree tend to be more similar than LOG aFWD pairs drawn from different trees. Trunk-branch continuity patterns were visualized with ECDF graphs of these cross-type distances by pair class (Appendix 5). ANOSIM was run with the same pair-blocked groupings.

Trunk-branch continuity AUC values were evaluated against a design preserving tree_identity null model by permuting tree_identity labels and recomputing the LOG aFWDAUC for each permutation (Suppl. material 2: table SS3). To dissect how much of the LOG aFWD contrast is driven by species turnover versus nestedness we applied betapart to the same cross state LOG aFWD pairs and summarized partitions for within tree and between tree classes (Suppl. material 2: table S9).

**Scope 3B aFWD versus fFWD (detachment)**

For the aFWD versus fFWD scope we focused on detachment effects within FWD communities. The data set was restricted to samples with substrate equal to aFWD and fFWD. Because within substrate microhabitat effects such as decay stage and diameter were already analyzed in scopes 2C-D, this scope focuses on between state community composition and tree identity rather than refitting separate alpha richness models.

To tackle the unbalanced number of trees where FWD was sampled, we divided our analyses into two sets:

A global set where all aFWD samples (n = 34 across 20 trees) were included to contrast with fFWD samples (n = 35 across 3 trees). For this global set we tested both tree identity and substrate against a null model.

A paired set where only trees (n = 3) that have both aFWD (n = 6) and fFWD (35) samples were included. For the paired set we tested only substrate against a null model.

**Global unblocked comparison across all FWD**

For the global FWD set we used unblocked PERMANOVA (free permutations, by = ”terms”) to test for an overall detachment effect across all trees with model.

*distance_metric ~ log_reads + library + tree_identity +fwd_state*

To evaluate dispersion effects we applied PERMDISP with grouping by tree identity and by substrate (Suppl. material 2: table S5).

To characterize global separation between groups we grouped sample pairs by detachment state as either within substrate (aFWD-aFWD or fFWD-fFWD) or cross substrate (aFWD-fFWD). We then computed the AUC and oriented so that AUC > 0.5 indicates that cross substrate distances tend to exceed within substrate distances. The same procedure was applied with tree identity as a grouping factor to contrast within-tree and between-tree distances. ANOSIM was run in parallel with the same groupings (tree identity and substrate). In Suppl. material 2: table SS3, these are specified as the FWD GLOBAL substrate and tree NULL models.

Design preserving null models were constructed separately for tree identity and substrate. For tree identity we generated null distributions by shuffling tree labels while keeping the number of samples per tree constant and refitting the unblocked PERMANOVA model. The same permutations were used to derive null distributions for tree_identity AUC by recomputing within versus between tree AUC for each shuffle.

For detachment, we generated substrate null distributions by shuffling attached versus fallen labels across samples while preserving the total number of samples per substrate. For each permutation we refitted the unblocked PERMANOVA model and recomputed AUC for within versus cross substrate distances. These tree and substrate nulls define empirical expectations for R^2^ and AUC under random allocation of trees or detachment states that respects the observed sample size structure (Suppl. material 2: table SS3).

**Paired subset and tree blocked PERMANOVA**

The global model partly confounds detachment with tree identity as for many trees where aFWD was sampled, no fFWD was sampled. To isolate a within-tree detachment effect we constructed a paired subset consisting only of the 3 trees that contributed both attached and fallen FWD samples.

Within this paired subset we tested for detachment while controlling for tree identity by fitting tree blocked PERMANOVAs (by = ”terms”) with model

*distance_metric ~ log_reads + library + fwd_state*

To evaluate dispersion effects we applied PERMDISP with grouping by substrate (Suppl. material 2: table S5). To gauge turnover and nestedness we applied betapart to the cross substrate pairs and summarized partitions for within and between substrates classes (Suppl. material 2: table S9). Sensitivity to individual trees was examined with LOO PERMANOVA on the tree_identity blocked models, summarized in Suppl. material 2: table S4, but this analysis suffers power as only 2 trees are considered in each permutation with 3 total permutations.

Analogously to the global set, a Null model was constructed on the blocked PERMANOVA for substrate effect, but this time we restricted substrate label shuffles within each tree. From this permuted set, we also derived AUC NULL metrics (Suppl. material 2: table SS3, paired FWD substrate model).

**Scope 3C fFWD versus soil**

For the fFWD versus soil imprint scope we asked whether fallen fine woody debris carries a local soil signature. Specifically, we tested whether fungal assemblages in fallen twigs resemble those in the immediately underlying soil cores more than expected from random pairing while accounting for tree identity and pair structure.

From the global data set we selected fFWD samples that have an associated soil sample. This yielded a one-to-one set of fFWD SOIL pairs.

**Global paired type**

To quantify the overall compositional contrast between fFWD and soil while respecting the matched design, we fitted a PERMANOVA blocked by pair with model:

*distance_metric ~ log_reads + substrate*

Multivariate dispersion for substrate was evaluated with PERMDISP (Suppl. material 2: table S5). To gauge turnover and nestedness we applied betapart to the cross-substrate pairs and summarized partitions for within and between substrates classes (Suppl. material 2: table S9).

Evidence for a global fFWD versus soil effect was further summarized within the general sensitivity framework. We shuffled labels across substrates and refitted the pair-blocked PERMANOVA R^2^ and quantified within versus between substrate distances as AUC. ANOSIM with substrate as group was run in parallel (Suppl. material 2: table SS3).

**Pair level soil imprint tests**

Local soil imprint was evaluated at the scale of individual fFWD SOIL pairs. For each pair we calculated the observed Jaccard distance and the observed robust Aitchison distance between the two members of the pair. To obtain design preserving expectations we then generated two null models by randomly reassigning soil samples to fFWD samples. In a conservative tree blocked null, soil labels were permuted only among pairs belonging to the same tree identity so that each fFWD remained associated with a soil sample from the same tree but not necessarily its true partner. Because one tree contributed only three valid pairs due to failure of one soil sample in sequencing, the number of distinct permutations within that tree was small, which reduces the power of the blocked null. Therefore, we complemented this with a more liberal global null in which soil labels were randomly shuffled across all fFWD samples so that no fFWD retained its original soil partner. This global null approximates the expectation under arbitrary pairing of fFWD and soil samples across the plot while preserving the overall marginal distributions of soil and fFWD communities but is somewhat biased as local soils tend to be more alike and a tree identity effect exists (scope 2D).

For each null model and each pair we recomputed Jaccard similarity and Aitchison distance across permutations. For both distances we used left-tailed p-values (lower distance = more similar). p-values were computed separately for the tree blocked and global nulls and adjusted across pairs with the BH to get multiple-testing corrected q values. All statistics can be found in Suppl. material 2: table S8.

We also visualized the distributions for each pair in Suppl. material 1: fig. S6.

## Appendix 6. Consistency across amplification protocols

To test whether amplification protocol influenced inference, we repeated the Scope 1 and Scope 2A analyses separately for UMI-based and non-UMI libraries. Within each subset (UMI: MI and P1; non-UMI: P2 and P3; Table 1), we applied the same null-model framework for tree identity and substrate type and compared ΔR^2^, null distributions, AUC, and ECDF patterns with those from the full dataset.

Partitioning the data by amplification protocol did not alter the main conclusions. In both subsets, tree identity remained the dominant structuring factor across scopes (Suppl. material 1: fig. S7, Suppl. material 2: table S10), with observed effects clearly separated from their null expectations. Substrate effects were likewise detected in each dataset, but were more consistent under presence–absence distances. Differences emerged in abundance-weighted analyses. Substrate differentiation closely matched the full dataset in the non-UMI subset, where all major substrate categories were represented and abundance contrasts could be resolved. In the UMI subset, however, substrate effects were not significant in abundance-weighted space. This reflects the restricted substrate composition of the UMI data, which lacks fFWD and is dominated by log samples (Suppl. material 2: table S10). As a result, abundance-based contrasts among substrate types are inherently limited. The difference is therefore most parsimoniously explained by reduced substrate contrast and lower statistical power, rather than by an effect of the amplification protocol itself.

Overall, the ecological interpretation is unchanged. Tree identity consistently structures community composition, and substrate-related differentiation is recovered across amplification protocols. Protocol-specific null distributions and separation diagnostics are provided in Suppl. material 1: fig. S7 and Suppl. material 2: table S10.

## Additional information

### Artificial Intelligence (AI) use

Regarding the use of AI in the preparation of this manuscript, the authors declare the following:

AI was used to streamline the R scripts to better adherence to FAIR data principles. Figures generated in R were further refined using Canva, including integrated AI-assisted tools.

### Adherence to national and international regulations

All the fungal strains used in this study have been legally obtained, respecting the Convention on Biological Diversity (Rio Convention).

### Author ORCIDs

Glen Dierickx https://orcid.org/0000-0001-5387-8544

Kris Vandekerkhove https://orcid.org/0000-0003-1954-692X

Peter Van de Kerckhove https://orcid.org/0000-0002-8058-6798

Pieter Asselman https://orcid.org/0000-0003-3491-2174

Annemieke Verbeken https://orcid.org/0000-0002-6266-3091

### Data availability

All raw sequence data generated and analyzed in this study are deposited in the NCBI Sequence Read Archive under BioProject accession numbers PRJNA1328363 and PRJNA1428556. Species Hypothesis (SH) tables and associated sample metadata are provided as Supplementary Tables. Krona visualizations of taxonomic composition are available as downloadable supplementary files. All scripts used for data processing and statistical analyses are publicly available at GitHub: https://github.com/MycoMatics/scripts.

## Supplementary materials

Supplementary material 1 Additional figures Data type zip Explanation note Compressed (.zip) archive containing seven supplementary figures: **fig. S1**. Detailed drilling protocol and sampling scheme. **fig. S2**. Beta-diversity decomposition (turnover and nestedness components). **fig. S3**. Occupancy–frequency distributions across samples, logs, and tree identities. **fig. S4**. Substrate-level taxon share, rarefaction curves, and deadwood versus soil collector’s curves. **fig. S5**. Tree-level collector’s curves. **fig. S6**. fFWD–soil null model distributions. **fig. S7**. Robustness of null model results across amplification protocols (UMI vs non-UMI) https://binary.pensoft.net/file/1753432This dataset is made available under the Open Database License (http://opendatacommons.org/licenses/odbl/1.0/). The Open Database License (ODbL) is a license agreement intended to allow users to freely share, modify, and use this Dataset while maintaining this same freedom for others, provided that the original source and author(s) are credited. Glen Dierickx

Supplementary material 2 Additional tables Data type xlsx Explanation note This Excel workbook contains eleven sheets corresponding to the SH-table and tables S1–S10. Each sheet provides detailed results per analytical scope. SH_table_1_5: Species Hypothesis (1.5% threshold) × sample abundance matrix used in all analyses. **table SS1**. Sample-level metadata and corresponding SRA BioProject and BioSample accession numbers. **table SS2**. PERMANOVA results for unblocked and tree-blocked models across scopes and distance metrics, including model formulas and test statistics. **table SS3**. Design-preserving null model results, including ΔR^2^, standardized effect sizes, empirical p-values, and within-versus-between group separation metrics (AUC, ANOSIM). **table S4**. Leave-one-(tree)-out PERMANOVA results assessing robustness of tree identity. **table S5**. Multivariate dispersion (PERMDISP) results per scope and tested factor. **table S6**. Main alpha-diversity modelling results (negative-binomial GLMMs). **table S7**. Estimated marginal means and pairwise contrasts for alpha-diversity models. **table S8**. Null model results for paired fFWD–soil comparisons testing for elevated similarity. **table S9**. Median Sørensen-based beta-diversity decomposition (turnover and nestedness) and derived turnover:nestedness ratios. **table S10**. Subset analyses evaluating robustness of main results across amplification protocols (UMI vs non-UMI libraries). https://binary.pensoft.net/file/1753433This dataset is made available under the Open Database License (http://opendatacommons.org/licenses/odbl/1.0/). The Open Database License (ODbL) is a license agreement intended to allow users to freely share, modify, and use this Dataset while maintaining this same freedom for others, provided that the original source and author(s) are credited. Glen Dierickx

Supplementary material 3 Krona wheel charts Data type zip Explanation note Compressed (.zip) archive containing six Krona interactive charts in HTML format for browser viewing. The files display mean relative read proportions per taxon for: the full dataset (all woody samples combined), and each substrate separately: logs, snags, attached fine woody debris (aFWD), fallen fine woody debris (fFWD), and soil. Each Krona chart allows hierarchical exploration of taxonomic composition from higher ranks to Species Hypothesis level. https://binary.pensoft.net/file/1753434This dataset is made available under the Open Database License (http://opendatacommons.org/licenses/odbl/1.0/). The Open Database License (ODbL) is a license agreement intended to allow users to freely share, modify, and use this Dataset while maintaining this same freedom for others, provided that the original source and author(s) are credited. Glen Dierickx

## References

[B1] Abarenkov K, Kõljalg U, Nilsson RH (2022) UNITE Species Hypotheses Matching Analysis. In: Biodiversity Information Science and Standards. Pensoft Publishers 6: e93856. 10.3897/biss.6.93856

[B2] Abarenkov K, Nilsson RH, Larsson K-H et al. (2024) The UNITE database for molecular identification and taxonomic communication of fungi and other eukaryotes: sequences, taxa and classifications reconsidered. Nucleic Acids Research 52: D791–D797. 10.1093/nar/gkad1039 PMC10767974 37953409

[B3] Abrego N, Salcedo I (2011) How does fungal diversity change based on woody debris type? A case study in Northern Spain. Ekologija 57(3): 109–119. 10.6001/ekologija.v57i3.1916

[B4] Abrego N, Salcedo I (2013) Variety of woody debris as the factor influencing wood-inhabiting fungal richness and assemblages: Is it a question of quantity or quality? Forest Ecology and Management 291: 377–385. 10.1016/j.foreco.2012.11.025

[B5] Abrego N, Norros V, Halme P et al. (2018) Give me a sample of air and I will tell which species are found from your region: Molecular identification of fungi from airborne spore samples. Molecular Ecology Resources 18: 511–524. 10.1111/1755-0998.12755 29330936

[B6] André F, Jonard M, Ponette Q (2010) Biomass and nutrient content of sessile oak (*Quercus petraea* (Matt.) Liebl.) and beech (*Fagus sylvatica* L.) stem and branches in a mixed stand in southern Belgium. Science of The Total Environment 408: 2285–2294. 10.1016/j.scitotenv.2010.02.040 20231032

[B7] Atrena A, Banelytė GG, Læssøe T et al. (2020) Quality of substrate and forest structure determine macrofungal richness along a gradient of management intensity in beech forests. Forest Ecology and Management 478: 118512. 10.1016/j.foreco.2020.118512

[B8] Baldrian P, Zrůstová P, Tláskal V et al. (2016) Fungi associated with decomposing deadwood in a natural beech-dominated forest. Fungal Ecology 23: 109–122. 10.1016/j.funeco.2016.07.001

[B9] Baselga A, Orme D, Villeger S et al. (2025) betapart: Partitioning Beta Diversity into Turnover and Nestedness Components. https://CRAN.R-project.org/package=betapart

[B10] Bates D, Maechler M, Bolker B et al. (2015) Fitting Linear Mixed-Effects Models Using lme4. Journal of Statistical Software 67: 1–48. 10.18637/jss.v067.i01

[B11] Boddy L (2001) Fungal community ecology and wood decomposition processes in angiosperms: from standing tree to complete decay of coarse woody debris. Ecological Bulletins 49: 43–56. https://www.jstor.org/stable/20113263

[B12] Boddy L (2021) Fungi and trees: Their complex relationships. Arboricultural Association, Gloucestershire, 306 pp.

[B13] Bosch J, Némethová E, Tláskal V et al. (2023) Bacterial, but not fungal, communities show spatial heterogeneity in European beech (*Fagus sylvatica* L.) deadwood. FEMS Microbiology Ecology 99: fiad023. 10.1093/femsec/fiad023 PMC10065134 36906283

[B14] Brabcová V, Tláskal V, Lepinay C et al. (2022) Fungal Community Development in Decomposing Fine Deadwood Is Largely Affected by Microclimate. Frontiers in Microbiology 13. 10.3389/fmicb.2022.835274 PMC9045801 35495708

[B15] Burrascano S, Trentanovi G, Paillet Y et al. (2021) Handbook of field sampling for multi-taxon biodiversity studies in European forests. Ecological Indicators 132: 108266. 10.1016/j.ecolind.2021.108266

[B16] Chao A, Gotelli NJ, Hsieh TC et al. (2014) Rarefaction and Extrapolation with Hill Numbers: A Framework for Sampling and Estimation in Species Diversity Studies. Ecological Monographs 84: 45–67. 10.1890/13-0133.1

[B17] Chapela IH, Boddy L (1988) Fungal colonization of attached beech branches. New Phytologist 110: 39–45. 10.1111/j.1469-8137.1988.tb00235.x

[B18] Christensen M, Emborg J, Hahn K et al. (2005) Wood-inhabiting fungi as indicators of nature value in European beech forests. Monitoring and Indicators of Forest Biodiversity in Europe from Ideas to Operationality. EFI Proceedings 51: 229–237.

[B19] Dahlberg A, Pioli S, Jönsson M et al. (2025) Detailed analysis of fungal communities in Norway spruce logs reveals stochastic fine-scale patterns of species and detects lichen forming fungi without their photobionts. Fungal Ecology 78: 101458. 10.1016/j.funeco.2025.101458

[B20] De Keersmaeker L, Esprit M, Goessens S et al. (2023) Monitoring programme on strict forest reserves in Flanders (Belgium) - site level stand structure, regeneration and vegetation data. 10.5281/zenodo.8017674

[B21] Dickie IA, Wakelin A, Richardson SJ (2020) Rare species of wood-inhabiting fungi are not local. Ecological Applications 30: e02156. 10.1002/eap.2156 32358821

[B22] Dierickx G, Tondeleir L, Asselman P et al. (2024) What Quality Suffices for Nanopore Metabarcoding? Reconsidering Methodology and Ectomycorrhizae in Decaying *Fagus sylvatica* Bark as Case Study. Journal of Fungi 10: 708. 10.3390/jof10100708 PMC11508852 39452660

[B23] Dierickx G, Asselman P, Van Nieuwerburgh F et al. (2026) Do we need Unique Molecular Identifiers in nanopore metabarcoding?

[B24] Dyson BL, Brabcová V, Baldrian P et al. (2026) Experimentally manipulating forest structure to mimic management strategies: effects on deadwood fungal diversity and decomposition. FEMS Microbiology Ecology: fiag011. 10.1093/femsec/fiag011 PMC12927422 41671191

[B25] Fjelde M, Kauserud H, Halvorsen R et al. (2025) Ecosystem sequencing of the boreal forest mycobiome reveals high substrate specificity. 10.21203/rs.3.rs-7774568/v1 42247207

[B26] Fukami T, Dickie IA, Paula Wilkie J et al. (2010) Assembly history dictates ecosystem functioning: evidence from wood decomposer communities: Carbon dynamics and fungal community assembly. Ecology Letters 13: 675–684. 10.1111/j.1461-0248.2010.01465.x 20412280

[B27] Furneaux B, Bahram M, Rosling A et al. (2021) Long‐ and short‐read metabarcoding technologies reveal similar spatiotemporal structures in fungal communities. Molecular Ecology Resources 21: 1833–1849. 10.1111/1755-0998.13387 33811446

[B28] Gardes M, Bruns TD (1993) ITS primers with enhanced specificity for Basidiomycetes - application to the identification of mycorrhizae and rusts. Molecular Ecology 2: 113–118. 10.1111/j.1365-294X.1993.tb00005.x 8180733

[B29] Gilmartin EC, Jusino MA, Pyne EJ et al. (2022) Fungal endophytes and origins of decay in beech (*Fagus sylvatica*) sapwood. Fungal Ecology 59: 101161. 10.1016/j.funeco.2022.101161

[B30] Halme P, Holec J, Heilmann-Clausen J (2017) The history and future of fungi as biodiversity surrogates in forests. Fungal Ecology 27: 193–201. 10.1016/j.funeco.2016.10.005

[B31] Heilmann-Clausen J, Christensen M (2004) Does size matter?: On the importance of various dead wood fractions for fungal diversity in Danish beech forests. Forest Ecology and Management 201: 105–117. 10.1016/j.foreco.2004.07.010

[B32] Heilmann-Clausen J, Aude E, van Dort K et al. (2014) Communities of wood-inhabiting bryophytes and fungi on dead beech logs in Europe – reflecting substrate quality or shaped by climate and forest conditions? Journal of Biogeography 41: 2269–2282. 10.1111/jbi.12388

[B33] Hiscox J, O’Leary J, Boddy L (2018) Fungus wars: basidiomycete battles in wood decay. Studies in Mycology 89: 117–124. 10.1016/j.simyco.2018.02.003 PMC6002336 29910518

[B34] Hiscox J, Savoury M, Johnston SR et al. (2016) Location, location, location: priority effects in wood decay communities may vary between sites. Environmental Microbiology 18: 1954–1969. 10.1111/1462-2920.13141 26626102

[B35] Hsieh TC, Ma KH, Chao A (2025) iNEXT: iNterpolation and EXTrapolation for Species Diversity. Available from: http://chao.stat.nthu.edu.tw/wordpress/software-download/

[B36] Jomura M, Yoshida R, Michalčíková L et al. (2022) Factors Controlling Dead Wood Decomposition in an Old-Growth Temperate Forest in Central Europe. Journal of Fungi 8: 673. 10.3390/jof8070673 PMC9325057 35887430

[B37] Juutilainen K, Halme P, Kotiranta H et al. (2011) Size matters in studies of dead wood and wood-inhabiting fungi. Fungal Ecology 4: 342–349. 10.1016/j.funeco.2011.05.004

[B38] Kahl T, Arnstadt T, Baber K et al. (2017) Wood decay rates of 13 temperate tree species in relation to wood properties, enzyme activities and organismic diversities. Forest Ecology and Management 391: 86–95. 10.1016/j.foreco.2017.02.012

[B39] Komonen A, Müller J (2018) Dispersal ecology of deadwood organisms and connectivity conservation. Conservation Biology 32: 535–545. 10.1111/cobi.13087 29388249

[B40] Korhonen A, Siitonen J, Hamberg L (2024) Fungal and beetle diversity in deciduous fine woody debris in spruce-dominated forests in relation to substrate quantity and quality. Biodiversity and Conservation 33: 4121–4137. 10.1007/s10531-024-02942-6

[B41] Korhonen A, Siitonen J, Hamberg L (2025) How do attached crown parts and branches contribute to the diversity of saproxylic fungi and beetles in downed and decaying spruce trees? Biodiversity and Conservation 34: 481–494. 10.1007/s10531-024-02982-y

[B42] Kubartová A, Ottosson E, Dahlberg A et al. (2012) Patterns of fungal communities among and within decaying logs, revealed by 454 sequencing. Molecular Ecology 21: 4514–4532. 10.1111/j.1365-294X.2012.05723.x 22882383

[B43] Küffer N, Gillet F, Senn-Irlet B et al. (2008) Ecological determinants of fungal diversity on dead wood in European forests. Fungal Diversity 30: 83–95. https://hal.science/hal-00357745v1

[B44] Lenth RV, Piaskowski J (2025) emmeans: Estimated Marginal Means, aka Least-Squares Means. https://CRAN.R-project.org/package=emmeans

[B45] Leonhardt S, Hoppe B, Stengel E et al. (2019) Molecular fungal community and its decomposition activity in sapwood and heartwood of 13 temperate European tree species. PLoS ONE 14: e0212120. 10.1371/journal.pone.0212120 PMC6375594 30763365

[B46] Lepinay C, Jiráska L, Tláskal V et al. (2021) Successional Development of Fungal Communities Associated with Decomposing Deadwood in a Natural Mixed Temperate Forest. Journal of Fungi 7: 412. 10.3390/jof7060412 PMC8228407 34070657

[B47] Mäkipää R, Rajala T, Schigel D et al. (2017) Interactions between soil- and dead wood-inhabiting fungal communities during the decay of Norway spruce logs. The ISME Journal 11: 1964–1974. 10.1038/ismej.2017.57 PMC5563949 28430188

[B48] Mussche S, Bussche B, De Schrijver A et al. (1998) Nutrient uptake of a mixed oak/beech forest in Flanders (Belgium). Silva Gandavensis 63. 10.21825/sg.v63i0.840

[B49] Naranjo-Orrico D, Purhonen J, Furneaux B et al. (2025) The Importance of Within-Log Sampling Replication in Bark- and Wood-Inhabiting Fungal Metabarcoding Studies. Environmental DNA 7: e70181. 10.1002/edn3.70181

[B50] Niskanen T, Lücking R, Dahlberg A et al. (2023) Pushing the Frontiers of Biodiversity Research: Unveiling the Global Diversity, Distribution, and Conservation of Fungi. Annual Review of Environment and Resources 48: 149–176. 10.1146/annurev-environ-112621-090937

[B51] Nordén B, Ryberg M, Götmark F et al. (2004) Relative importance of coarse and fine woody debris for the diversity of wood-inhabiting fungi in temperate broadleaf forests. Biological Conservation 117: 1–10. 10.1016/S0006-3207(03)00235-0

[B52] Ódor P, Heilmann-Clausen J, Christensen M et al. (2006) Diversity of dead wood inhabiting fungi and bryophytes in semi-natural beech forests in Europe. Biological Conservation 131: 58–71. 10.1016/j.biocon.2006.02.004

[B53] Odriozola I, Abrego N, Tláskal V et al. (2021) Fungal Communities Are Important Determinants of Bacterial Community Composition in Deadwood. Shade A (Ed.). mSystems 6: e01017-20. 10.1128/mSystems.01017-20 PMC7786133 33402349

[B54] Oechler H, Krah F-S, Schreiber J et al. (2026) Disentangling effects of structural deadwood characteristics on fungal and bacterial diversity and assembly processes. Forest Ecology and Management 599: 123268. 10.1016/j.foreco.2025.123268

[B55] Oksanen J, Simpson GL, Blanchet FG et al. (2025) vegan: Community Ecology Package. https://CRAN.R-project.org/package=vegan

[B56] Ottosson E, Nordén J, Dahlberg A et al. (2014) Species associations during the succession of wood-inhabiting fungal communities. Fungal Ecology 11: 17–28. 10.1016/j.funeco.2014.03.003

[B57] Ovaskainen O, Schigel D, Ali-Kovero H et al. (2013) Combining high-throughput sequencing with fruit body surveys reveals contrasting life-history strategies in fungi. The ISME Journal 7: 1696–1709. 10.1038/ismej.2013.61 PMC3749500 23575372

[B58] Oxford Nanopore Technologies (2024) nanoporetech/dorado. https://github.com/nanoporetech/dorado [July 31, 2024]

[B59] Parisi F, Pioli S, Lombardi F et al. (2018) Linking deadwood traits with saproxylic invertebrates and fungi in European forests - a review. iForest - Biogeosciences and Forestry 11: 423. 10.3832/ifor2670-011

[B60] Pouska V, Macek P, Zíbarová L (2016) The relation of fungal communities to wood microclimate in a mountain spruce forest. Fungal Ecology 21: 1–9. 10.1016/j.funeco.2016.01.006

[B61] Purahong W, Wubet T, Krüger D et al. (2019) Application of next-generation sequencing technologies to conservation of wood-inhabiting fungi. Conservation Biology 33: 716–724. 10.1111/cobi.13240 30350883

[B62] Purahong W, Wubet T, Lentendu G et al. (2018) Determinants of deadwood-inhabiting fungal communities in temperate forests: molecular evidence from a large scale deadwood decomposition experiment. Frontiers in Microbiology 9: 2120. 10.3389/fmicb.2018.02120 PMC6158579 30294306

[B63] R Core Team (2024) R: A Language and Environment for Statistical Computing. R Foundation for Statistical Computing, Vienna, Austria. https://www.R-project.org/

[B64] Renvall P (1995) Community structure and dynamics of wood-rotting Basidiomycetes on decomposing conifer trunks in northern Finland. Karstenia 35: 1–51. 10.29203/ka.1995.309

[B65] Rieker D, Runnel K, Baldrian P et al. (2024) How to best detect threatened deadwood fungi – Comparing metabarcoding and fruit body surveys. Biological Conservation 296: 110696. 10.1016/j.biocon.2024.110696

[B66] Rolstad J, Sætersdal M, Gjerde I et al. (2004) Wood-decaying fungi in boreal forest: are species richness and abundances influenced by small-scale spatiotemporal distribution of dead wood? Biological Conservation 117: 539–555. 10.1016/j.biocon.2003.09.008

[B67] Ronold EK, Kauserud H, Nordén J et al. (2026) Long-term shift in community composition of deadwood fungi after clear-cutting.: 2026.01.20.700536. 10.64898/2026.01.20.700536

[B68] Runnel K, Drenkhan R, Adamson K et al. (2021) The factors and scales shaping fungal assemblages in fallen spruce trunks: A DNA metabarcoding study. Forest Ecology and Management 495: 119381. 10.1016/j.foreco.2021.119381

[B69] Scali E, Johnson M, Emiliani G et al. (2025) Not seeing the tree for the Forest: Scattered trees can be unexpected hotspots of fungal diversity. Biological Conservation 303: 111020. 10.1016/j.biocon.2025.111020

[B70] Simpson GL (2025) permute: Functions for Generating Restricted Permutations of Data. https://CRAN.R-project.org/package=permute

[B71] Stokland JN, Siitonen J, Jonsson BG (2012) Biodiversity in Dead Wood. Cambridge University Press, 525 pp. 10.1017/CBO9781139025843

[B72] Tedersoo L, Anslan S (2019) Towards PacBio-based pan-eukaryote metabarcoding using full-length ITS sequences. Environmental Microbiology Reports 11: 659–668. 10.1111/1758-2229.12776 31219680

[B73] Tedersoo L, Tooming-Klunderud A, Anslan S (2018) PacBio metabarcoding of Fungi and other eukaryotes: errors, biases and perspectives. New Phytologist 217: 1370–1385. 10.1111/nph.14776 28906012

[B74] Tedersoo L, Bahram M, Zinger L et al. (2022) Best practices in metabarcoding of fungi: From experimental design to results. Molecular Ecology 31: 2769–2795. 10.1111/mec.16460 35395127

[B75] Thomaes A, Van de Kerckhove P, Van Calster H et al. (2024) Assessing coarse woody debris by integrating full area sampling and line intersect sampling: Combining the best of both worlds. Forest Ecology and Management 562: 121943. 10.1016/j.foreco.2024.121943

[B76] Toljander YK, Lindahl BD, Holmer L et al. (2006) Environmental fluctuations facilitate species co-existence and increase decomposition in communities of wood decay fungi. Oecologia 148: 625–631. 10.1007/s00442-006-0406-3 16538482

[B77] Uhl B, Krah F-S, Baldrian P et al. (2022) Snags, logs, stumps, and microclimate as tools optimizing deadwood enrichment for forest biodiversity. Biological Conservation 270: 109569. 10.1016/j.biocon.2022.109569

[B78] Vandekerkhove K, Vanhellemont M, Vrška T et al. (2018) Very large trees in a lowland old-growth beech (*Fagus sylvatica* L.) forest: Density, size, growth and spatial patterns in comparison to reference sites in Europe. Forest Ecology and Management 417: 1–17. 10.1016/j.foreco.2018.02.033

[B79] White TJ, Bruns T, Lee S et al. (1990) Amplification and direct sequencing of fungal ribosomal RNA genes for phylogenetics. In: Innis MA, Gelfand DH, Sninsky JJ, White TJ (Eds) PCR protocols: a guide to methods and applications. Academic Press, New York, 315–322. 10.1016/B978-0-12-372180-8.50042-1

[B80] Wickham H (2016) ggplot2: Elegant Graphics for Data Analysis. Springer-Verlag New York. https://ggplot2.tidyverse.org

[B81] Wickham H, Averick M, Bryan J et al. (2019) Welcome to the tidyverse. Journal of Open Source Software 4: 1686. 10.21105/joss.01686

[B82] Yang S, Limpens J, Sterck FJ et al. (2021) Dead wood diversity promotes fungal diversity. Oikos 130: 2202–2216. 10.1111/oik.08388

