# Supplementary material for: Tree identity shapes composition, substrate shifts dominance in deadwood fungi of an old-growth lowland beech forest
Source: IMA Fungus. 2026 Aug 24;17:e190198. doi: 10.3897/imafungus.17.190198 (PMC13531028; doi:10.3897/imafungus.17.190198)
Supplement: Supplementary file 2 [file imafungus-17-e190198-s003.zip › krona_aFWD_perSamplePct.html]

Javascript must be enabled to view this page.

magnitude
magnitudeUnassigned

krona\_aFWD\_perSamplePct

3399995

30

30

30

30

30

30

30

535

535

535

535

535

535

535

3397350

35

35

35

35

35

6

29

219

210

210

210

210

210

9

9

9

9

9

70975

70975

70975

70975

70975

70975

30

30

30

30

30

30

1823474

1485178

239

17

17

17

222

222

222

5132

3098

3098

733

8

2357

1350

1350

1350

684

603

2

601

81

81

617

617

472

55

417

135

27

108

10

10

14929

14333

14333

14333

596

596

596

27

27

27

27

1418

6

6

6

1412

1412

1412

1

1

1

1

87

87

84

84

3

3

994

994

994

994

1404

1404

1404

1404

12233

12233

2385

2361

24

704

704

8798

3221

228

5347

2

346

344

2

9906

9906

1

1

9905

9905

764

114

114

114

500

484

3

479

2

16

16

150

150

150

57215

57215

396

9

382

5

1123

1123

1154

399

750

5

64

64

54478

54478

6771

6771

6771

60

6711

1161074

24027

24027

24027

240089

275

275

1

1

222533

222533

17280

17280

17368

17368

17368

13962

6

6

12957

12957

819

819

180

1

179

865628

118697

118490

207

746931

306891

252638

187399

3

124759

6

6

6

72902

24

24

72878

718

49774

178

179

3100

18929

724

724

724

43304

4

4

16527

16527

507

7

5

457

11

27

26160

26160

55

55

51

51

4587

2

2

21

21

277

277

4287

4287

5

5

5

569

569

569

2662

2655

80

2

2573

7

7

7

7

7

7

11303

11303

10764

10764

72

29

43

159

71

72

10

6

308

308

983

94

94

8

86

752

752

752

44

44

44

93

93

93

960

958

958

958

2

2

2

74355

74355

74355

529

531

71510

2

1783

55025

49864

302

229

229

73

73

1591

44

44

183

183

1364

1364

1985

1985

1985

368

368

368

31

31

31

5800

94

94

5706

350

5356

11804

11804

11804

173

173

173

116

116

116

1097

1097

1097

122

119

119

3

3

20168

20168

20167

1

2

2

2

15

15

15

22

22

22

56

56

56

46

46

46

8

8

8

6158

6147

6127

20

11

11

1136

1136

1136

1136

527

527

527

527

41

41

41

41

2956

133

133

133

3

2

2

1

1

677

2

2

19

19

643

643

13

13

2143

2102

2102

41

41

435

20

20

20

415

415

415

66

66

66

66

23409

9

9

9

9

214

7

7

2

1

2

1

1

207

129

129

78

78

81

81

81

81

22879

22556

20

20

14516

13

923

6

170

13404

1

1

226

5

37

180

4

6505

6505

100

54

46

1188

1114

74

323

323

323

226

226

222

222

4

4

2657

2657

2657

2657

2657

34591

34591

2366

2366

2366

157

157

155

2

18

18

18

31319

31319

31319

683

595

31

564

88

88

48

48

48

219498

198410

1428

1428

1428

27

27

27

1758

1758

1758

76

76

76

8406

8406

8406

4

4

4

90

31

31

59

59

4020

127

111

16

45

45

3631

3531

97

2

1

45

45

172

172

4

4

4

112851

36778

36778

76064

4612

95

71357

9

9

102

102

102

119

119

119

43797

37227

169

36782

276

6570

6570

8988

8988

8988

16740

16740

66

16570

104

17880

17880

17863

17862

1

17

17

6

6

6

6

3197

3197

3197

3197

5

5

5

5

891

891

891

8

2

6

882

149

100

592

1

40

1

1

2178

4

4

4

4

626

477

292

292

185

185

6

6

6

143

143

143

70

70

70

70

1468

1468

1468

1468

10

5

5

5

5

5

5

3

3

3

3

3

44

44

44

44

44

130

130

130

130

90

2

28

58

2

30

30

10

1

7

2

1502487

1710

84

84

72

72

12

12

1589

550

369

335

34

15

15

166

166

33

33

33

279

279

279

123

123

56

67

398

398

292

106

45

45

45

161

161

47

114

21

21

19

19

2

2

12

12

12

12

4

4

4

4

5

5

5

5

5

62466

62466

62466

70

2

68

62396

9

59143

3244

785

756

635

62

62

566

565

1

7

7

121

11

11

110

110

29

29

28

28

1

1

109

65

65

65

65

3

3

3

3

2

1

1

1

1

1

1

39

39

39

39

1432211

39904

4790

4790

4790

9

9

9

34848

34848

246

34438

79

85

257

256

256

1

1

2

2

2

2

171

171

171

171

249324

55391

55391

13603

41788

193905

193905

193905

28

25

13

8

4

3

3

100

100

3

3

97

97

12

12

8

8

4

4

846027

16417

1

1

16416

5014

11398

4

12

12

12

23106

23106

23106

41

3

3

1

1

2

2

35

35

85191

85191

85191

27

10

1

9

5

5

12

3

9

253369

24707

24707

165685

165685

62977

62977

165856

23

23

165833

47429

1

11740

106171

165

327

24006

8174

8174

15832

15832

1

1

1

3172

3146

3146

21

21

5

5

5

5

5

36

28

23

5

8

8

1

1

1

5

5

5

274782

274782

274782

133466

5

5

5

3705

3702

3702

3

3

23

16

16

7

7

3517

3517

3128

389

130

130

130

911

911

911

34447

34447

34419

28

90728

11847

11847

37070

37070

2

2

41809

41809

471

471

428

428

16

16

27

27

148765

144520

144520

9

144511

78

78

78

545

519

519

26

26

4

4

4

3618

3220

3220

218

218

180

36

144

6298

6298

6298

6298

6

1

1

1

5

5

5

7644

1454

1453

1398

55

1

1

93

93

93

4

4

4

5148

5148

5148

945

945

7

4

934

21

21

18

18

3

3

5192

5192

5192

5192

2

4889

301

9

9

9

9

9

0

0

0

0

0

0

0

2080

2080

2080

2080

2080

2080

2080
