# Supplementary material for: Tree identity shapes composition, substrate shifts dominance in deadwood fungi of an old-growth lowland beech forest
Source: IMA Fungus. 2026 Aug 24;17:e190198. doi: 10.3897/imafungus.17.190198 (PMC13531028; doi:10.3897/imafungus.17.190198)
Supplement: Supplementary file 2 [file imafungus-17-e190198-s003.zip › krona_fFWD_perSamplePct.html]

Javascript must be enabled to view this page.

magnitude
magnitudeUnassigned

krona\_fFWD\_perSamplePct

3499991

10952

10952

10952

10952

10952

10952

10952

0

0

0

0

0

0

0

3488983

1708321

1577542

1

1

1

1

1

1

1

1

31990

1282

1282

1282

30708

30708

30708

4

4

4

4

51054

50216

50216

18

16296

33902

722

722

722

2

2

2

114

11

11

64

4

60

39

35

4

169567

31

30

13

17

1

1

111

111

111

47921

1315

1315

476

476

46130

46130

64

64

58

6

54906

54906

54906

106

106

106

2524

2524

2524

63904

4368

1467

2901

3

3

39245

39245

20288

20273

3

12

2043

2043

1889

1889

154

154

796580

4

4

4

170243

170243

2

569

4

1

77

62

136188

29390

3941

7

2

1

1

1

1441

1441

1441

147568

4

4

857

857

145159

145159

1548

1548

14

14

14

231918

91579

91579

13135

12320

1

813

1

82660

82660

43910

43910

634

634

3

3

3

8424

8424

8424

20

20

20

170808

170808

170808

383

331

3

53

275

52

52

18223

1

1

18222

18222

1410

1410

1410

31634

31634

94

2

19

31519

14188

14188

14188

227

227

227

71

27

27

44

44

39422

31436

31436

31436

191

191

191

2307

2307

4

2303

5488

5488

5488

1630

1630

1630

1630

156475

639

639

638

1

11144

11144

11144

98130

97492

97492

638

638

28366

28366

28366

18196

16467

16467

1719

36

1665

18

8

8

2

2

77089

77089

77089

77089

36334

36334

36334

36334

215352

59981

59981

59981

44310

44310

44310

111052

8105

8105

102947

102947

9

9

9

105

105

105

105

105

38946

38946

38946

38946

38946

3045

3045

3045

3045

2523

522

1703

1692

21

21

21

588

588

4

584

6

6

4

2

444

6

6

438

438

506

506

506

83

83

83

44

44

44

5

2

2

2

3

3

3

6

6

3

3

3

3

4186

6

6

6

6

4

4

4

4

4167

4167

1

1

4166

4166

9

9

9

9

601

45

9

4

4

5

5

36

36

36

23

23

23

23

2

2

2

2

528

359

359

359

162

162

105

57

7

7

7

3

3

3

3

87

87

87

87

87

82106

82106

82106

35508

35508

46598

46598

1762914

52544

1290

1290

1290

1290

201

201

43

43

158

158

3

3

3

3

51016

1

1

1

160

160

160

50844

299

250

49

2052

76

8

1707

65

76

98

2

20

39212

14

6692

294

4050

28162

2517

2517

4186

551

3635

1201

1201

1225

577

648

3

3

149

149

11

11

11

34

7

7

5

2

27

27

27

53459

6

4

4

4

2

2

2

453

3

3

3

371

23

23

348

348

13

13

13

2

2

2

64

23

23

27

27

14

14

433

433

433

433

51385

19025

19025

18994

31

20

20

20

17

17

10

7

5

5

5

61

61

61

27

24

24

3

3

182

90

46

44

92

19

73

413

3

3

410

406

4

3

3

3

1741

1741

1741

109

109

109

1641

1641

1641

109

109

109

23111

23111

23111

4

4

4

1694

2

2

66

66

2

2

1624

1624

2

2

2

7

7

7

3042

3042

3042

172

1

1

5

5

157

66

91

9

9

134

134

134

134

260

260

260

260

485

485

485

485

74

74

74

74

217

56

56

56

161

161

161

12

12

12

12

4029

6

6

6

6

158

8

8

8

150

5

5

93

93

52

31

21

3865

3865

3865

3865

2772

2772

2771

172

172

559

555

4

1

1

2039

340

454

46

1

648

519

14

17

1

1

1

1198350

1911

1911

535

3

28

504

790

19

771

8

8

578

578

2481

2481

2481

2478

3

22196

295

295

293

2

7451

16

16

7431

3

7428

4

4

14450

14450

7608

6802

40

29293

29293

16775

16775

12518

12518

2346

2346

2346

2346

82431

82431

82431

457

5

2590

6677

71882

820

9

7

7

7

2

2

2

25266

25266

24600

24600

22

22

66

66

497

369

117

11

81

79

2

66415

66415

3

3

1

1

2

2

61627

61627

12

12

2625

285

1574

2

764

2129

4

63

2062

16

16

3

3

3

3

14

14

14

14

9130

804

804

804

8326

8326

8326

10356

10356

10356

10356

5302

543

543

543

4759

4759

4755

4

6649

6649

5824

732

5092

125

125

8

8

98

98

594

6

395

193

706489

616

616

616

29418

29418

29418

528587

172032

172032

2

2

356553

149210

192014

13087

2242

121426

3516

3516

22800

22800

95110

95110

693

693

693

25749

46

46

99

19

80

4662

4662

20942

20942

2

2

2

2

75

75

75

75

26094

5

1

1

4

4

362

76

15

61

286

286

15893

19

19

15874

2

13635

396

75

73

1691

2

135

135

135

9678

30

30

39

39

71

71

4347

4347

2

2

172

172

4990

4990

27

2

25

11

1

1

10

10

8

6

6

2

2

2

2

2

147463

39956

39956

39956

85068

85068

85018

50

2731

2731

2731

19706

19703

7

19696

3

3

2

2

2

4

4

4

4

7265

7263

2146

2146

5117

5117

2

2

2

47156

32670

32570

6

595

163

122

31684

100

100

14486

14486

14486

81232

81232

81232

81232

81232

6

6

2

2

2

4

4

4

31

31

31

31

31

351228

27

27

25

25

2

2

95563

97

97

97

95466

91611

184

90272

912

243

11

11

2

2

3842

3842

242811

45985

28427

16605

17

3

11802

17558

17558

384

2

2

13

13

231

231

134

134

4

4

3748

3748

3748

3463

855

855

653

653

1955

1548

407

69

69

69

2

2

2

9317

9317

9317

62101

57328

57328

4773

390

4273

36

74

107

107

107

1

1

1

9541

87

87

511

4

379

128

570

570

12

7

5

8324

8324

37

37

1231

1231

1231

49908

49908

49908

4319

4263

4263

56

53

3

34428

5

5

34423

28

34393

2

18207

3

3

4

4

18200

18200

12827

12827

12827

12827

17569

17569

10142

10142

9455

687

7427

7334

7334

93

93

1692

1692

112

112

112

5

2

2

3

3

4

1

1

3

3

1571

748

748

823

823

2

2

2

2

2

94

8

8

8

8

8

17

17

17

17

17

9

9

9

9

9

60

60

60

60

60

2658

1955

1955

1955

1955

1955

703

19

19

19

19

665

665

665

665

19

19

19

19

14856

14856

14856

14856

14856

14856

140

140

140

140

35

35

55

27

8

18

2

50

29

9

12

56

56

56

56

56

56

56
