# Supplementary material for: Tree identity shapes composition, substrate shifts dominance in deadwood fungi of an old-growth lowland beech forest
Source: IMA Fungus. 2026 Aug 24;17:e190198. doi: 10.3897/imafungus.17.190198 (PMC13531028; doi:10.3897/imafungus.17.190198)
Supplement: Supplementary file 2 [file imafungus-17-e190198-s003.zip › krona_full_dataset.html]

Javascript must be enabled to view this page.

magnitude
magnitudeUnassigned

krona\_full\_dataset

162355470

2749

2749

252

152

152

152

152

100

59

59

59

41

41

41

2497

2497

2497

2497

2497

440852

440852

440852

440852

440852

440852

440852

161815597

68837098

56971

140

140

140

140

271

271

271

271

1652

1652

1652

1631

21

116

89

89

89

27

27

27

54792

54792

4656

2026

2630

50136

50136

174

11

11

11

11

163

163

163

143

20

383687

383687

383687

383687

383687

15

15

15

15

15

2305

2214

1747

1747

1747

467

467

256

35

80

96

12

12

12

12

14

14

14

14

65

65

65

65

136631

104087

98032

98032

98032

6055

885

885

3243

139

3104

1927

566

1361

5522

5522

5522

5522

18

18

18

18

24911

14479

37

37

12517

2771

9746

1925

1925

689

689

689

9488

45

45

576

576

8867

7586

1281

255

255

245

10

2093

2093

1980

17

10

1953

113

113

173447

56

56

56

56

173391

173391

173391

20

21

55823

117527

452489

122169

18037

3576

3576

3028

2776

252

6356

6356

5077

5077

12531

31

17

14

12500

3419

9066

15

17338

17338

17338

12

12

12

4225

532

532

3693

3693

2560

2560

1745

815

604

604

534

70

44814

601

601

44213

43534

36

619

24

22048

22048

21368

680

48585

48585

48585

48585

260227

228258

15095

15095

178336

178234

102

34756

34756

71

71

31969

31556

31517

39

413

413

17145

17145

10049

10014

35

50

50

38

38

7008

6643

365

4363

4363

3652

32

3620

711

711

1567

1401

662

440

205

235

222

19

203

739

739

739

166

166

166

166

66030113

9602423

288003

238175

238175

696

696

49132

49132

18603

18603

18603

2876681

406073

8526

397547

583170

26

112968

470176

25466

25466

1861972

1861972

134304

95741

95741

38243

38243

320

135

185

97

79

37

42

18

18

961352

961352

945902

174

277

14050

949

3855814

960

884

76

817

817

1161552

1161552

95229

95229

669

669

4504

4504

2592083

2592083

1252615

1252615

241937

1010541

43

94

89180

89180

40

89059

81

52108

52108

52108

6500

6500

6500

33499

786

786

32713

32713

12566

44

44

12522

12522

21101

21101

21101

485

485

319

319

137

137

29

29

38989202

54791

54791

54791

6420

2428

812

14

88

1514

3992

3354

224

414

2030101

130

130

655319

654915

48

10

331

15

8104

11

8013

45

35

11

11

134562

81661

14

199

37

159

522

122

444

51404

685653

685653

546322

546322

61217

61217

61217

277

25

25

158

158

77

77

17

17

234849

234849

1193

224375

601

43

256

132

66

553

7630

154382

154382

1197

5269

105

147811

6660264

156

156

1225

1225

6658865

92

1803307

51156

1087

87

381

2945

2781

18810

75

7982

122

367190

104

1532883

74

8700

2860844

245

18

18

102815

53

53

102762

44

102718

1361764

1361764

1361764

2838704

63

63

39

39

2837896

2837896

706

706

67637

76

76

67561

67561

524

524

524

11690

46

46

11430

2025

9392

13

214

214

262

262

177

85

1422242

77507

77494

13

31

31

1344704

149

15

1344540

11

11

11

154828

208

14

40

154

22

22

151973

2047

230

45734

103962

2625

2625

196

136

136

10

10

50

50

22178

22157

10

3974

3684

111

171

14207

21

21

848875

11

11

32207

32207

24

24

23

23

379

379

1050

1050

1198

1198

3476

3476

809944

809944

542

542

21

21

368

324

324

10

10

34

34

1759

10

10

1749

11

1120

576

42

336

336

336

8297029

65457

65416

41

11

11

1921851

1921851

6309710

10

6309700

240

240

240

15948

15948

13452

1031

875

162

428

296005

296005

855

295150

14033069

25

25

13344

13344

1134170

1134170

294547

294547

12590961

915

12590046

22

22

21

21

21

309410

305235

305235

4175

4175

990

228

228

762

762

398290

397833

382

382

85

85

14362

287

123

187

1216

12549

32032

57

15174

16801

350972

350972

457

457

457

449000

16620

16063

16063

519

519

38

38

9216

9216

9216

423164

38

38

423126

423126

1197212

1197212

1197212

1197212

4367260

1293737

1240488

1240478

10

53249

53249

285661

285661

285661

1606123

1296958

2316

42

10997

601430

241

2010

10893

13

20401

5534

14510

161488

28427

709

396698

41249

309165

9882

266229

33054

1181739

1178215

162690

1015525

477

477

3047

3047

1761421

264147

264147

264147

144

144

144

1109011

1109011

1108932

10

69

69

53

53

16

16

11776

11776

11776

376274

50738

50738

12

12

37592

37592

10

10

3145

3145

37

37

284740

284713

27

239090

239090

66

66

239024

239024

453305

453305

383858

383858

550

24

14

512

68897

68897

88324

88324

88324

88324

4667812

778952

3750

3750

771850

771850

3352

3352

1813941

104

104

1813759

1336

1181776

623827

6820

78

78

148155

148155

148155

1767265

384425

572

25844

7970

350039

1726

1726

1378826

1378826

2288

2288

5769

5769

5184

585

153730

153730

153730

87416

87401

630

630

13682

13682

73089

73089

15

15

15

875452

52599

52599

52599

84

36

36

48

48

126778

126778

8990

1935

56893

2309

53321

3330

13591

13483

13483

108

108

322026

309820

280216

3164

11

12

26417

12206

46

41

12119

765

765

28

737

359609

359609

359609

2852893

314688

25657

25657

280885

275360

41

5047

437

828

828

7318

7292

26

2473908

476

476

2473383

1382471

63

389541

628487

72821

49

49

61075

49684

49684

11391

11391

31

31

31

12

12

12

3179

3179

3179

528

528

34

34

494

494

3374

708

708

708

708

2666

2666

2666

2666

1596325

1596325

1596290

385981

26157

359824

1210309

24047

5856

1180406

35

35

35

636418

636418

636418

636418

636418

636418

1355

1355

1355

1355

1355

1355

1601

1444

1444

1433

796

548

248

637

637

11

11

11

157

157

157

157

157

739

86

86

86

86

86

653

653

653

653

653

24240

3325

3325

389

361

361

28

28

2936

2936

2936

1973

1973

1973

1973

1973

2145

2145

2145

2145

2145

11393

11393

40

40

40

11353

11353

11134

219

1100

1100

1100

1100

1100

4304

4304

4304

4304

4304

3978

3978

3978

3978

622

622

3356

3356

41

41

41

41

41

41

1866

57

57

57

57

57

1415

566

566

566

566

403

403

403

403

446

446

446

446

394

394

394

394

56

338

92034723

1877899

31054

31054

17271

17271

13783

13783

403832

61380

20500

17100

45

2255

13

1087

40880

40880

309610

284505

397

87332

12

214

6136

141082

531

19657

14

622

53

3064

568

3818

154

1317

32

59

2452

3473

3266

190

1367

361

8060

274

148

148

24957

117

7612

2833

14384

11

545

545

545

32297

32297

53

32218

26

1369715

70979

58456

4062

54394

7425

7425

5098

1025

4073

1114739

49645

320

2799

175

5087

2810

20820

1168

2348

3450

10668

114710

55839

20

58851

8186

7563

623

168

132

36

57849

36542

21017

180

110

30117

30117

938

938

764890

140506

5677

19897

140253

456388

2169

88236

849

87387

10

10

10

183430

183430

183430

557

499

499

58

58

66073

66073

66073

66073

442

37

37

37

405

209

209

196

11

185

6039

6039

5007

11

4996

1032

1032

627

627

627

627

33

33

33

33

84

84

84

13

71

2999980

2999980

80688

31660

871

30789

46369

46369

2659

2659

32524

13083

82

12605

130

266

19122

53

2897

16172

55

55

138

138

126

126

37796

7843

7720

10

113

70

32

38

149

33

116

22623

22623

12

12

441

441

6418

6418

240

240

2781087

2781087

2781087

503

503

181

322

8762

8762

8762

58620

45032

45032

13588

13588

192140

192140

92434

6926

6926

17

17

6195

6195

78238

71593

307

6338

1058

1058

93496

7272

7272

70252

1643

68609

15972

15972

1516

1516

1516

4694

4694

4545

149

36

36

36

36

36

26

26

13

13

13

13

13

13

914

914

914

831

533

298

83

83

8814018

1481

117

117

117

610

610

610

754

754

754

7411816

49166

49166

3750

1781

22780

73

44

230

12338

274

7896

87849

83769

83769

4064

136

3928

16

16

162474

22

22

161760

35469

122640

2264

1387

25

25

27

27

640

640

67429

67429

67368

61

210937

210937

1770

209167

45907

28

28

59

59

6646

6646

27870

27870

4950

4950

564

564

669

669

4806

4806

315

315

116

10

10

52

52

54

54

3211

3211

3211

75668

32129

36

31672

399

22

11

11

42218

42218

1310

1310

1232725

29832

27187

1086

1505

54

5041

5041

12

12

653

653

3766

3766

292

292

111357

111357

627453

627453

454086

306

446667

4979

2134

233

233

243

243

243

15046

15046

14579

467

863438

863438

863438

1216025

816956

816956

399069

341823

599

55870

777

1562918

475483

475483

1009919

1030

771497

55670

181722

77516

44061

103

10

33163

179

19733

69

69

19664

19664

11885

11885

11885

776711

1892

1892

774819

774262

557

911494

992

992

860889

430980

7687

715

404183

17324

11193

2849

8344

38420

38420

98841

20020

20020

5560

5560

54221

54221

16722

4000

12722

2318

2318

1213801

341

341

341

947

890

890

57

57

4689

4689

4689

1207824

2077

2077

1164433

1761

1150994

1251

10427

3955

3955

37359

37359

34820

34820

13376

13376

16442

16442

4295

4295

156

156

551

551

4412

4412

4412

4412

147688

147688

147688

147688

119207

119207

11252

11252

11252

107955

83237

518

2785

8226

2368

3731

4837

306

56799

1756

11

1601

299

761

761

11030

11030

64

50

14

15

15

421

421

12427

3969

719

226

7513

1141007

1141007

1141007

1141007

1141007

183

183

31

31

31

152

152

152

678

678

678

455

94

91

270

223

223

73690844

664

664

664

664

18

18

18

18

4621160

12382

12382

12382

1207952

10518

12

11

10200

149

146

15

15

40

40

1516

1516

3392

3392

1192471

11849

22

2285

187

1564

1629

760

1521

69678

839059

195540

372

67636

13

39

317

197045

258

258

16

16

458

458

181

121

60

12

12

98967

12076

1163

85728

97153

97153

97

97

97

319562

319562

319562

74564

38

38

260

260

960

960

8907

8870

37

56500

55075

450

975

7886

5413

2455

18

13

13

411445

3224

3224

408221

71

100737

307413

35

35

12

23

120626

77

77

567

76

491

117375

117375

231

30

147

54

33

33

1343

1343

659

579

34

46

32

32

309

309

956

956

956

1773039

27597

27597

7443

7443

190500

15

471

17

189997

146446

89889

1635

36542

89

16754

103

1434

55010

1616

53394

111

111

11

11

658

658

38

38

1318770

1318770

18355

56

18299

171

171

5541

5541

2388

27

1036

1325

31

31

31

498432

201544

201544

94735

94723

12

130

130

1720

930

790

4862

189

3600

12

395

666

51233

51233

3718

3718

172

10

162

2301

2301

137933

137933

84

84

2908

2908

2908

2086

2086

39

2047

13209

34

34

34

622

622

622

12553

12553

12553

3734374

3734374

25358

25358

197627

11

164022

894

422

31893

131

254

92424

92424

82111

12471

4307

79

13

36844

28397

1314586

1314586

676

420

256

100

100

67497

67497

1953970

1953970

10

10

15

15

205285

205285

205285

205285

1015657

90505

90505

2873

87632

553883

34439

34439

518962

139052

294484

85426

482

482

371269

301806

22

142464

158834

486

69463

69463

487

487

487

487

728445

728445

728445

76289

652156

126106

126106

126106

126106

707057

2460

2460

2424

36

511513

7185

7185

504328

6471

485099

7472

2764

2522

193084

193084

193084

303

137

137

137

166

142

142

24

24

1098571

1098571

346405

346405

689492

638931

50561

62674

43177

19497

52447716

12082445

9138176

123828

3636430

1750802

3627116

211

211

2944058

588

2943470

635

635

635

16

16

16

125529

125529

125529

39

39

39

26821128

165

165

26379331

26379331

77925

54245

826

33

398

22423

363659

1589

362070

48

48

4263

4239

4239

24

24

365511

365511

365511

13045283

247844

247844

12710032

12225163

484869

44303

44303

163

163

42941

42941

2867

2867

73

2794

624954

624954

624954

624954

65625

7737

7737

7737

57888

57888

55988

399

1501

12414

53

53

53

1577

1577

323

1111

118

25

10784

13

13

10

10

342

342

9926

9926

183

30

153

310

310

1877052

1877052

1877052

10395

60

109667

1620040

17410

119480

104558

3827

3827

65

139

3623

100731

68838

68838

31893

31893

2613911

939138

939138

939138

2112

701

701

1411

1411

770154

770154

764454

5700

410562

22

22

9863

9863

400677

400624

53

258

258

258

48

48

48

474452

474452

474452

17187

17187

17055

132

111644

8038

8038

8038

103606

103606

103606

62267

62267

62267

62267

2326125

2326125

748714

9860

732075

837

988

97

4857

15199

15199

186413

19

72

186322

1344833

1344833

67

67

30899

102

30797

280551

280551

163430

158826

4604

117121

117121

750

750

750

634

89

27

270250

270250

80430

9346

3735

67201

148

135235

750

71987

62498

36472

23322

13150

9254

9254

8859

8859

156

156

156

156

166543

166543

62816

3676

51855

7285

5124

5124

73536

49746

22249

1541

4221

3436

785

23

23

20823

20823

474992

399962

399962

385300

14662

21626

88

88

144

144

18473

18336

137

64

64

19

19

2838

2838

169

169

169

53147

202

202

553

553

52392

52392

13

13

13

17

17

17

58

58

58

2735565

165748

165682

165682

165682

66

66

66

10

10

10

10

57

57

57

57

1390

29

29

29

1361

362

362

999

999

6022

6022

5239

5239

783

783

1207368

6713

801

801

1911

1911

409

409

3367

103

3264

225

225

96

11

11

85

21

64

237391

237391

372

237019

24750

94

94

6095

6095

93

93

17

17

18

18

17966

17966

467

438

29

34531

34531

34531

805

805

805

367

367

367

77

77

77

1781

46

46

1735

1735

714

714

521

193

894

894

894

526

526

475

51

14

14

14

95

95

95

7704

7302

7302

184

184

218

218

126508

126508

126508

5709

4992

4992

717

104

613

1357

1357

1357

65796

2740

2740

387

387

5534

5534

57135

603

18

52515

3999

283209

283209

283209

1488

1430

12

45

1357

16

28

28

30

30

258638

258638

258410

228

5234

5234

5234

49

12

12

37

37

4289

3515

10

3178

327

774

144

630

851

851

851

84918

84918

11169

73749

48619

132

132

48487

47975

179

333

152

152

131

21

3622

3622

3622

471

471

471

17

17

17

17

47

47

47

47

17899

17899

17899

16716

1183

446

446

446

446

4930

1664

1638

1638

26

26

1972

1972

1972

1294

1132

1132

126

126

36

36

3624

3624

3624

3624

583

583

462

462

121

121

41330

41330

41330

41330

14

14

14

14

73

73

73

73

636168

633050

633050

633050

12

12

12

3106

3096

3096

10

10

649839

1520

1520

1520

84870

88

64

24

79362

79362

221

80

141

35

35

15

15

4895

4895

11

11

66

66

177

177

479831

501

501

473057

473057

616

479

137

5657

5657

3241

2826

2826

10

10

405

405

80377

80340

21

80177

15

127

37

37

432

432

388

311

311

77

77

44

44

44

193281

3456

3456

3456

3456

1022

1009

1009

1009

13

13

13

699

699

699

699

69

69

69

69

167656

167656

167656

167656

1900

1900

1900

1900

217

217

217

217

17046

970

970

970

12154

36

36

8677

4858

79

3740

3441

295

3146

10

10

10

1239

1239

216

352

163

280

228

2673

2673

2673

25

25

25

25

411

314

294

294

20

20

97

97

97

780

780

780

780

268495

253960

253960

253960

253960

14535

14535

326

326

1042

1042

12415

12415

261

261

491

491

18

18

18

18

18

139859

139859

139859

139346

156

156

2067

2067

2115

2115

65489

46958

1023

1956

281

1158

50

477

1511

155

2318

3861

5741

4949

4949

7277

7277

57026

13648

682

39759

24

1418

1495

182

182

85

85

513

513

513

75

75

75

75

75

75

133604

20186

1158

1158

1158

1158

13925

13925

13925

13925

4746

4746

4746

4746

357

357

357

357

113418

113418

113418

113418

113418

65970

65970

65970

65970

65970

65970

65970

1506

48

38

38

38

38

38

10

10

10

10

10

347

183

183

183

183

183

164

164

164

164

164

1111

975

975

975

975

975

136

136

136

136

136

0

0

0

0

0

0

0

28796

108

108

108

108

108

108

28688

8641

8641

8641

8641

8482

159

2140

2140

1827

1827

1827

313

313

313

16580

1185

1029

1029

1029

156

156

156

1133

459

459

459

674

674

674

14262

14262

14262

14262

1327

1327

1327

1327

1327
