# Supplementary material for: Tree identity shapes composition, substrate shifts dominance in deadwood fungi of an old-growth lowland beech forest
Source: IMA Fungus. 2026 Aug 24;17:e190198. doi: 10.3897/imafungus.17.190198 (PMC13531028; doi:10.3897/imafungus.17.190198)
Supplement: Supplementary file 2 [file imafungus-17-e190198-s003.zip › krona_full_dataset_perSamplePct.html]

Javascript must be enabled to view this page.

magnitude
magnitudeUnassigned

krona\_full\_dataset\_perSamplePct

26400014

11730

11730

11730

11730

11730

11730

11730

103399

103399

103399

103399

103399

103399

103399

19574

93

93

93

93

93

93

19481

10171

8091

8091

8091

8091

1477

83

83

83

1394

1394

1394

603

359

359

359

244

244

244

1427

1427

167

167

167

1260

1260

1260

7111

7111

7111

7111

85

7026

772

772

772

772

772

26261822

14205873

123629

123629

123629

123629

123629

53

53

46

11

11

35

35

7

7

7

129

129

129

108

26

9

73

21

21

441261

94

94

94

94

3383

3383

3383

3250

133

1

1

1

1

152102

1733

1733

1733

21456

21456

1882

19574

31203

31203

31172

31

1

1

1

233

3

3

227

2

217

1

7

3

3

1513

67

10

57

1446

1446

127

127

127

48

48

46

2

297

297

297

6618

14

14

6604

6538

24

42

184

184

184

4478

9

9

1678

1678

68

2

66

2695

2695

2

2

9

9

17

17

80

80

80

326

311

311

15

15

10

8

8

2

2

84

84

50

34

7913

51

51

831

831

6664

5625

235

7

797

367

367

125

125

125

143

143

143

13

13

13

15

15

15

22210

22210

22210

71

71

64

7

544

114

19

95

430

44

1

385

4117

4117

4117

1364

24

24

1319

1319

21

21

23413

23413

23413

22091

22091

107

21984

1558

67

67

98

98

864

48

816

456

456

73

73

25

8

8

17

3

14

109

109

109

63

63

13

13

50

50

147731

146

146

146

10895

1

1

15

15

19

12

7

1

1

3

3

624

624

10218

10218

8

2

6

6

6

129777

72

72

1132

1132

87

52

35

128486

128486

317

271

271

8

8

38

38

6596

4

4

6592

2

15

6574

1

10

10

10

10

209

199

65

65

134

134

10

10

10

485

485

485

485

100928

440

439

439

1

1

1

1

1

100487

100487

100487

929

929

63

63

866

866

5

5

5

5

25

25

25

25

2

2

2

2

7753

7753

7753

7753

882

290

4

4

286

286

389

389

389

203

5

5

22

22

176

176

26657

26635

26635

26635

22

22

22

2

2

2

2

2

2

2

2

2

42

42

38

38

38

4

4

4

9

9

9

9

9

198

198

198

71

71

127

49

78

20976

49

49

49

49

1057

1057

1057

1057

94

94

94

94

41

31

2

2

29

29

10

10

10

4

4

4

4

2084

1339

966

11

588

367

368

34

334

5

5

135

135

135

1

1

1

308

308

308

301

301

84

24

137

26

30

177

177

177

177

10

10

10

10

121

121

121

121

131

2

2

2

129

129

129

17208

17208

17208

17208

4

4

2

2

2

2

2

2

10772662

44738

44738

44738

44738

410

410

410

410

632393

14

14

14

150814

11

11

148861

568

147

2881

20599

63

404

88573

102

28

11

3

179

287

5717

29297

2

1178

6

1082

1

26

63

5

5

440

440

319

319

426

426

426

1403

1403

1403

4

4

2

2

42425

439

439

41986

12721

7

29258

69446

956

23

154

63

714

2

18

18

154

63

91

32063

2

32061

12674

12674

318

318

55

8

47

22

22

18596

18596

4292

4292

298

298

256692

33562

9496

10438

638

22

12796

161

11

162449

162449

923

757

34

132

13

13

1396

1396

1017

1017

4

4

2370

2363

7

14233

14233

34547

79

3

34464

1

16

16

1

1

201

201

5960

5792

168

14702

28

28

419

419

2

2

9976

112

9734

130

2600

2593

7

1671

2

1037

632

6

6

20673

14

14

3

3

115

115

80

12

68

269

269

19999

19999

72

62

7

3

3

3

118

5

6

107

28442

146

146

37

31

6

28

28

15942

14092

1712

138

2

2

12286

12286

1

1

46956

46956

46956

179

179

179

213

213

5

208

4

4

4

116158

37416

5687

5687

31729

12473

40

8

19208

14385

14385

13005

1380

64357

4765

4765

113

113

59479

14933

36445

8101

112422

112422

112422

101833

10589

18261

18261

18261

18261

50354

35285

34064

163

889

31792

279

941

1221

1221

14671

14671

14671

398

398

4

394

8295

6883

6883

185

6571

127

1412

1412

1412

33

13

13

13

20

18

18

2

2

170974

170974

170974

5

2251

144238

13905

822

9753

35040

35040

20981

19663

1318

14059

14059

25722

25722

13216

680

12093

443

8631

168

2942

5521

1410

1410

3

3

1975

1975

487

364

123

1848

1697

17

3

14

1

1

39

39

394

394

1244

1244

2

2

7

7

7

144

144

98

16

28

2

52

52

52

52

12987

810

810

810

12177

12177

12177

431330

431330

11139

11139

89

53

36

24692

6

152

31

19082

102

50

5269

8827

8827

28

28

8917

8917

3

3

224909

224909

17803

110

1955

771

2

5671

9294

134922

134922

1

1

7762

7762

7762

7762

1290

1210

1210

1210

77

77

77

3

3

3

306280

109716

109716

109716

87276

87276

621

86655

45203

43889

7

43882

1312

1312

2

2

59008

59008

59008

439

272

272

167

167

4553

4553

4535

18

48

48

48

37

37

37

2

2

2

2

88604

88604

88604

88604

22762

1262

1262

22

46

1194

21500

19342

19342

2158

2158

172

172

172

161

3

8

133855

19

19

19

7829

7233

7216

17

25

25

534

534

28

28

6

6

3

3

5

5

5

7

7

7

2

2

2

26195

112

112

52

52

26031

26031

99798

99798

12469

87329

1291

1291

1291

1291

54652

54652

23468

74

7956

15438

1536

1536

20461

15710

1174

16

3561

712

712

8475

3201

5274

8097558

681

8

8

673

673

60

60

60

1

1

1

8

8

8

2409564

2365269

2304398

60871

21284

21284

18296

18296

51

51

4664

4664

3969185

50173

41915

8258

3880054

3880054

4

4

19

19

38935

5

3552

132

35178

68

436

436

391

45

1645086

319877

319670

207

440

440

1324769

419207

341472

13092

550998

24876

24876

24876

47661

47661

47661

133401

133401

72149

7834

64315

52806

52806

8446

1569

6877

264016

264016

81965

878

272

1696

78921

187

11

3786

3786

10

10

4348

4337

11

19658

19252

7

399

154249

154249

31489

2220

2220

40

40

122

122

122

122

1859

1859

77

77

29269

29269

29269

29269

1550782

21444

21444

21444

21444

159875

28

28

28

900

900

900

141

127

127

14

14

158806

660

660

153500

192

152151

912

245

787

787

3859

3859

1399

1399

1399

1399

1356385

20297

27

27

20270

20270

27

27

27

251237

1122

371

751

6450

6450

243522

84857

544

152197

5086

838

143

143

126845

68488

68488

58357

47270

309

10695

83

11

5

5

5

5

1

1

8101

4

4

51

51

51

51

3862

3862

1659

1659

5

5

122

122

681

681

1666

1666

170910

835

835

64832

63826

147

239

620

82

82

4026

97

152

6

3771

2

2

84647

84647

87

87

15249

15249

99

99

1051

1051

3016

3016

3016

9861

9861

9855

6

11621

17

17

936

918

18

10668

10668

9733

5157

5157

162

162

4413

5

53

3

4352

1

1

16366

2087

1664

423

9183

9183

1042

1042

787

787

3267

3267

193215

398

398

192817

52

192765

119765

119765

119765

24946

24834

447

18219

6001

167

3

3

98

98

4

4

7

7

9475

9475

4028

41

489

528

61

2229

2073

6

20

421

421

421

6258

6258

1772

4486

345286

93598

93598

9102

4832

4200

2

59

9

242586

92

198498

20210

23786

28994

28994

28803

191

182

103

103

103

67

67

67

12

12

12

11497

11497

14

14

7515

7515

87

87

1474

1474

2407

2407

239007

623

623

464

458

6

159

159

3

3

3

3

467

467

467

467

160750

1

1

1

131965

5144

5144

6083

245

518

629

2615

31

50

332

1221

341

101

9554

9413

141

11287

7896

3251

15

125

154

154

87905

14529

757

51710

3283

17308

318

22

19

3

982

933

49

10834

5

5001

5828

193

5

5

188

188

18317

18317

18317

10274

858

351

507

705

705

8711

577

8134

4309

4309

2003

2003

2306

2306

12787

12787

12787

12787

59932

40495

36812

3601

561

2

20

1

154

8

6

346

46

52

63

43

7699

20375

383

354

693

364

58

34

112

32

1065

8

732

3621

18

773

1

509

2320

62

62

6575

6575

2

8

6565

185

185

185

12677

4876

185

4017

16

2

656

7801

7801

75

75

75

63

12

61

56

28

27

1

28

28

5

5

5

25787

25787

11029

1940

1940

1518

1518

7571

214

7357

13939

13

13

653

653

101

101

10908

49

9928

931

2264

2264

413

413

413

406

406

15

391

15841

15841

14361

148

148

2

2

1085

1085

11568

46

67

1057

34

8271

276

380

1

240

385

648

163

1488

22

78

824

564

57

57

13

1

12

1480

1480

1480

984002

984002

9339

2808

2360

20

428

15

15

6499

35

6440

15

9

12

12

5

5

2737

2737

2737

3661

735

735

40

5

35

42

42

8

4

4

977

1

14

962

1820

1820

38

38

1

1

569

569

264

305

32868

27934

27934

1282

1282

3652

3431

221

43317

40760

40760

2557

2557

891511

891511

891511

25111

25111

25111

25058

810

810

12722

209

333

757

106

232

9249

39

26

38

788

642

303

8877

193

1727

6557

148

250

2

21

21

301

301

68

68

688

688

1560

1560

11

11

53

53

53

5464

2519

2519

25

25

25

2494

2494

2383

111

1038

1038

925

925

925

113

110

110

3

3

177

177

177

177

177

586

586

586

586

586

847

847

847

847

847

297

297

297

297

297

47

47

47

47

47

47

230

230

230

230

230

230

145191

145191

145191

145191

145191

145191

6

6

6

6

6

6

98

72

72

72

72

72

26

26

26

26

26

19994

14746

14746

14746

14746

14746

5248

34

34

34

34

210

210

210

210

3365

3365

3365

3365

1639

1639

1639

1639

493

22

22

22

22

22

471

471

8

8

8

463

276

276

187

112

75

473

49

49

49

49

49

361

181

181

181

181

129

129

129

129

51

51

51

51

63

63

63

63

6

57

11858304

518

358

358

358

358

160

160

160

160

18761

7

7

7

7

18754

18754

18754

6188

2

2

12562

5115

4878

4878

4333

4333

545

204

341

48

48

48

48

14

14

14

14

12

4

4

4

8

8

8

163

163

163

2

161

287

65

65

65

65

222

128

81

79

2

47

19

28

94

94

94

56188

2370

2370

23

23

1084

5

1079

5

5

1258

1184

74

30039

23462

3433

3433

17104

17090

14

2906

2906

19

19

6577

6531

6524

7

46

46

449

449

347

344

3

102

102

5295

5295

5295

5295

18035

286

286

77

209

4715

4715

4644

71

2001

472

438

34

550

550

526

526

453

453

1578

4

2

2

1574

1147

425

2

97

97

10

87

1

1

1

5804

459

459

5345

4

116

5220

5

2251

2251

2251

1302

1219

1219

83

83

11478897

111743

96

96

96

111647

105338

105338

2139

38

1899

154

17

31

33

33

42

42

4095

7

1460

2628

9534

9534

9534

9534

41126

1330

1330

1330

1874

5

5

50

50

1819

1819

37922

37919

37919

3

3

37713

37713

37699

37699

14

14

50827

50827

42492

42492

48

42

2

4

8287

8287

737079

115878

45

45

115462

101813

13649

371

371

307208

299082

1

299081

8126

8126

269638

53508

4292

1282

47934

216130

3077

2

300

4812

32

108

14659

18078

66270

95092

2117

1555

62

1025

8321

620

44355

44355

44355

113

113

14

14

4

4

95

95

448892

5569

770

770

4799

4799

391024

64

64

6

6

390954

31565

32

192391

34186

132780

2

2

2

680

680

680

51613

594

594

45601

4

44488

1049

60

3617

3617

1801

1797

4

4

4

4

116

116

112

112

4

4

622029

950

950

728

222

249130

249106

190

176721

1433

70762

9

9

15

15

28367

28367

28367

99095

735

735

97720

97720

640

640

21718

21718

21718

222769

3357

3357

170992

170992

673

673

47747

2293

6835

38547

72

11779

11777

82

82

10069

10069

1626

1626

2

2

2

1898663

2273

2273

2273

126649

30

17

13

64670

64670

61949

61949

2524

2524

2524

144309

106

106

7255

7255

136948

136948

19830

19825

19825

5

5

6950

6950

6950

326024

206443

206443

49198

47731

1467

62575

20273

3

42299

7808

7808

25

2

2

23

4

19

91854

91854

2300

89335

145

28

46

824884

548815

548815

76627

76627

198612

198612

96

96

151

24

127

476

476

107

107

331349

331349

7

169381

49

161912

2967

2967

2967

5470

5285

5285

185

185

13555

13555

8

13

13534

270183

66768

11

11

441

441

1

1

6838

6838

7762

7762

51714

51701

13

1

1

19

7

7

12

12

163899

163899

163893

1

5

13

13

13

37370

37370

37370

2114

2114

2114

226216

226216

226216

226216

6893128

23494

23494

22065

614

110

705

8437

6

6

8431

8427

4

37730

37730

418

37312

25130

361

361

24748

8292

11398

5014

44

3

3

18

2

13

3

130818

150

150

124742

124742

4761

4761

3

3

44

44

3

3

692

692

335

335

5

5

81

81

2

2

69252

502

502

68750

68750

113

113

113

64

64

64

14

14

14

3182

3179

1

88

1875

451

14

750

3

3

111

9

9

8

8

94

94

102

82

82

20

20

1174087

341

341

404

404

1

1

1173341

1543

10

20

2243

358067

392

48502

7

247

379636

51412

327812

30

96

9

62

2711

262

280

6266

6258

6258

8

8

916

363

286

20

57

553

129

6

413

5

2319

2319

1845

177

73

16

208

1198628

1

1

196970

196970

962689

1

962688

38968

38962

6

250463

17371

171

47

1

12322

85

60

4

26

4655

649

4

636

5

4

94446

94396

1

42

1

6

94062

94062

2

2

13

13

43920

43920

63

63

48

15

472861

472687

472687

161

161

6

6

7

7

22

6

6

15

15

1

1

4

4

4

2866032

81308

81308

3

3

179430

179430

4

4

2604052

318

2603734

1235

1235

310490

310490

310490

45

45

45

25

3

3

1

1

7

7

14

14

1249

23

23

4

4

1222

227

994

1

5530

5530

5530

33444

33444

19

19

1355

31767

7

128

65

5

79

15498

15498

15498

1264

1

1

1263

4

1

1179

79

255475

8534

2

8532

14

14

246927

128

100

246699

119756

19077

19077

1270

359

6631

320

7475

3022

6465

56

56

6409

6409

107

14

14

93

93

302

302

4

298

48958

1557

21

24

1512

47401

43279

3860

1

6

255

36604

36604

36604

8243

8243

8243

1144

1

1

1

1

2

2

2

2

25

25

25

25

1116

1068

1068

1068

48

48

9

27

4

8

15695

746

746

14

14

732

1

729

2

10759

619

314

13

301

199

53

146

106

106

10140

10140

10140

1462

1462

1462

1462

2725

28

28

26

2

1114

970

839

131

110

110

34

34

1467

234

234

1226

933

293

7

7

116

116

116

3

3

3

3

242321

242321

242318

193305

6840

184494

1971

49013

45006

4007

3

3

3

16

15

15

15

2

13

1

1

1

1

39360

39360

39360

39360

39360

2

2

2

2

2

538

538

538

538

463

463

75

75

2894

2675

72

72

72

72

72

2603

2603

2603

2603

2603

35

30

30

30

30

30

5

5

5

5

5

184

87

87

87

87

87

97

97

97

97

97

595

595

568

568

568

568

568

27

14

14

14

14

13

6

6

6

7

7

7

0

0

0

0

0

0

0
