# Supplementary material for: Tree identity shapes composition, substrate shifts dominance in deadwood fungi of an old-growth lowland beech forest
Source: IMA Fungus. 2026 Aug 24;17:e190198. doi: 10.3897/imafungus.17.190198 (PMC13531028; doi:10.3897/imafungus.17.190198)
Supplement: Supplementary file 2 [file imafungus-17-e190198-s003.zip › krona_LOG_perSamplePct.html]

Javascript must be enabled to view this page.

magnitude
magnitudeUnassigned

krona\_LOG\_perSamplePct

15999991

29074

29074

29074

29074

29074

29074

29074

682

682

682

682

682

682

682

389

389

16

13

6

6

6

7

7

7

3

3

3

3

373

373

373

373

373

0

0

0

0

0

0

0

15957935

1202

550

550

550

550

550

26

26

26

26

26

107

107

104

104

104

3

3

3

464

464

6

6

6

458

458

348

110

6

6

6

6

6

49

49

49

49

49

52466

52466

52466

52466

52466

52466

80

70

70

70

70

70

10

10

10

10

10

8983

8983

8983

11

11

11

8972

1740

10

80

15

1541

94

722

722

5932

450

39

38

4640

12

280

297

1

137

38

301

301

5

5

137

137

135

135

162

162

162

162

162

162

7216869

379

160

160

160

160

219

219

219

219

2

2

2

2

2

122

25

25

25

25

94

55

55

55

39

39

27

8

4

2

2

2

2

1

1

1

1

63182

63182

63182

49883

46657

1948

1278

13299

3998

9301

77

77

77

77

77

9857

3

3

3

3

9854

9854

9854

5324

4528

2

7096081

38340

38340

42

42

38298

38298

83

83

4

4

77

77

2

2

164

164

164

164

1339221

2273

2273

2273

169389

431

431

7236

7236

6854

6854

154868

154868

192

192

3

145

41

3

6

4

4

2

2

210

210

210

66426

1763

1763

64663

64663

619285

151

24

127

96

96

191880

191880

76627

76627

350425

350425

106

106

13540

13540

6

13534

6847

6847

6847

19830

5

5

19825

19825

135971

135970

135970

1

1

299967

299967

4

134104

165859

5285

5285

5285

7597

592

537

537

50

50

5

5

7005

7002

7002

3

3

15

15

15

15

52655

19

19

19

24

24

24

12563

12563

1938

6620

320

3004

359

322

36455

35604

35604

851

851

14

14

14

3580

3580

3580

88639

88622

723

667

17

31

8

33

33

1057

1032

25

86799

86799

10

10

17

17

17

676

321

321

5

316

340

301

301

39

39

13

13

13

1

1

1

1

1

1

4814286

8186

13

13

3946

3832

9

59

46

5

5

2057

33

5

2019

2154

2154

11

11

278

278

278

605

605

605

2346265

934

934

12639

12639

2332692

304

2332388

111

111

95

16

21

21

21

14

14

14

151337

151337

151337

129497

330

330

124678

124678

150

150

4280

4280

3

3

56

56

29

29

29

1025

1025

5

5

19

128

4

65

720

79

1132925

897442

897442

196526

196526

38957

38957

418

418

418

89

89

89

1094

23

23

1071

1

86

984

4

4

4

539

539

539

781853

103

103

1

1

781431

243

1447

111414

1

215

3

4

262

379303

2233

286106

96

70

34

318

318

466

24

3

17

4

442

6

54

382

3

2

2

1

1

5301

5301

5301

218854

218484

218384

100

358

358

12

12

8481

356

356

8122

8122

3

3

82

82

82

26725

26703

26703

14

14

7

7

1

1

84

84

79

1

4

11631

2

2

2

11629

1622

1622

10007

10007

136871

136871

136871

136871

29358

2060

2039

1

2038

21

21

27173

16737

15

1006

351

8988

6377

10436

1

10435

125

80

33

47

45

45

271529

222801

222731

59608

131731

38

31354

64

64

6

6

48629

45526

1038

44488

1506

1506

1597

1597

1

1

1

94

94

94

4

4

4

297710

211

211

5

206

414

1

1

198

198

215

215

103755

103731

189

628

70737

32177

9

9

15

15

186504

65

65

32307

628

31679

153997

153997

135

135

6826

6826

6826

7306

7306

7306

7306

2

2

2

2

2

34901

5153

5153

5153

5153

1428

1428

1081

5

1076

342

4

338

5

5

339

339

7

7

332

332

21886

1341

44

44

1297

1297

20545

3417

3417

17027

14

17013

101

101

6095

10

10

10

1

1

1

1311

1311

1311

2948

2948

2948

158

158

17

141

327

2

2

60

60

265

265

2

2

2

1070

4

2

2

1066

761

303

2

268

268

263

5

742

163

163

163

2

161

569

569

26

26

543

341

202

3

3

3

3

7

7

7

7

187

65

65

65

65

122

2

2

2

120

81

2

79

39

28

11

11337

1

1

1

1

10059

576

306

6

300

78

78

192

51

141

9483

9483

9483

1267

440

270

4

266

170

170

6

6

6

26

26

26

795

795

62

733

10

10

10

10

47

47

47

47

47

47

53

31

31

31

2

2

29

10

19

22

22

22

22

22

41

37

37

37

37

37

4

4

4

4

4

532

532

532

532

75

75

457

457

6

6

6

6

6

6

8667617

44

44

3

3

3

41

30

30

11

11

87231

332

332

118

118

214

214

12760

12760

12760

12760

71193

9098

683

683

8406

8097

309

9

5

4

44489

7748

6535

125

15

1073

170

170

1414

1414

5224

56

5

5163

4

4

79

79

2516

317

559

341

97

78

4

3

3

1114

27334

8406

736

18

9353

5

8816

17413

17413

17413

193

188

188

5

5

2589

23

23

21

2

598

404

2

3

397

2

194

194

1968

1605

1

497

57

5

13

1

3

108

16

4

20

7

34

41

49

293

271

4

181

363

47

77

239

339

339

1

1

338

6

332

18

18

17

17

1

1

10723

10723

10723

10723

10723

11086

11086

1090

1090

1090

9996

57

57

903

3

20

802

78

14

14

849

849

8173

228

8

385

57

276

60

7159

939280

939280

40931

38376

38376

2555

2555

29278

2233

2044

189

27045

27045

711

711

711

859774

859774

859774

6319

8

3

5

11

11

1

1

7

7

6292

12

7

6273

521

521

216

305

1746

936

14

922

35

35

42

42

732

732

1

1

103528

10

10

10

10

14533

7212

1

1

509

509

6702

6702

7013

767

767

12

12

82

30

52

6152

6152

12

1

1

11

2

9

296

258

258

38

38

5

5

5

5

24832

24832

24832

24832

1

1

1

1

63

63

50

50

13

13

6438

6438

6438

6438

16

4

4

4

4

4

4

8

3

3

5

5

34

34

34

15

19

518

518

518

518

14422

1395

1395

1025

370

143

143

143

208

208

75

133

845

7

5

2

112

112

17

17

709

709

17

2

2

5

5

10

10

27

27

27

132

101

101

10

10

21

21

99

96

2

1

93

3

3

1600

180

180

793

793

576

235

7

301

33

51

51

184

184

184

80

80

80

2

2

2

210

210

210

49

49

49

7491

7491

7491

10

10

3

7

124

124

124

1

1

1

259

244

244

15

15

1429

65

55

10

1364

1364

88

88

88

29

29

29

28

28

28

28

42628

42612

42612

42612

16

16

16

4

4

2

2

2

2

2

2

1

1

1

1

1

129

129

129

21

21

108

73

26

9

662159

638128

7443

921

918

3

6522

6522

7

7

1

6

117530

2820

2820

2

2

4

4

71688

71688

42593

41834

143

616

376

241

135

47

47

84483

53

53

84227

57995

744

4844

24

20620

203

203

114

114

9

37

1

7

55

4

1

174497

174497

174445

52

264

264

264

10961

1221

1221

9740

66

10

2985

6679

6436

6436

6001

167

3

265

8

4

4

4

4

31114

31114

31114

171623

137784

75

3657

110366

23686

29632

29632

4207

7

4200

25803

25803

25612

191

24

24

24

2507

1955

1955

4

4

51

51

484

484

6

6

7

7

1930

116

116

101

85

16

1713

1713

43

43

13

30

46

19

19

27

27

3295

162

162

789

789

2344

2342

2

124

53

53

53

62

62

62

9

9

9

4611

4611

4611

4611

2376

2376

57

57

865

865

1454

1454

16920

27

27

27

16846

16062

16054

8

777

777

7

7

19

16

16

3

3

28

28

28

31141

2121

2121

1859

1859

122

122

38

38

102

102

29020

29020

29020

29020

1492

1492

406

406

391

15

1086

395

395

15

15

577

577

99

99

6807025

12648

12648

12648

39

9330

2545

734

4290

4288

4288

4288

2

2

2

178526

178526

78231

96

3

78132

2577

2577

4262

11

4251

10

10

75171

75171

18275

18275

2440

2440

2440

2440

13

13

13

13

269

203

203

203

66

66

66

297

297

297

297

2057

2057

323

323

1734

1734

66418

66418

66418

66418

358

358

312

312

45

6

39

1

1

88537

27192

27192

27192

511

511

8

503

201

201

154

47

21321

21321

21321

39312

39312

39312

266300

12683

34

34

409

409

4

4

151

63

88

294

294

33

33

11500

11500

258

63

41

154

13023

6

6

205

205

41

5

36

12765

12765

2

2

4

4

4

4

2

2

2809

94

2

92

2539

90

2449

28

28

130

23

107

18

18

6

5

3

2

1

1

164434

759

34

18

707

125835

125835

1

1

1236

1236

133

133

21816

9442

5

1192

638

10410

129

12

12

487

487

8464

1

7

8456

144

144

5547

168

5379

146

146

146

167

167

167

44336

3

3

44304

4923

109

22278

5

4

15730

2

1253

19

19

5

5

5

5

4

4

4

16098

16098

16098

12590

1354

108

442

804

2

2

11199

11199

1

1

28

28

6

6

35716

35716

55

55

20361

71

15299

4991

5591

2417

3174

9709

329

14

860

8506

24

24

24

24

74080

7849

7849

6473

1376

10199

10199

2129

8070

56032

6

6

51347

14929

36417

1

4679

4679

3626

3626

98

3

95

1693

168

12

1513

27

13

14

1808

1808

5586425

3633195

8257

24

8233

13

13

476

80

396

3624449

3624449

60

60

60

142820

126203

17482

76900

31821

16615

16615

2

2

385

385

385

4

4

4

452

452

452

1

1

1

29

29

29

1809479

66

66

1808407

1808319

88

955

955

51

51

229

229

229

229

421

4

4

4

401

2

2

390

390

8

8

1

1

16

16

16

1106

803

405

405

398

17

66

315

278

278

4

274

25

25

25

88877

88877

88877

87483

1394

93979

68420

68420

5

68415

25545

112

112

25433

25433

14

10

10

3

3

1

1

299335

299335

8570

8570

135287

135287

19

15

4

7471

4689

230

2514

38

11336

7

87

11242

4

4

134922

134922

1726

1726

760

239

239

239

521

521

20

501

294

294

294

294

13768

30

4

4

4

26

2

2

24

24

11148

11148

11148

11148

94

94

94

94

98

98

98

98

1229

301

301

24

84

26

30

137

130

130

130

669

559

367

181

11

110

3

107

1

1

1

128

128

128

1047

1047

1047

1047

10

10

10

10

10

10

10

10

102

102

102

102

4

4

4

4

4

2

2

2

2

2

9877

9221

9221

9221

9221

9221

656

456

456

456

456

146

146

146

146

54

54

54

54

813

594

72

72

72

72

72

522

522

522

522

522

184

97

97

97

97

97

87

87

87

87

87

35

30

30

30

30

30

5

5

5

5

5

11098

11098

533

533

533

533

533

85

85

85

85

85

9053

603

244

244

244

359

359

359

1477

1394

1394

1394

83

83

83

6973

6973

6973

6973

1427

1427

167

167

167

1260

1260

1260
