# Supplementary material for: Tree identity shapes composition, substrate shifts dominance in deadwood fungi of an old-growth lowland beech forest
Source: IMA Fungus. 2026 Aug 24;17:e190198. doi: 10.3897/imafungus.17.190198 (PMC13531028; doi:10.3897/imafungus.17.190198)
Supplement: Supplementary file 2 [file imafungus-17-e190198-s003.zip › krona_SNAG_perSamplePct.html]

Javascript must be enabled to view this page.

magnitude
magnitudeUnassigned

krona\_SNAG\_perSamplePct

2399999

0

0

0

0

0

0

0

192

192

192

192

192

192

192

2317743

1636806

1598

1598

1598

1598

1598

214

214

214

214

214

56

56

56

56

56

57555

19

19

19

19

467

467

467

467

2447

2447

2087

2087

360

360

10280

412

412

412

8749

178

178

802

463

339

199

58

141

80

80

6652

2520

751

1499

752

1130

34

34

785

5

31

331

233

43

142

19

19

1119

268

268

2

2

849

502

347

75

75

75

63

12

44231

32585

535

535

32050

1

42

20193

731

7681

957

406

15

289

560

1175

11646

4168

3512

656

7478

7478

36

36

27

27

9

9

5233

5233

55

1

1

54

54

19

18

18

1

1

33

33

33

417

417

417

2859

8

8

4

4

46

35

11

2801

20

423

2358

1850

1820

1820

4

4

5

5

21

21

527

527

2

2

2

525

54

54

471

443

9

7

12

215971

57854

57853

57853

57853

1

1

1

56

56

56

56

26008

2

2

2

2

2

2

32

32

32

5

5

5

19292

19292

18541

751

602

602

40

562

2

2

2

5260

5260

5260

214

66

66

136

136

3

3

9

9

21

19

19

2

2

208

1

1

207

144

63

7

7

7

3

3

3

312

312

312

44

44

5

39

2

2

2

118

118

55

55

63

63

11

11

11

11

3235

3235

3235

3235

128689

104

9

9

95

95

122010

4

4

2

2

121664

121664

340

340

6575

6575

1

6574

194

194

194

123

49

74

71

71

1150015

44509

44509

44509

44509

14785

14785

12370

11681

673

16

351

351

642

642

20

20

1402

1402

1258

1258

699

699

559

274

273

9

3

409

409

409

409

145

145

145

137

8

2144

2144

2144

2144

2088

2088

2088

2088

18261

18261

18261

18261

32

32

32

32

57933

29319

29319

29319

2413

2413

2413

22

22

22

3

3

3

26176

26176

26176

1536

1536

1536

1223

112

201

1603

1603

19

19

1584

243

30

1311

345

209

209

55

154

136

136

136

27574

27574

16442

16442

454

454

14

14

23

23

7100

7100

68

18

50

3473

3116

356

1

4

3

3

3

1

1

1

14039

14039

14039

5

14034

3586

3586

1214

1214

2372

1318

1054

132237

132237

52487

52487

72093

7818

64275

7657

6088

1569

4792

4792

1481

1481

1749

1742

7

1419

58

8

1353

143

25

3

115

19867

5

5

5

12558

12558

94

12464

19

19

19

2

2

2

49

2

2

47

47

7234

17

17

7217

7206

11

2247

2247

2247

2247

14156

188

90

90

98

25

71

2

4690

4690

4688

2

9278

3591

3591

5687

5687

624138

6

6

6

199724

146832

146832

34616

51

34565

18276

18252

24

318093

318077

257374

60703

16

16

1

1

1

106311

11157

11157

94717

5673

179

88865

437

437

3

3

3

161041

136

5

5

5

5

5

3

2

3

3

118

107

5

6

5753

102

102

5651

5474

76

101

4

2

2

2

2

67

67

5

62

30032

30032

30032

39396

39251

5

29161

10085

145

145

32451

13687

13687

2713

2713

4832

4760

72

4

4

51

51

11164

18

11146

51867

18564

18564

653

2

651

246

246

751

751

31653

2

31651

1230

1230

1230

88

88

88

17

1

1

16

16

1286

1282

1244

1244

31

31

2

2

2

2

3

3

4

4

4

794

119

119

119

119

9

9

9

9

666

666

666

666

204649

300

300

300

300

173958

17014

17014

17014

24127

19982

19982

2

2

1

1

209

209

3933

3933

2

1

1

1

1

536

5

5

531

531

107978

90

90

107888

520

77628

29740

1952

37

37

1907

1907

3

3

5

5

4

4

4

71

71

71

9804

174

163

11

9630

9630

180

180

180

517

517

513

4

2017

3

3

1

1

2013

2010

3

9756

3262

3262

6494

3135

3327

32

28350

28350

27921

27921

429

429

2

2

2

2

2039

2039

36

36

2002

2002

1

1

6071

6071

6071

6071

6071

6071

96

85

85

85

85

85

11

8

8

8

8

3

3

3

3

1308

1308

1308

1308

430

430

194

25

2

167

187

5

76

2

104

497

497

673407

639

4

4

4

4

635

635

635

621

14

635936

37378

11

11

11

37367

37367

37367

54

54

54

54

14282

12

12

12

1

1

1

6

6

6

14214

304

304

600

600

13310

6835

6475

49

49

1

15

33

3

3

3

3

3

3

3

3

227114

60168

60168

60168

7321

7254

7254

67

67

166

166

127

39

468

36

36

428

428

4

4

2611

2611

2611

98

98

98

2310

2310

7

2259

44

153972

5414

5414

148558

148558

422

422

422

422

74

74

74

74

260779

207

207

166

41

247

247

247

393

393

393

3

3

3

2842

2842

2

576

3

168

8

1934

151

5544

5373

5373

171

171

421

3

3

418

418

73946

73946

73946

117122

179

179

1235

1235

15849

15849

99859

1

99858

18

18

18

6258

6258

6258

9737

9733

128

9605

2

2

2

2

44041

43644

43644

397

397

420

420

420

420

85764

48

48

48

2602

2020

2020

582

582

83114

83114

946

82036

132

774

774

33

33

1

1

740

737

3

203

7

7

7

189

189

189

7

7

2

2

3

8666

558

558

558

4851

4851

255

790

1

3805

3257

3257

11

8

3231

7

366

60

55

55

55

5

2

2

3

3

108

33

33

6

27

2

2

2

73

73

73

198

198

198

198

1013

1013

1013

1013

1013

34546

34546

34543

34429

32096

2319

14

114

7

107

3

3

3

2

2

2

2

2

76

76

76

76

76

8

8

8

8

8

821

11

11

11

10

1

796

45

45

45

14

14

14

48

48

5

2

41

10

10

10

679

524

524

36

36

119

119

5

4

4

4

1

1

1

9

9

9

9

6

6

6

6

6

6

49

49

49

49

49

49

73568

73568

73568

73568

73568

73568

73568

20

20

20

20

20

20

20

8476

93

93

93

93

93

93

8383

1118

1118

1118

1118

1118

239

239

239

239

239

7026

7026

7026

7026

7026
