# Supplementary material for: Tree identity shapes composition, substrate shifts dominance in deadwood fungi of an old-growth lowland beech forest
Source: IMA Fungus. 2026 Aug 24;17:e190198. doi: 10.3897/imafungus.17.190198 (PMC13531028; doi:10.3897/imafungus.17.190198)
Supplement: Supplementary file 2 [file imafungus-17-e190198-s003.zip › krona_SOIL_perSamplePct.html]

Javascript must be enabled to view this page.

magnitude
magnitudeUnassigned

krona\_SOIL\_perSamplePct

1100009

165

165

165

165

165

165

165

47

47

47

47

47

47

47

0

0

0

0

0

0

0

1099783

4132

931

931

110

110

110

821

821

821

2016

2016

1997

1997

1

1996

19

19

19

13

13

13

13

13

91

91

91

91

91

292

292

292

292

292

789

789

789

789

789

440

440

440

432

274

274

158

65

93

8

8

8

6

6

6

6

6

6

816

816

816

816

816

816

315056

216

117

117

117

117

99

99

20

20

77

77

2

2

27412

27412

27412

27412

27412

567

567

180

10

10

18

18

2

2

3

3

12

12

135

135

387

387

387

132085

54193

11929

11

11

276

276

10152

402

102

2

11

1

2127

28

21

2802

568

73

3960

55

1176

24

1082

6

63

1

314

314

6823

378

3

375

780

780

962

7

955

198

198

13

13

47

3

17

27

2750

2750

285

285

3

3

1

1

28

28

114

114

1264

1264

11881

2506

2501

5

400

400

6

6

1533

2

609

922

7436

39

112

7285

299

1

1

23

23

2

2

18

18

3

3

55

47

8

137

137

38

38

22

22

14

14

14

5

5

5

683

683

683

337

337

337

15102

145

145

31

31

345

345

14581

13281

1269

31

179

179

179

6941

6660

6660

64

64

73

61

12

14

14

26

26

104

104

590

390

390

12

378

200

200

200

1648

1172

1172

127

1038

7

476

476

476

253

253

253

253

1405

1405

1257

168

630

272

187

148

148

44

44

12

12

11

11

18

15

3

3

3

19433

878

4

4

147

36

68

5

38

706

706

21

21

1741

1375

1375

366

2

25

339

7

7

7

124

124

124

50

50

5

45

16155

2

2

16153

16153

61

53

53

8

8

417

417

417

722

722

711

711

11

11

68

5

5

5

53

2

2

51

21

30

10

10

10

20

20

18

18

2

2

18

18

16

16

2

2

1

1

1

1

47

47

47

47

6

6

6

3

2

1

26706

26706

7093

171

6781

141

3

3

11106

11106

7682

67

7377

91

50

6

60

31

1

1

791

791

26

26

4

4

11367

1890

1890

409

1481

439

272

272

167

167

1671

360

360

2

2

1309

1309

48

48

48

2159

2159

2159

37

37

37

4038

4038

10

4028

1085

1085

1085

702

702

702

702

1269

1258

1258

22

46

1190

11

11

11

128

128

128

2

98

28

8337

8337

8337

8337

5076

7

7

7

582

7

7

514

514

6

6

28

28

24

24

3

3

4

4

4

4483

4483

4483

52

52

52

52

13283

2

2

2

2

214

214

214

214

75

65

65

65

10

10

10

345

345

345

345

1101

1065

15

15

8

2

6

1

1

971

971

6

6

19

12

7

3

3

42

42

8

8

8

11

11

11

16

16

16

1

1

1

25

25

25

25

373

351

351

351

22

22

22

2

2

2

2

66

66

66

66

10424

13

13

13

51

51

51

72

72

31

41

9

9

9

1732

1732

1732

43

43

9

34

6301

6301

6301

340

340

340

10

2

2

8

8

1

1

1

25

17

14

3

8

8

1

1

1

1

1

1

619

5

4

1

98

98

65

65

451

451

2

2

2

103

103

103

301

301

301

106

11

11

95

95

293

293

8

285

134

113

113

9

9

12

12

132

132

132

135

83

83

20

2

18

32

32

651

329

329

329

196

174

174

22

22

126

126

126

5

4

4

4

1

1

1

113248

7050

7050

12

12

87

87

349

349

6588

6588

14

14

1162

264

42

42

222

222

122

11

11

111

111

776

776

776

51

41

41

41

10

10

10

1399

1399

1399

1399

103079

23

23

23

241

208

208

17

17

16

16

10878

9183

9183

635

635

18

18

1042

1042

7608

1117

371

746

6247

6247

244

3

94

147

12741

12741

12741

1704

1704

1704

2947

2947

2947

20247

20247

20247

5023

136

2

128

6

1

1

4886

2

4825

59

459

66

66

393

393

229

107

107

122

23

94

5

15692

97

97

6

6

1004

1004

831

831

1620

1616

4

12134

12134

1831

103

103

1136

1136

51

51

541

541

4456

4456

4456

8844

8844

2

435

19

8

4024

2220

3

61

2072

303

7

7

98

98

198

182

16

9853

9853

9853

507

507

507

507

38

38

38

38

38

3205

3205

1881

1881

1881

1282

1282

1282

42

1

1

38

38

3

3

18273

5385

46

9

9

37

37

5330

163

163

2332

2332

189

18

131

40

524

524

204

196

8

1414

1023

391

504

1

35

438

2

28

9

9

9

15

15

15

15

7

2

2

2

5

5

5

3

3

3

3

12863

197

197

12

2

183

185

185

185

5929

62

62

3143

16

2

1

364

45

154

43

23

383

1925

93

52

9

6

27

2724

463

1

18

2242

6552

6552

6544

6

2

207

2

2

2

2

19

19

19

19

4

4

4

4

49

49

49

49

73

29

29

29

44

23

23

21

21

60

60

60

60

6513

6513

2712

13

13

2

2

371

49

244

78

77

77

2249

2249

3388

23

23

1940

1940

1425

1425

413

413

413

9

9

9

9

9

7138

3269

3269

3269

3269

3269

3869

34

34

34

34

38

38

38

38

2232

2232

2232

2232

1565

1565

1565

1565

757210

14

13

13

13

13

1

1

1

1

22

22

22

22

22

1

1

1

1

1

737123

232128

163567

163567

1

163566

66427

51412

51399

13

1

1

7723

7723

1

1

441

441

6838

6838

11

11

2

2

2

19

7

7

12

12

2113

2113

2113

12485

12485

4192

4192

8287

8287

6

2

4

242842

2

2

2

242421

43060

37488

4292

1280

199361

11692

108

62

2

94735

2072

8321

620

4812

1025

14659

1555

2101

300

32

57265

45

45

45

374

3

3

371

371

8

8

8

8

19816

79

79

79

19737

522

495

21

6

721

714

7

15

15

18479

18479

11372

4102

4102

4102

302

302

298

4

710

704

659

21

24

6

6

6251

6195

6195

56

56

7

7

3

4

175448

152

147

147

5

5

31

31

26

5

788

5

5

61

61

4

4

26

26

692

692

1467

1467

1467

42

42

15

27

265

6

6

25

25

234

1

233

23

15

2

13

3

3

3

3

2

2

25

14

14

1

1

7

7

3

3

53290

52989

1462

9

216

7

390

30

20

62

2360

48431

2

301

301

3182

3

3

3179

14

451

88

1875

750

1

1708

70

70

3

3

1635

1622

13

45

45

45

13780

13780

13780

4789

108

16

1

2

85

4

9

4

4

1

4346

2

1

4342

1

2

2

324

324

17

8

8

9

9

9

3

3

6

6

1180

1

1

1179

1179

2

2

2

19

6

6

13

13

154

150

141

9

4

4

22874

22874

110

705

614

21445

2208

2208

73

177

1750

208

20

20

20

68687

502

502

68185

68185

64

64

64

84

84

84

538

538

294

244

4

4

4

1

1

1

4795

15

15

1

14

806

806

806

3731

3731

3731

4

4

4

1

1

1

238

6

6

232

232

29294

19

19

19

27634

27634

27322

2

49

261

74

74

74

15

15

2

13

17

17

17

1534

55

55

563

563

905

905

11

11

1

1

1

2

2

2

2

5538

5538

5538

5538

1530

201

201

201

1329

1329

1329

82

82

82

82

641

577

577

577

11

11

11

45

45

13

32

8

8

8

1142

1142

14

14

1128

1128

28

28

28

28

27

1

335

335

335

335

335

17048

72

72

72

72

130

130

130

130

7860

1217

1217

1217

6

6

5

1

4381

4381

4381

90

90

76

14

109

109

109

2057

459

459

1598

1598

929

929

23

23

903

834

69

3

3

8057

2824

19

19

2805

2805

5233

5233

5226

7

34

34

34

34

34

2605

66

2

2

2

34

34

34

30

30

30

743

743

14

14

729

729

565

565

565

565

1231

1231

1231

1231

14552

14552

14552

42

42

42

14510

176

176

553

553

21

21

343

343

63

63

6805

6542

72

40

148

3

11

11

6538

94

361

143

757

36

4573

159

26

223

166

67

67

67

67

67

67

348

24

24

24

24

24

324

14

14

14

14

129

129

129

129

181

181

181

181

18

2

2

2

2

2

16

16

16

16

16

14

14

3

3

3

3

3

11

11

11

11

11
